# Picolinamide-functionalized macrocyclic chelators for ^203/212^Pb theranostic radiotracers

**DOI:** 10.1039/d6qi01035k

**Published:** 2026-06-11

**Authors:** Bradley E. Osborne, Christina Siakalli, Ryan K. Brown, Andrew J. P. White, Claudia Rocco, Dominik Weiss, Estefanía Delgado-Pinar, Enrique García-España, Jane K. Sosabowski, Michelle T. Ma, Nicholas J. Long

**Affiliations:** a Department of Chemistry, Imperial College London, Molecular Sciences Research Hub White City Campus London W12 0BZ UK n.long@imperial.ac.uk b.osborne18@imperial.ac.uk; b School of Biomedical Engineering and Imaging Sciences, King's College London, St Thomas’ Hospital 4th Floor Lambeth Wing London SE1 7EH UK; c Department of Earth Science and Engineering, Imperial College London South Kensington Campus London SW7 2BP UK; d Institute of Molecular Sciences, Universitat de València València Spain; e Centre for Cancer Biomarkers and Biotherapeutics, Barts Cancer Institute, Queen Mary University of London London UK

## Abstract

Picolinamide-functionalized macrocyclic ligands represent a promising class of chelators for ^203/212^Pb-based theranostic applications, offering a dual role in both diagnostic imaging and targeted radiotherapy. In this study, two 18-membered diazacrown ligands, K22_PicAm and the novel NPK_PicAm, were synthesized and complexed with both non-radioactive Pb^2+^ and radiotherapeutic ^212^Pb^2+^. Structural characterization *via* NMR spectroscopy and X-ray diffraction confirmed the formation of a single, highly rigid, symmetric [Pb(K22_PicAm)]^2^^+^ species. Density Functional Theory (DFT) and Natural Bond Orbital (NBO) analysis indicated stereochemically inactive 6s^2^ lone pairs in the Pb^2+^ complexes, leading to holodirected geometries. UV-vis spectroscopy and potentiometric titrations showed both ligands form highly stable Pb^2+^ complexes, with complete binding by K22_PicAm and NPK_PicAm between pH 4 and 9. Radiolabelling studies with ^212^Pb demonstrated near-quantitative radiochemical conversion within 15 minutes. These results establish picolinamide-bearing macrocycles as promising candidates for the development of next-generation, targeted ^203/212^Pb theranostic agents and support their further exploration in radiopharmaceutical research.

## Abstract 2

Picolinamide-functionalized macrocyclic ligands represent a promising class of chelators for ^203/212^Pb-based theranostic applications, offering a dual role in both diagnostic imaging and targeted radiotherapy.

## Graphical abstract

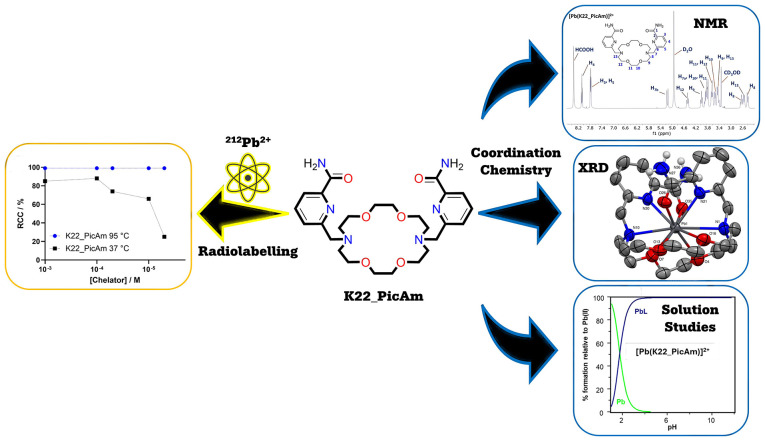

## Introduction

Lead(ii)-based (Pb^2+^) radiopharmaceuticals have gained significant interest in oncology due to their unique properties, offering a promising dual role in both diagnostic imaging and targeted radiotherapy.^1–3^ Theranostic “look and treat” radiopharmaceuticals use pairs of radionuclides that are incorporated into the same or similar molecular architectures that target receptors selectively expressed on the surface of tumour cells. Indeed, these molecular pharmaceuticals have transformed treatment outcomes for many cancer patients, particularly in neuroendocrine and prostate cancer.^4,5^ The first diagnostic radiopharmaceutical enables tumour imaging using either Positron (β^+^) Emission Tomography (PET), or Single Photon Computed Tomography (SPECT), selecting patients who are eligible for the second, systemic therapeutic radiopharmaceutical, which emits cytotoxic alpha- (α), beta- (β^−^), or Auger particles.^6–9^ Either same-element isotope pairs (“true theranostics”) or different-element isotope pairs can be employed.^10,11^ For example, receptor-targeted peptide derivatives incorporating the PET radionuclide, gallium-68 (^68^Ga, *t*_1/2_ = 68 minutes) are used to stratify patients for companion receptor-targeted radiotherapies containing β^−^-emitting lutetium-177 (^177^Lu, *t*_1/2_ = 6.65 days).^12^ Same-element isotope pairs, such as lead-203 (^203^Pb, *t*_1/2_ = 51.9 hours) and lead-212 (^212^Pb, *t*_1/2_ = 10.6 hours) are appealing as they enable a “true” molecular match, with the diagnostic agent having an identical chemical structure to the radiotherapeutic agent. ^203^Pb is a suitable SPECT radionuclide; significantly, therapeutic ^212^Pb emits both β^−^-particles, and an α-particle *via* the decay of its daughter nuclide bismuth-212 (^212^Bi, *t*_1/2_ = 60.6 minutes).^2,13–17^ Preclinical studies with ^203^Pb- and ^212^Pb-based radiopharmaceuticals have demonstrated promising tumour targeting and therapeutic efficacy in pancreatic, melanoma, prostate, ovarian, and breast cancer models.^18–29^ Notably, ^212^Pb-radiopharmaceuticals are now advancing through early-phase clinical trials, showing favourable biodistribution and safety profiles in prostate, ovarian, and breast cancer.^30–35^

**Fig. 1:**
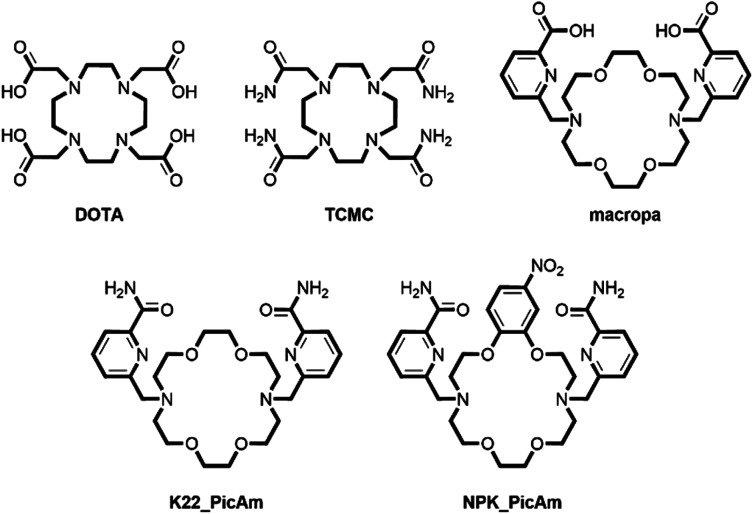
The structures of the chelators discussed in this work.

**Scheme 1:**
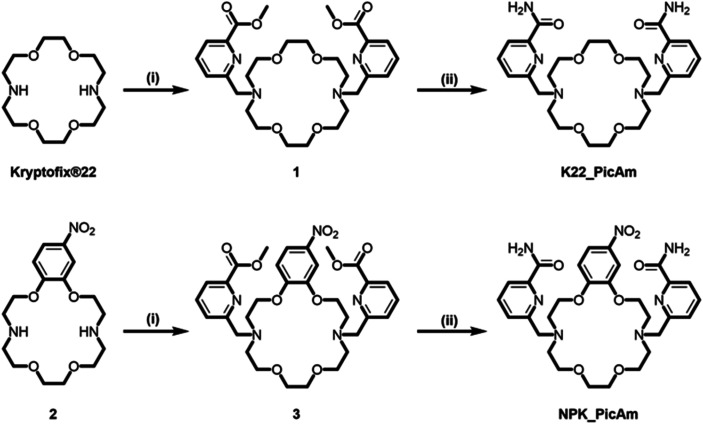
The synthesis of chelators K22_PicAm and NPK_PicAm. Reaction conditions: (i) methyl 6-(chloromethyl)picolinate, Na_2_CO_3_, MeCN, reflux, silica gel chromatography; (ii) NH_3(aq)_ (35 wt%), 0 to 25 °C, reverse-phase chromatography.

Chelators are critical to radiometal-based radiopharmaceuticals, as their resulting complexes need to possess sufficient thermodynamic and kinetic stability to deliver payload to diseased tissue *in vivo*.^36,37^ DOTAM (TCMC, Fig. 1) is cited as the “gold-standard” for Pb^2+^-based radiopharmaceuticals: its intermediate Lewis basic amides are more suitable for Pb^2+^ coordination compared to the analogous carboxylates of the prevalently used chelator, DOTA (Fig. 1).^36,38,39^ Alongside DOTA and DOTAM, which are prevalently used in radiopharmaceuticals, several families of macrocyclic chelators have also been investigated for the complexation of Pb^2+^ radionuclides.^40–46^ In particular, macropa (Fig. 1), an 18-membered macrocyclic chelator with picolinic acid pendant arms,^47^ has demonstrated exceptional selectivity and radiochemical labelling efficiency with Pb^2+^.^44,48^ We hypothesized that substituting the picolinic acid pendant arms with picolinamide groups would further enhance the kinetic inertness and thermodynamic stability of the Pb^2+^ complexes. Although not yet reported for radiopharmaceutical purposes, K22_PicAm (Fig. 1) was first used for the complexation of Eu^2+^ to investigate its size-discrimination ability and redox stabilisation.^49^ Here, we report the synthesis and characterisation of novel Pb^2+^ complexes of K22_PicAm, including a radiolabelled ^212^Pb^2+^ complex. We additionally report a novel nitrophenyl analogue of K22_PicAm, NPK_PicAm (Scheme 1), as a potential precursor to enable functionalisation of K22_PicAm with biomolecules (*e.g.* peptides) for targeting receptors of cancer cells, *via* the nitrophenyl motif.^50^

## Results and discussion

### Synthesis of the chelators

K22_PicAm was synthesised in 21% yield from commercially available Kryptofix®22 by following a previously reported procedure^51^ (Scheme 1). NPK_PicAm was synthesised in a similar manner to K22_PicAm in two steps. Refluxing compound 2 ^52^ in anhydrous acetonitrile with 2.1 equivalents of methyl 6-(chloromethyl)picolinate, and 4 equivalents of sodium carbonate gave compound 3 as an orange oil. NPK_PicAm was obtained by aminolysis of 3, as a yellow solid in 38% yield after purification.

### Solution-state structures of Pb^2+^ complexes

**Fig. 2:**
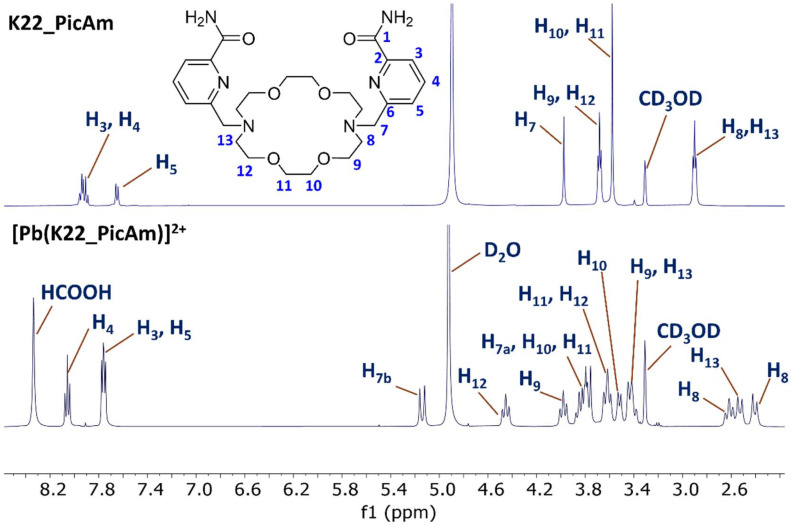
^1^H NMR spectra of K22_PicAm, and [Pb(K22_PicAm)]^2+^, recorded in MeOD (400 MHz, 298 K).

**Fig. 3:**
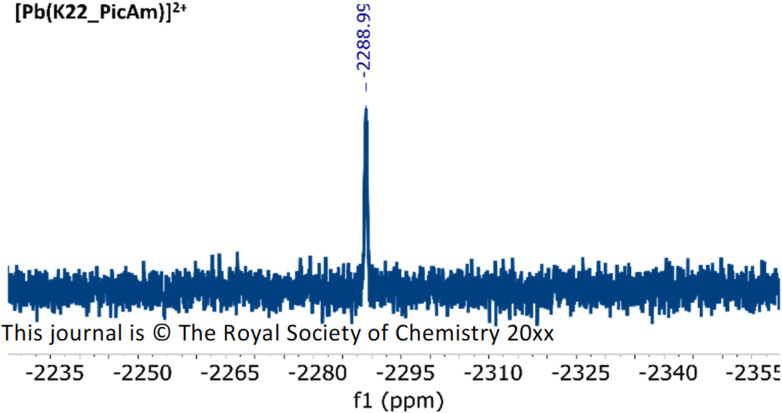
^207^Pb{^1^H} NMR spectrum of [Pb(K22_PicAm)]^2+^, recorded in D_2_O (84 MHz, 298 K).

Pb^2+^ complexes were synthesised by the addition of 1.1 eq. of Pb(OAc)_2_·3H_2_O to a solution of the chelator in an aqueous NH_4_OAc solution (pH 4.5) at room temperature. After stirring for 15 minutes, the mixture was purified *via* reverse-phase chromatography, and the desired complexes characterised *via* NMR and MS (Fig. S13–S17, S19, S20, S24 and S25, SI). These complexation conditions were repeated in MeOD to record the complete reaction system and to assess the behaviour of the Pb^2+^ complexes in solution (Fig. 2). The ^1^H NMR spectrum of [Pb(K22_PicAm)]^2+^ is characteristic of a single highly rigid *C*_2_ symmetric species, evidenced by the presence of well-resolved aliphatic proton resonances, in contrast to other polyether complexes which are known to exhibit dynamic and fluxional behaviour.^43,53^ Further, geminal AB type splitting patterns are observed for all methylene and ethylene protons in the Pb^2+^ complex. The presence of a single, highly rigid species suggests that this complex could possess the requisite stability that is critical for *in vivo* applications. The ^207^Pb NMR spectrum of the complex [Pb(K22_PicAm)]^2+^ displays a single signal at −2289 ppm (Fig. 3 and Fig. S19, SI), *versus* Pb(NO_3_)_2_, which is within the range of macrocyclic crown systems with carboxylic acid and amide pendant arms reported previously (−2076 to −2299 ppm).^40,41^ Pb^2+^ complexation experiments with NPK_PicAm were undertaken by reacting 1 eq. of Pb^2+^ with 1 eq. of NPK_PicAm at 25 °C. ^1^H NMR, HRMS (ES-TOF+) and HPLC analyses (Fig. S20, S25, S29 and S30, SI) indicated the formation of a single product, consistent with the formation of [Pb(NPK_PicAm)]^2+^. The ^1^H NMR spectrum of [Pb(NPK_PicAm)]^2+^ was more complex than that of [Pb(K22_PicAm)]^2+^ (Fig. 2) due to the relative lower symmetry of the former, which arises from the incorporation of a nitrophenyl group. However, compared to [Pb(K22_PicAm)]^2+^, similar patterns in chemical shift changes were observed upon coordination of NPK_PicAm to Pb^2+^. HRMS (ES-TOF+) analysis showed the expected *m*/*z* signals for a complex of formula [Pb(NPK_PicAm)]^2+^. HPLC analysis showed the formation of a single Pb-bound species, with a retention time distinct to that of the free ligand.

### X-ray crystal structure of the [Pb(K22_PicAm)]^2+^ complex

**Fig. 4:**
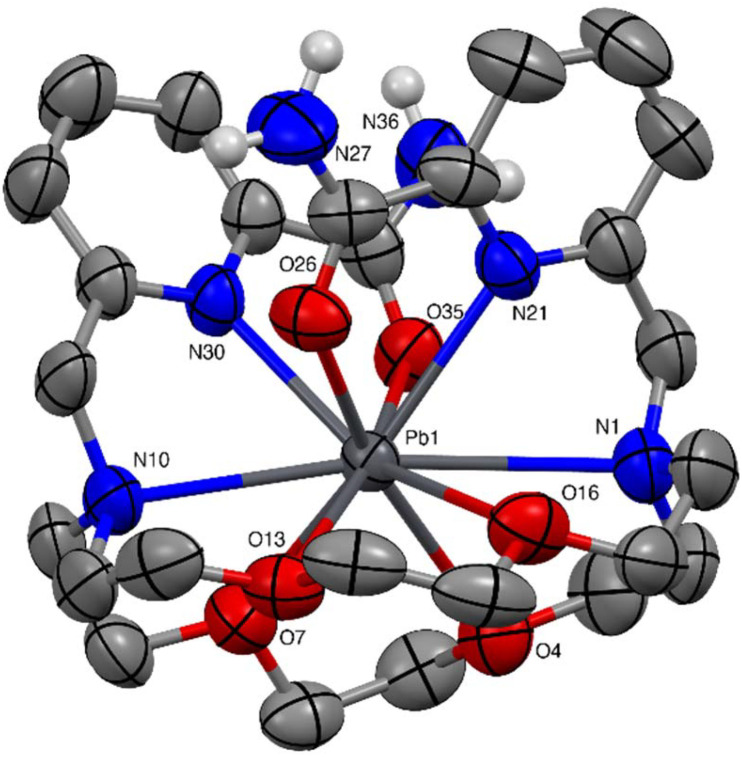
The structure of the cationic complex present in the crystal of [Pb(K22_PicAm)]-2PF_6_. Thermal ellipsoids are drawn at the 50% probability level. Hydrogen atoms and both PF_6_ anions are omitted for clarity.

Single crystals of [Pb(K22_PicAm)]-2PF_6_ suitable for X-ray diffraction studies (Fig. 4 and Fig. S41, Table S2, SI) were obtained. Bond distances and angles of the Pb^2+^ coordination environments are listed in Table 1 and Table S3, SI. Compound [Pb(K22_PicAm)]-2PF_6_ crystallizes in the tetragonal *I*4/*m* space group, with the crystal containing two non-coordinated PF_6_^−^ anions. The Pb^2+^ metal ion in [Pb(K22_PicAm)]-2PF_6_ is ten-coordinated by the chelator, which binds through the two N and four O donor atoms of the macrocycle, two pyridyl N atoms, and two amide O atoms.^47^ The macrocyclic Pb–N and Pb–O distances (Table 1 and Table S3, SI) range from 2.89 to 3.05 Å, whereas pyridyl Pb–N distances (2.61 to 2.68 Å) are shorter. Amide Pb–O distances are shorter again (2.53 to 2.60 Å). The structure of [Pb(K22_PicAm)]-2PF_6_ displays shorter bond lengths involving donor atoms of the picolinamide pendant arms, whereas the donor atoms of the macrocyclic unit fall within a longer bond length range. This indicates that the donor atoms of the picolinamide pendant arms provide the strongest interactions with the Pb^2+^ ion. A similar trend in bond lengths was reported for the Pb^2+^ complex of macropa, in which the shortest bonds were observed between picolinate N and O donor atoms and Pb, with longer bond distances – similar to those observed here for [Pb(K22_PicAm)]-2PF_6_ – reported between macrocyclic N and O donors and Pb.^47,48^ An alternative trend in coordination of Pb^2+^ to multidentate macrocyclic chelators has also been reported, in which donor atoms of the macrocycle form stronger interactions with the Pb^2+^ metal ion compared to donor atoms of the pendant arms. This type of coordination has been observed in the Pb^2+^ complexes of the cyclen derivatives DOTA, DOTAM and THP-12-ane-N_4_, along with other azacrown macrocycles,^39,41,54–56^ although these structures are all considered to be hemidirected.

**Table 1:** Bonding distances (Å) of the Pb^2+^ coordination environments in the X-ray structure of [Pb(K22_PicAm)]-2PF_6_ shown in Fig. 4

	[Pb(K22_PicAm)]-2PF_6_
Pb(1)–O(26)	2.534(4)
Pb(1)–O(35)	2.604(4)
Pb(1)–N(30)	2.613(5)
Pb(1)–N(21)	2.679(5)
Pb(1)–N(10)	2.890(5)
Pb(1)–O(7)	2.891(4)
Pb(1)–O(16)	2.910(4)
Pb(1)–O(13)	2.925(4)
Pb(1)–O(4)	3.015(4)
Pb(1)–N(1)	3.045(5)

### DFT calculations

Having the electronic configuration [Xe]4f^14^5d^10^6s^2^, Pb^2+^ is one of the post-transition metal elements that exhibits the “inert pair effect”.^57^ The resulting lone pair of electrons are stereochemically active or inactive. Such Pb^2+^ complexes are termed as holodirected (inactive lone pair, even donor atom distribution) or hemidirected (active lone pair, uneven distribution of donor atoms), with the latter exhibiting lower stability under certain conditions.^58^ Hemidirected complexes are favoured by low coordination numbers, and hard and charged donor atoms, whereas holodirected complexes prefer high coordination numbers and soft donor atoms.^59^ Natural Bond Orbital (NBO) analysis can quantify the 6s^2^ lone pair character of Pb^2+^ complexes by analysing electron density distribution, orbital interactions, and hybridisation. If the lone pair has significant s-character with minimal 6p orbital mixing, it is likely to be stereochemically inactive, favouring holodirected geometry. Using DFT, NBO analysis of [Pb(K22_PicAm)]^2+^ and [Pb(NPK_PicAm)]^2+^ indicates that the Pb^2+^ lone pair is stereochemically inactive in both complexes due to the insignificant 6p contribution (0.59% and 0.60% respectively) to the 6s^2^ lone pair (Fig. S42–S45 and Table S5, S6, SI), signifying both complexes are holodirected. Initial geometries for K22_PicAm and NPK_PicAm and their respective Pb^2+^ complexes were taken from the crystal structure of macropa with modification of picolinate arms to picolinamide arms and the addition of a nitrophenyl group in the macrocyclic ring.^48^ The optimized structures of the Pb^2+^ complexes are consistent with the X-ray crystal structure [Pb(K22_PicAm)]-2PF_6_ in which there is no distinct void in the Pb^2+^ coordination sphere, indicative of a stereochemically inactive lone pair, supporting the presence of a holodirected complex.

### Protonation constants and thermodynamic stability of the Pb^2+^ complexes

To investigate the properties and affinity of the new ligands for Pb^2+^, potentiometry and UV-Vis spectroscopy were used to determine the protonation constants (*K*_i_) and the Pb^2+^ stability constants (*K*_PbL_) (Table 2). Consistent with previous reports,^49^ we find that potentiometric measurements resolve only two protonation constants for the systems studied. The second protonation constant (log *K*_H2_) determined in this study is in good agreement with literature values, while some variation is observed for the first constant (log *K*_H1_), which we attribute to differences in ionic strength and electrolyte. Comparison with the NPK_PicAm, for which potentiometric measurements provided well-defined constants (Table 2), supports the assigned value for the first protonation constant of K22_PicAm (log *K*_H1_ = 8.30). The observed UV-Vis absorption spectra suggest the presence of two different protonation processes. At acidic pH, the absorption spectrum displays a band centred at 270 nm, with shoulders at 262 and 276 nm. Upon gradual addition of NaOH(aq), the band broadens slightly and absorbance decreases until pH 5.6, followed by an increase in absorbance until pH 9.0, when a plateau is reached (Fig. S37, SI). The decrease in absorbance in acidic pH might correspond to the deprotonation of the pyridine nitrogen atoms while the increase in absorption correspond possibly to deprotonation of the tertiary ammonium groups of the macrocycle.^60^ While our observed UV-Vis trends are consistent with multiple protonation sites, the combined spectroscopic, potentiometric and literature data support a model in which the dominant observable equilibria correspond to the tertiary amine groups of the macrocyclic ring.^41,54^

**Table 2:** Protonation and Pb^2+^ complex formation constants for the ligands K22_PicAm and NPK_PicAm (L)

	Reaction	K22_PicAm		NPK_PicAm
Log *K*_H1_	H^+^ + L ⇆ HL^+^	7.90(2)^a^	7.08^c^	7.82(1)^a^
Log *K*_H2_	H^+^ + HL^+^ ⇆ H_2_L^2+^	6.43(1)^a^	6.40^c^	5.422(7)^a^
Log *K*_PbL_	Pb^2+^ + L ⇆ [PbL]^2+^	14.19(2)^b^	13.28^d^	11.68(4)^a^
Log *K*_PbLOH_	Pb^2+^ + L ⇆ [PbL(OH)]^+^ + H^+^			0.97(4)^a^
pPb		14.5	14.0	12.1

a Determined by potentiometric titration in 0.15 M NaCl at 25 °C in this study.

b Determined from spectroscopic titration data and using protonation constants obtained in this study.

c Determined by potentiometric titration in 0.1 M KCl at 25 °C in a study reported by Regueiro–Figueroa *et al.*^49^

d Calculated from spectroscopic titration data obtained in this study and using protonation constants reported in the literature.^49^ pPb corresponds to the negative logarithm of the equilibrium concentration of uncomplexed Pb^2+^ in the presence of the ligand at pH 7.4.

Potentiometric measurements indicate that complex formation occurs predominantly in a pH range where competition from protons is minimal, making the extraction of reliable stability constants (log *K*_PbL_) from these data challenging. Therefore, spectroscopic titrations were performed using solutions containing Pb^2+^ and K22_PicAm in a 0.9 : 1.0 molar ratio, titrated with aqueous NaOH starting from pH < 2 (Fig. S38, SI). Pb^2+^ complex formation is observed at low pH (approx. 2, see Fig. S38, SI). Absorption at 276 nm increases progressively with a maximum at pH 3.6, indicating quantitative formation of [Pb(K22_PicAm)]^2+^ ([PbL]^2+^). At pH above 10, absorbance slightly increases suggesting the formation of a minor hydroxide species [PbL(OH)]^+^. To determine the stability constant for the Pb-L complexes, a chemical model was developed using protonation constants derived from potentiometric measurements conducted during this study and a previous study.^49^ The spectrophotometric experimental titration data was fitted using the software program HypSpec.^61^ We find for the formation of a [PbL]^2+^ species log *K*_PbL_ values ranging from 13.28 to 14.19, indicating quantitative complexation of lead at pH 7.4.

Both ligands achieve quantitative Pb^2+^ binding under mildly acidic conditions (pH 3–5) and K22_PicAm exhibits a higher apparent stability compared to NPK_PicAm and pPb for NPK_PicAm is two logarithmic units lower.

**Fig. 5:**
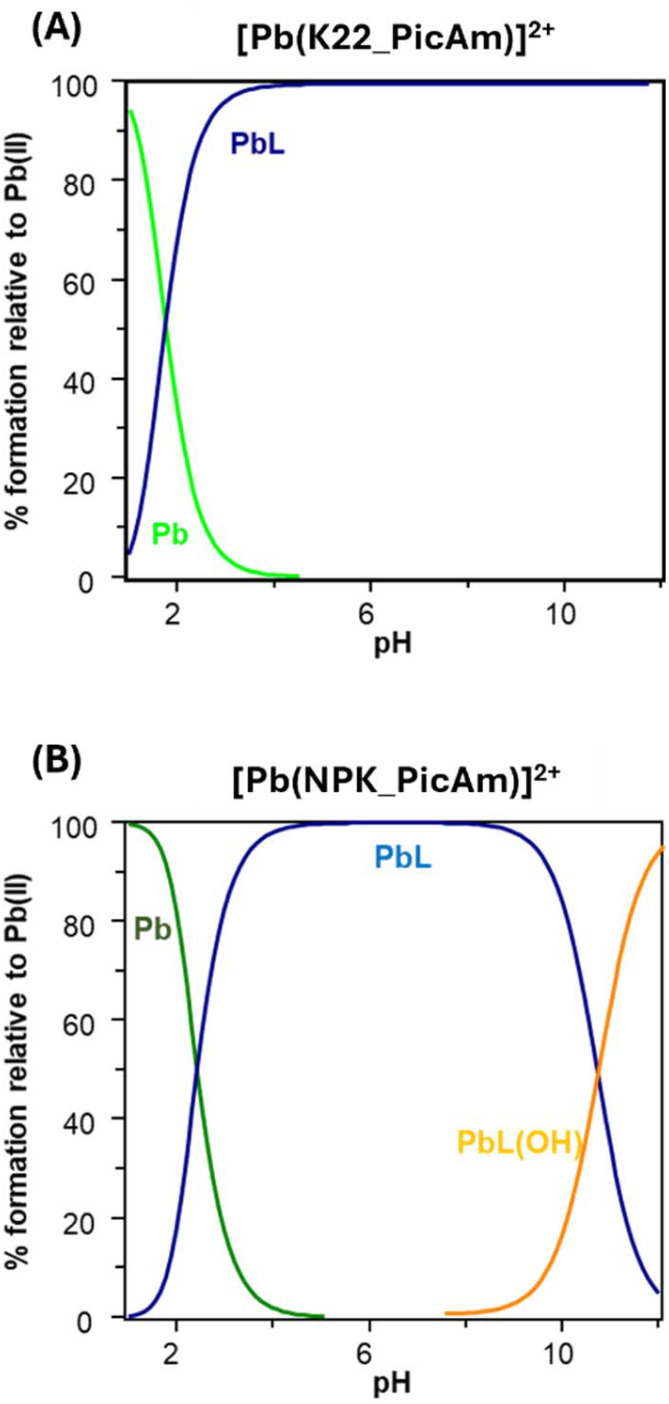
Speciation diagrams of the system Pb^2+^-L. (A) K22_PicAm; (B) NPK_PicAm.

The incorporation of a rigidifying and electron-withdrawing nitrophenyl group into the macrocyclic backbone results in an expected decrease in thermodynamic stability. The speciation diagrams (Fig. 5) indicate that under the conditions tested here, all Pb^2+^ in solution is bound to the chelator, either K22_PicAm and NPK_PicAm, above pH ≈ 4 and below pH ≈ 9. Notably, this pH range is well aligned with conditions commonly employed for ^203^Pb/^212^Pb radiolabelling, and the efficient complex formation observed in this work is consistent with the results shown in the ^212^Pb labelling discussed below.

### Lead-212 radiolabelling studies

**Fig. 6:**
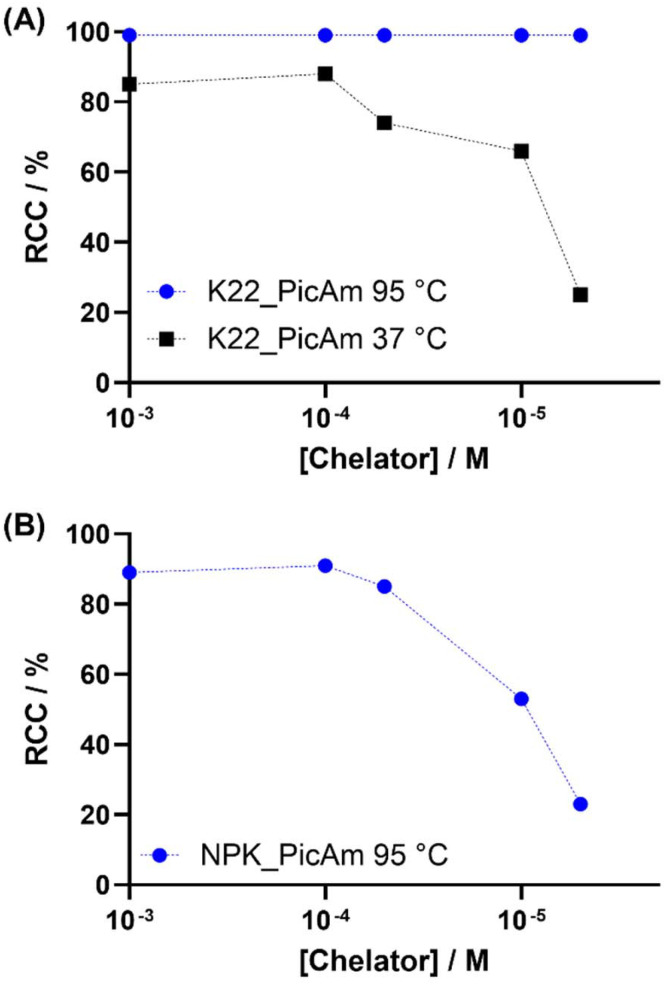
The radiochemical conversion (RCC) values for [^212^Pb]Pb^2+^ labelling with (A) K22_PicAm at chelator concentrations 1 mM to 5 µM over 15 minutes at 37 °C and 95 °C, and (B) NPK_PicAm at chelator concentrations 1 mM to 5 µM over 15 minutes at 95 °C.

Radiolabelling reactions of K22_PicAm and NPK_PicAm with [^212^Pb]Pb^2+^ were undertaken. In these reactions, a solution of [^212^Pb]Pb(OAc)_2_ (∼1 MBq) was added to a solution of chelator and reacted for 15 minutes at either 37 °C or 95 °C, with the final reaction containing the pertinent chelator at a concentration of 1 mM, 100 µM, 50 µM, 10 µM or 5 µM, at pH 5.5, in 0.5 M NaOAc buffer. Radiochemical conversions (RCCs) were assessed using radio-iTLC and are summarised in Table S1, SI. Upon heating at 95 °C for 15 minutes, K22_PicAm produced near quantitative RCCs across all chelator concentrations (Fig. 6A). At 37 °C, and a chelator concentration of 100 µM, a RCY of 88% was achieved, and at a chelator concentration of 10 µM a RCC of 66% was achieved. Comparatively, for NPK_PicAm a steady decrease in RCC values was observed as the chelator concentration was decreased (Fig. 6B). At a chelator concentration of 1 mM (10^−3^ M), NPK_PicAm achieved a RCC value of 85%, whereas at a lower chelator concentration of 5 µM (5 × 10^−6^ M) a RCC value of 23% was achieved. Due to the lower RCC observed for NPK_PicAm at high temperature, we did not investigate [^212^Pb]Pb^2+^ labelling with NPK_PicAm at lower temperatures. These preliminary findings underscore the potential of the K22_PicAm ligand system as a foundation for developing Pb^2+^-based bifunctional chelators, paving the way for future structural refinements tailored to a broad spectrum of biological targeting vectors.

## Conclusions

In summary, we report two 18-membered diazacrown macrocyclic chelators bearing picolinamide pendant arms, K22_PicAm and NPK_PicAm for ^212^Pb radiopharmaceuticals. Structural NMR and X-ray diffraction studies show that [Pb(K22_PicAm)]^2+^ forms a single, highly rigid, symmetric species, and UV-Vis spectrophotometric and potentiometric titrations showed that both K22_PicAm and NPK_PicAm form highly stable Pb^2+^ complexes. Radiolabelling each of these chelators with ^212^Pb^2+^ achieved near-quantitative radiochemical conversion within 15 minutes at high temperatures.

As metallic radionuclides, such as ^212^Pb, with therapeutically efficacious decay profiles become available for clinical application, chelator technologies such as K22_PicAm and NPK_PicAm are critical to enable their therapeutic application, to benefit patients. Recent first-in-human translational studies of radiotherapeutic ^212^Pb agents highlight the significant potential of K22_PicAm and NPK_PicAm chelators as chemical platforms for the development of ^203/212^Pb-based theranostic agents. Further functionalisation of these chelators with receptor-targeted motifs will transform the K22_PicAm scaffold into a precision-guided tool for application as a molecular theranostic agent in combination with lead radionuclides.

## Author contributions

BEO synthesised the compounds, performed the analyses, carried out the radiolabelling, and DFT calculations. X-ray crystallography was performed by RKB. Potentiometric titrations were performed by CS. UV-Vis spectrophotometric titrations were performed by ED-P and EG-E. MTM and NJL supervised the project, and all the authors contributed to the writing of the manuscript.

## Conflicts of interest

There are no conflicts to declare.

## Supplementary Material

QI-013-D6QI01035K-s001

QI-013-D6QI01035K-s002

## Data Availability

All the relevant research data is contained with the manuscript and supplementary information (SI). Supplementary information is available. See DOI: https://doi.org/10.1039/d6qi01035k. No databases have been used and no references to such databases are contained in the manuscript or SI. CCDC 2432138 contains the supplementary crystallographic data for this paper.^62^
