# Supplementary material for: Picolinamide-functionalized macrocyclic chelators for 203/212Pb theranostic radiotracers
Source: Inorg Chem Front. 2026 Jun 11;13(14):6289–97. doi: 10.1039/d6qi01035k (PMC13277825; doi:10.1039/d6qi01035k)
Supplement: QI-013-D6QI01035K-s001 [file QI-013-D6QI01035K-s001.pdf]

Supplementary Information (SI) for Inorganic Chemistry Frontiers.
This journal is © the Partner Organisations 2026

Supplementary Information

Picolinamide-functionalized macrocyclic chelators for $^{203/212}$Pb theranostic radiotracers

Bradley E. Osborne,*[a,b] Christina Siakalli,[a,b] Ryan K. Brown,[a] Andrew J. P. White,[a] Claudio Rocco,[c] Dominik Weiss,[c] Estefanía Delgado-Pinar,d Enrique García-España,[d] Jane K. Sosabowski,[e] Michelle T. Ma,[b] and Nicholas J. Long*[a]

a. Department of Chemistry, Imperial College London, Molecular Sciences Research Hub, White City Campus, London, W12 0BZ, UK. E-mail: n.long@imperial.ac.uk and b.osborne18@imperial.ac.uk
b. School of Biomedical Engineering and Imaging Sciences, King's College London, 4th Floor Lambeth Wing, St Thomas' Hospital, London, SE1 7EH, UK.
c. Department of Earth Science and Engineering, Imperial College London, South Kensington Campus, London, SW7 2BP, UK.
d. Institute of Molecular Sciences, Universitat de València, València, Spain.
e. Centre for Cancer Biomarkers and Biotherapeutics, Barts Cancer Institute, Queen Mary University of London, London, UK.

Contents

1. General considerations 2
2. Chelator Synthesis 2
3. Pb^{2+} Complex Formation 4
4. Radiochemistry 5
5. Radio-TLC Chromatograms 6
6. NMR Spectroscopy 8
7. Mass Spectra 16
8. HPLC Spectra 19
9. Potentiometric Titration and UV-Vis Spectroscopy Measurements 23
10. Crystal Data and Structure Refinement Details for [Pb(K22_PicAm)]-2PF_6 30
11. Density Functional Theory (DFT) Calculations 35
12. References 48

1. General considerations

Reagents: All solvents were purchased from commercial suppliers (Sigma-Aldrich, Fischer Scientific, TCI America, Fluorochem Ltd) and were used without further purification. All gas mixtures were purchased from BOC Industrial Gases. Anhydrous solvents were acquired from a Grubbs type SPS system and stored over activated 3 Å molecular sieves under inert atmosphere.

Instruments and characterisation: ^{1}H, ^{13}C and ^{207}Pb NMR spectra and 2D experiments (e.g., COSY, HSQC) were obtained using Bruker AV-400, AV-500, and DR-400 spectrometers. Chemical shifts (δ) are reported in ppm relative to residual undeuterated solvent signals ($CDCl_3$ δ_H = 7.26 ppm, δ_C = 77.16 ppm; D_2O δ_H = 4.79 ppm; CD_3OD δ_H = 3.31 ppm, δ_C = 49.00 ppm). The following abbreviations are used to designate multiplicities: s = single, d = doublet, t = triplet, q = quartet, m = multiplet, quint = quintet, br = broad. Coupling constants (*J*) are reported in hertz (Hz). NMR spectra were analysed using MESTRELAB MestReNova software. High resolution mass spectra were recorded using a Waters LCT Premier (ESI) spectrometer by the Imperial College Department of Chemistry Mass Spectrometry service, or independently on an Agilent 6200 TOF LC-MS instrument. Reverse-phase purifications were carried out on an Isolera™ 4 System using Biotage® Sfär Ultra C18 cartridges (12 g), and gradients are indicated throughout the text. Thin Layer Chromatography (TLC) was performed using TLC silica gel 60 F254 (aluminium sheets 20 × 20 cm).

Analytical HPLC chromatograms were obtained using an Agilent 1200 series instrument equipped with a flow-ram detector (Lablogic, Sheffield, UK), and integrated using Laura 6 software (Lablogic, Sheffield, UK). Columns, flow rate, and mobile phases are indicated in the text.

2. Chelator Synthesis

6,6'-((1,4,10,13-tetraoxa-7,16-diazacyclooctadecane-7,16-diyl)bis(methylene))dipicolinate (**1**) and 18-nitro-3,4,5,6,8,9,12,13,14,15-decahydro-2H,11H-benzo[b][1,4,10,13]tetraoxa[7,16] diazacyclooctadecine (**2**) were prepared according to previously reported procedures.[1,2]

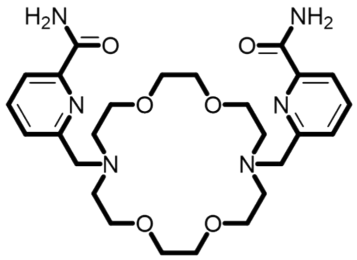

6,6'-((1,4,10,13-tetraoxa-7,16-diazacyclooctadecane-7,16-diyl)bis(methylene)) dipicolinamide[3] (K22_PicAm): 6,6'-((1,4,10,13-tetraoxa-7,16-diazacyclooctadecane-7,16-diyl) bis(methylene))dipicolinate (**1**) was added to an ice-cold solution of concentrated $NH_{3(aq)}$ (35 % wt., 25 mL) at 0 °C. The mixture was left to stir at room temperature overnight. The product was extracted from the aqueous layer with DCM (5 × 50 mL), the organic layers were combined, dried over $MgSO_4$, filtered, and concentrated under vacuum. The resulting crude residue was purified by reverse-phase chromatography (C18, 0-100 % MeCN + 0.3 % $NH_{3(aq)}$ in H_2O + 0.3 % $NH_{3(aq)}$) to give the product as an off-white solid (0.10 g, 0.19 mmol, 21 %). ^{1}H NMR (400 MHz, CD_3OD) δ_H (ppm): 7.94 (4H, m, Py-C*H*), 7.65 (2H, dd, $^{3}J_{HH}$ = 7.4, $^{4}J_{HH}$ = 1.5 Hz, Py-C*H*), 3.98 (4H, s, C*H*$_2$Py), 3.68 (8H, t, $^{3}J_{HH}$ = 5.3 Hz, macro-*H*), 3.58 (8H, s, macro-*H*), 2.90 (8H, t, $^{3}J_{HH}$ = 5.2 Hz, macro-*H*). $^{13}C\{^{1}H\}$ NMR (101 MHz, CD_3OD) δ_C (ppm): 169.3, 159.5, 150.5, 139.2, 127.7, 121.8, 71.5, 69.9, 60.8, 55.5. HRMS (ES-TOF+): *m/z* calcd. for $C_{26}H_{39}N_6O_6$ ($[M+H]^+$) 531.2931. found: 531.2938.

Dimethyl6,6'-((18-nitro-2,3,5,6,8,9,11,12,14,15-decahydro-4*H*,13*H*benzo[*b*][1,4,10,13] tetraoxa [7,16]diazacyclooctadecine-4,13-diyl)bis(methylene)) dipicolinate (3): 18-nitro-3,4,5,6,8,9,12,13,14,15-decahydro-2*H*,11*H*-benzo[*b*][1,4,10,13]tetraoxa[7,16]diaza cyclooctadecine (**2**) (0.75 g, 2.11 mmol), 6-chloromethylpyridine-2-carboxylic acid methyl ester (0.82 g, 4.43 mmol) and Na_2CO_3 (0.89 g, 8.44 mmol) were added to dry MeCN (30 mL) under N_2. The reaction mixture was stirred at 80 °C under N_2 for 3 days. The mixture was then cooled to room temperature, filtered and the solvent was removed under vacuum. The resulting crude residue was purified by flash silica column chromatography (SiO_2, DCM:MeOH, 85:15) to give the pure product as an orange oil (0.22 g, 0.34 mmol, 16 %). ^{1}H NMR (400 MHz, $CDCl_3$) δ_H (ppm): 7.90 (2H, m, Py-C*H*), 7.78 (3H, m, Py-C*H*/Ar-C*H*), 7.69 (2H, d, *J* = 7.8 Hz, Py-C*H*), 7.56 (1H, d, *J* = 2.7 Hz, Ar-C*H*), 6.77 (2H, d, *J* = 8.9 Hz, Ar-C*H*), 4.22 (4H, dt, *J* = 13.8, 5.3 Hz, macro-*H*), 4.02 (4H, s, CH_2Py), 3.95 (6H, d, *J* = 1.9 Hz, PyCOOCH_3), 3.70 (4H, dt, *J* = 6.6, 3.3 Hz, macro-*H*), 3.58 (4H, s, macro-*H*), 3.18 (4H, q, *J* = 5.3 Hz, macro-*H*), 2.97 (4H, q, *J* = 5.3 Hz, macro-*H*). ^{13}C{^{1}H} NMR (101 MHz, $CDCl_3$) δ_C (ppm): 165.9, 160.0, 153.5, 147.7, 147.1, 146.9, 141.3, 137.9, 126.8, 126.7, 123.8, 123.8, 118.0, 110.9, 107.4, 70.2, 68.9, 60.2, 54.7, 54.5, 53.1. HRMS (ES-TOF+): *m/z* calcd. for $C_{32}H_{40}N_5O_{10}$ ($[M+H]^+$) 654.2775. found: 654.2786.

6,6'-((18-nitro-2,3,5,6,8,9,11,12,14,15-decahydro-4*H*,13*H*-benzo[*b*][1,4,10,13]tetraoxa[7,16] diazacyclooctadecine-4,13-diyl)bis(methylene))dipicolinamide (NPK_PicAm): Dimethyl 6,6'-((18-nitro-2,3,5,6,8,9,11,12,14,15-decahydro-4*H*,13*H*-benzo[*b*][1,4,10,13]tetraoxa[7,16] diazacyclooctadecine-4,13-diyl)bis(methylene))dipicolinate (**3**) (0.86 g, 1.32 mmol) was added to $NH_{3(aq)}$ (35 mL) and stirred at room temperature for 5 days. The mixture was concentrated under vacuum and the crude residue was purified by reverse-phase column chromatography (C18, 0-100 % MeCN + 0.3 % $NH_{3(aq)}$ in H_2O + 0.3 % $NH_{3(aq)}$) to give the product as a yellow solid (0.31 g, 0.50 mmol, 38 %). ^{1}H NMR (400 MHz, CD_3OD) δ_H (ppm): 7.93 (2H, dt, *J* = 7.7, 1.6 Hz, Py-C*H*), 7.84 (3H, m, Py-C*H*/Ar-C*H*), 7.72 (2H, dt, *J* = 7.7, 1.6 Hz, Py-C*H*), 7.68 (1H, d, *J* = 2.7 Hz, Ar-C*H*), 6.97 (1H, d, *J* = 9.0 Hz, Ar-C*H*), 4.23 (4H, dt, *J* = 10.5, 5.5 Hz, macro-*H*), 4.03 (4H, d, *J* = 3.2 Hz, Py-CH_2), 3.73 (4H, t, *J* = 5.6 Hz, macro-*H*), 3.60 (4H, s, macro-*H*), 3.35 (4H, s, macro-*H*), 3.22 (4H, t, *J* = 5.5 Hz, macro-*H*), 3.04 (4H, dt, *J* = 5.9, 2.9 Hz, macro-*H*). ^{13}C{^{1}H} NMR (101 MHz, CD_3OD) δ_C (ppm): 169.4, 160.3, 155.5, 150.4, 149.6, 142.5, 139.1, 127.5, 121.7, 118.7, 112.2, 108.5, 71.6, 70.5, 68.9, 68.8, 61.4, 55.7, 55.7, 54.4, 54.3. HRMS (ES-TOF+): *m/z* calcd. for $C_{30}H_{38}N_7O_8$ ($[M+H]^+$) 624.2782. found: 624.2785.

3. Pb^{2+} Complex Formation

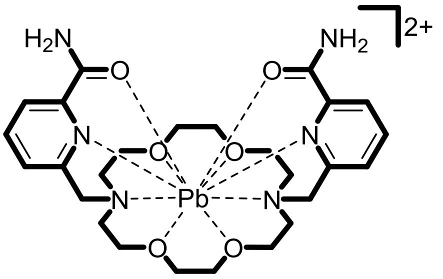

[Pb(K22_PicAm)][HCOOH]$_2$: K22_PicAm (0.03 g, 0.06 mmol) was dissolved in 20 mM NH_4OAc (pH 4.5, 5 mL). $Pb(OAc)_2.3H_2O$ (0.027 g, 0.07 mmol) was added to the solution and stirred at room temperature for 15 minutes. The reaction mixture purified by reverse-phase chromatography (0-100 % MeCN + 0.1 % FA in H_2O + 0.1 % FA) to give the product as a yellow oil (0.03 g, 0.04 mmol, 60 %). ^{1}H NMR (400 MHz, CD_3OD) δ_H (ppm): 8.06 (2H, t, J = 7.8 Hz, Py-C*H*), 7.76 (4H, dd, J = 7.8, 5.2 Hz, Py-C*H*), 5.14 (2H, d, J = 15.8 Hz, CH_2Py), 4.45 (2H, t, J = 11.0 Hz, macro-*H*), 3.98 (2H, t, J = 11.0 Hz, macro-*H*), 3.81 (6H, m, CH_2Py/macro-*H*), 3.62 (4H, dd, J = 13.2, 9.9 Hz, macro-*H*), 3.52 (2H, d, J = 9.5 Hz, macro-*H*), 3.41 (4H, dd, J = 21.4, 7.6 Hz, macro-*H*), 2.59 (4H, m, macro-*H*), 2.41 (2H, d, J = 13.8 Hz, macro-*H*). $^{13}C\{^1H\}$ NMR (101 MHz, CD_3OD) δ_C (ppm): 170.4, 167.5, 162.2, 148.5, 141.1, 129.3, 123.0, 71.7, 70.9, 69.6, 68.2, 60.7, 56.7, 55.2. $^{207}Pb\{^1H\}$ NMR (84 MHz, D_2O) δ_{Pb} (ppm): -2289. HRMS (ES-TOF+): *m/z* calcd. for $C_{26}H_{37}N_6O_6Pb$ ([M-HCOOH]$^+$) 737.2541. found: 737.2521. ([M-2(HCOOH)]$^{2+}$) found: 369.1251.

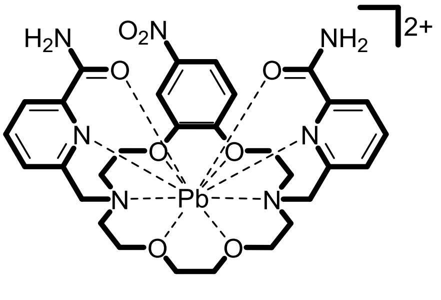

[Pb(NPK_PicAm)][HCOOH]$_2$: NPK_PicAm (0.05 g, 0.08 mmol) was dissolved in CD_3OD (10 mL). $Pb(OAc)_2.3H_2O$ (0.03 g, 0.08 mmol) was added to the solution and stirred at room temperature for a total of 2 hours (aliquots taken for ^{1}H NMR and HPLC analysis at t = 0, 0.5, 1 and 2 hours, **Fig. S20** and **Fig. S30**). The mixture was then heated at 70 °C for a total of 128 hours (aliquots taken for ^{1}H NMR and HPLC analysis at t = 0.5, 1, 2, 24, 40 and 128 hours, **Fig. S20** and **Fig. S30**). HRMS (ES-TOF+): *m/z* calcd. for $C_{30}H_{36}N_7O_8Pb$ ([M-HCOOH]$^+$) 830.2386. found: 830.2378. ([M-2(HCOOH)]$^{2+}$) calcd. 415.6231. found: 415.6230.

4. Radiochemistry

Radiolabelling procedures:

Caution: ^{212}Pb emits ionizing radiation and should only be used in a facility in accordance with appropriate safety controls.

Radiolabelling experiments were conducted for **K22_PicAm** and **NPK_PicAm** with [^{212}Pb]Pb(OAc)$_2$. **For K22_PicAm, the radiolabelling reactions were followed at two temperatures (37 °C and 95 °C) and a single time point of 15 minutes. For NPK_PicAm, the radiolabelling reactions were performed at 95 °C for 15 minutes.** Stock solutions, with a concentration of 0.02

M of **K22_PicAm** and **NPK_PicAm** were prepared in NaOAc (0.5 M, pH 5). Concentration-dependent experiments with both **K22_PicAm** and **NPK_PicAm** were conducted by serial dilution of stock solutions to prepare chelator solutions with concentrations between 10^{-3} and 5×10^{-6} M **(1 mM, 100 µM, 50 µM, 10 µM and 5 µM)**. For $[^{212}Pb]Pb^{2+}$ labelling experiments, a 5 µL aliquot of chelator (or 0.5 M NaOAc for the negative control) was added to 100 µL of $[^{212}Pb]Pb(OAc)_2$ (1 MBq, 0.5 M NaOAc, pH 5.5) and mixed to begin the reactions, which were left at either 37 °C or 95 °C for 15 minutes. iTLC was used to determine radiochemical conversion (RCC) of the reactions using iTLC-SG (iTLC paper impregnated with a silica gel) plates. The mobile phase used was a 0.1 M solution of NH_4OAc with 25 mM EDTA (pH 5.7). Using this iTLC system, $[^{212}Pb]Pb^{2+}$ labelled complex remained at the baseline (Rf = 0), while 'free' $[^{212}Pb]Pb^{2+}$ migrated at the solvent front (Rf = 1), shown below, Fig. S1-S3. Aliquots of the reactions were taken at 15 minutes, spotted onto the plates, and developed. RCCs were measured using a Perkin Elmer Cyclone phosphor imager. Radiochemical conversions (RCCs) were determined once the resulting iTLC-SG plates were left to decay for 18-24 hours to allow for the decay of daughter radionuclides (^{212}Bi and ^{208}Tl) before measurements were taken.

5. Radio-TLC Chromatograms

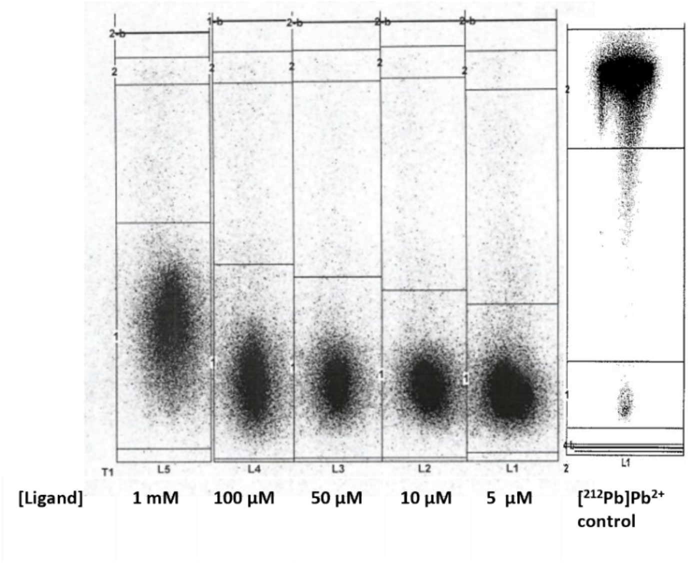

Figure S1. iTLC plates of $[^{212}Pb]Pb^{2+}$ labelling of **K22_PicAm** with 0.1 M NH_4OAc (pH 5.7) and 25 mM EDTA mobile phase. Reaction conditions: 95 °C, 15 minutes. A $[^{212}Pb]Pb^{2+}$ control was also performed using the same conditions, shown on the right.

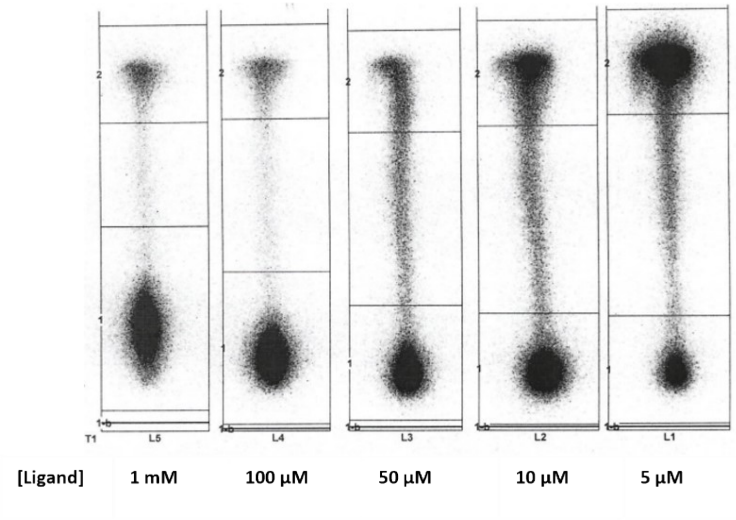

Figure S2. iTLC plates of [^{212}Pb]Pb^{2+} labelling of **K22_PicAm** with 0.1 M NH_4OAc (pH 5.7) and 25 mM EDTA mobile phase. Reaction conditions: 37 °C, 15 minutes.

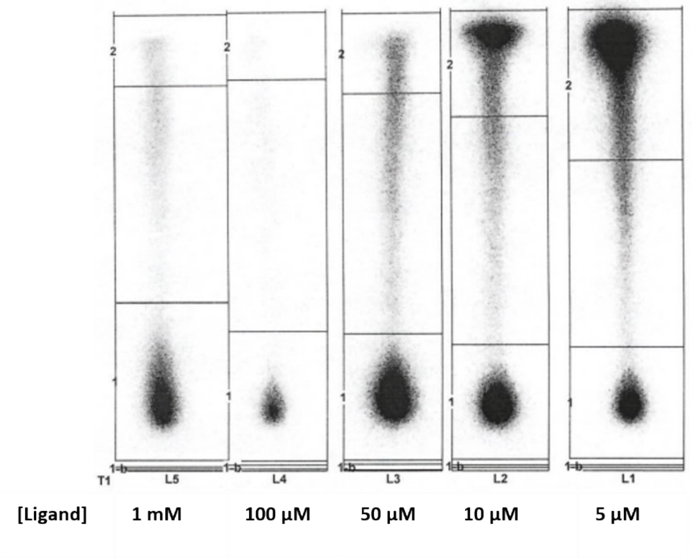

Figure S3. iTLC plates of [^{212}Pb]Pb^{2+} labelling of **NPK_PicAm** with 0.1 M NH_4OAc (pH 5.7) and 25 mM EDTA mobile phase. Reaction conditions: 95 °C, 15 minutes.

Table S1. The RCC values for [^{212}Pb]Pb^{2+} with **K22_PicAm** and **NPK_PicAm** at different ligand concentrations (1000 µM to 5 µM) in 0.5 M NaOAc (pH 5.5) over 15 min at 37 °C and 95 °C.

**Chelator**	**Concentration (µM)**	**Temperature (°C)**	**RCC (%)**
**K22_PicAm**	1000	95	99
		37	85
	100	95	99
		37	88
	50	95	99
		37	74

	10	95	99
		37	66
	5	95	99
		37	25
**NPK_PicAm**	1000	95	89
		37	-
	100	95	91
		37	-
	50	95	85
		37	-
	10	95	53
		37	-
	5	95	23
		37	-

6. NMR Spectroscopy

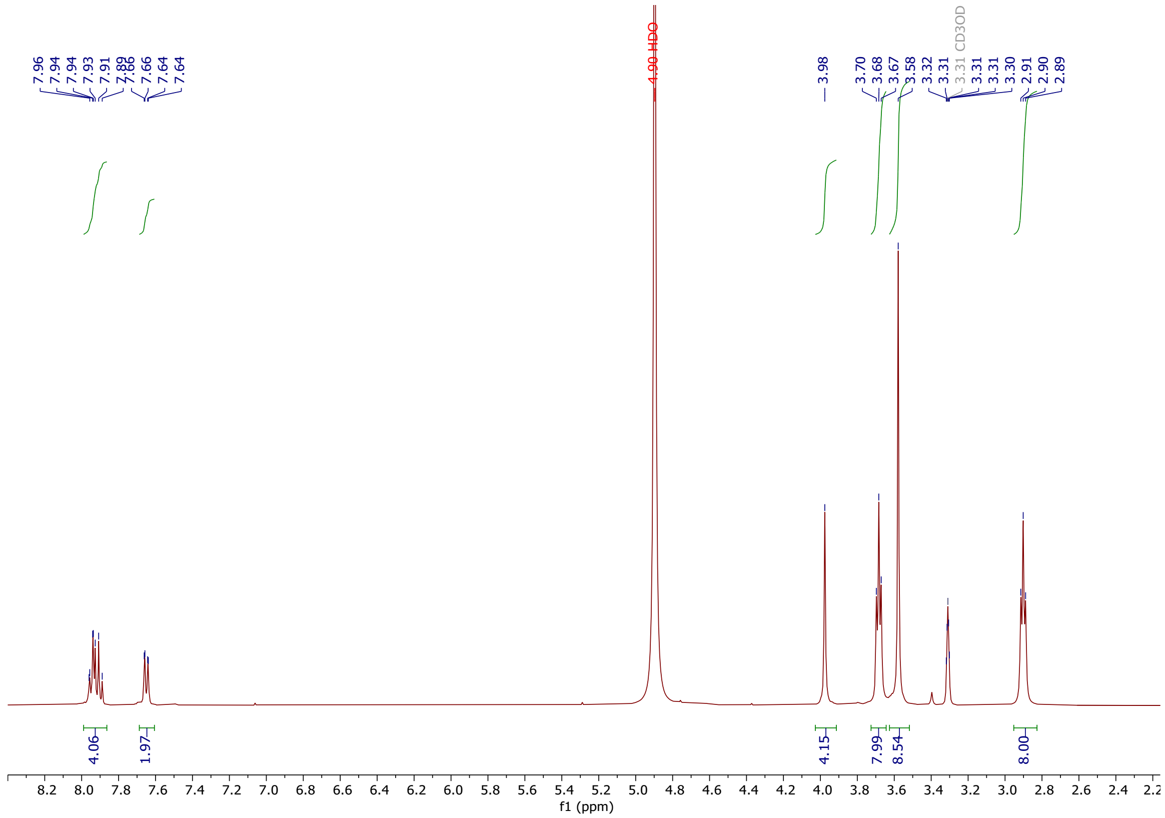

Figure S4. ^{1}H NMR spectrum of **K22_PicAm** (400 MHz, CD_3OD, 298 K).

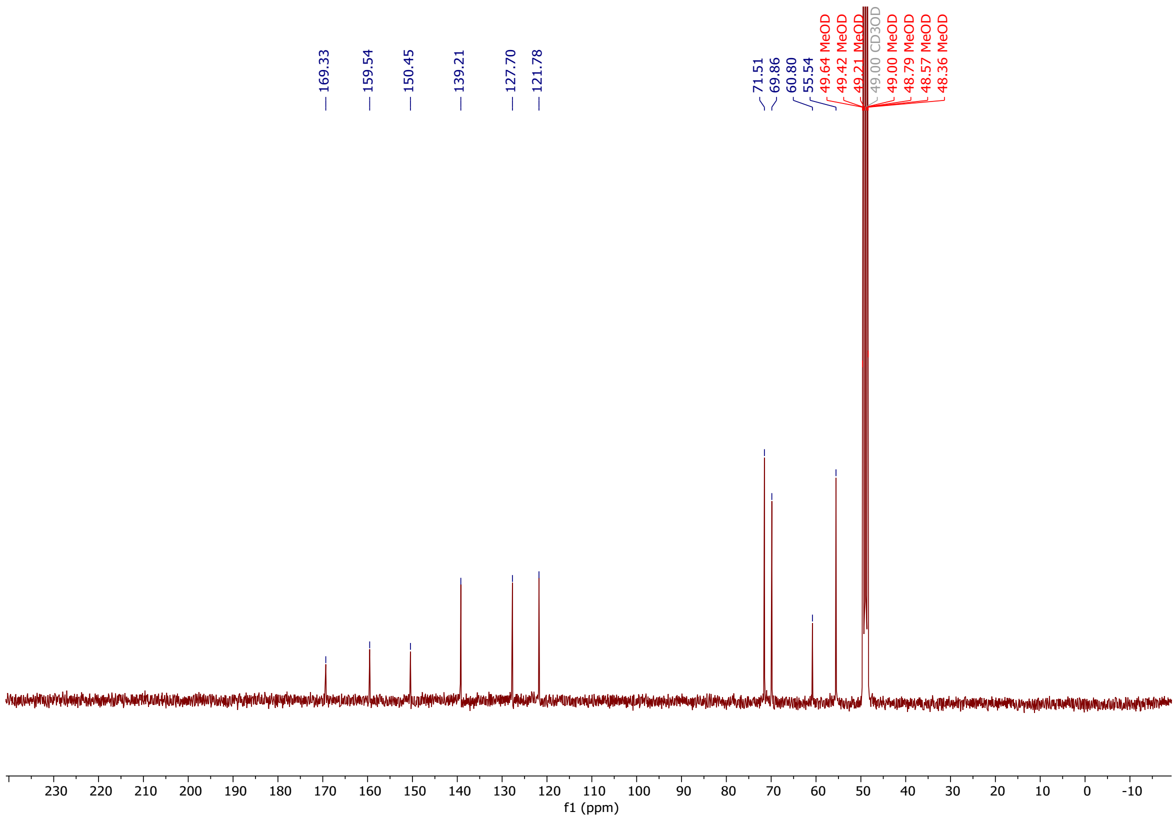

Figure S5. $^{13}C\{^{1}H\}$ NMR spectrum of **K22_PicAm** (101 MHz, CD_3OD, 298 K).

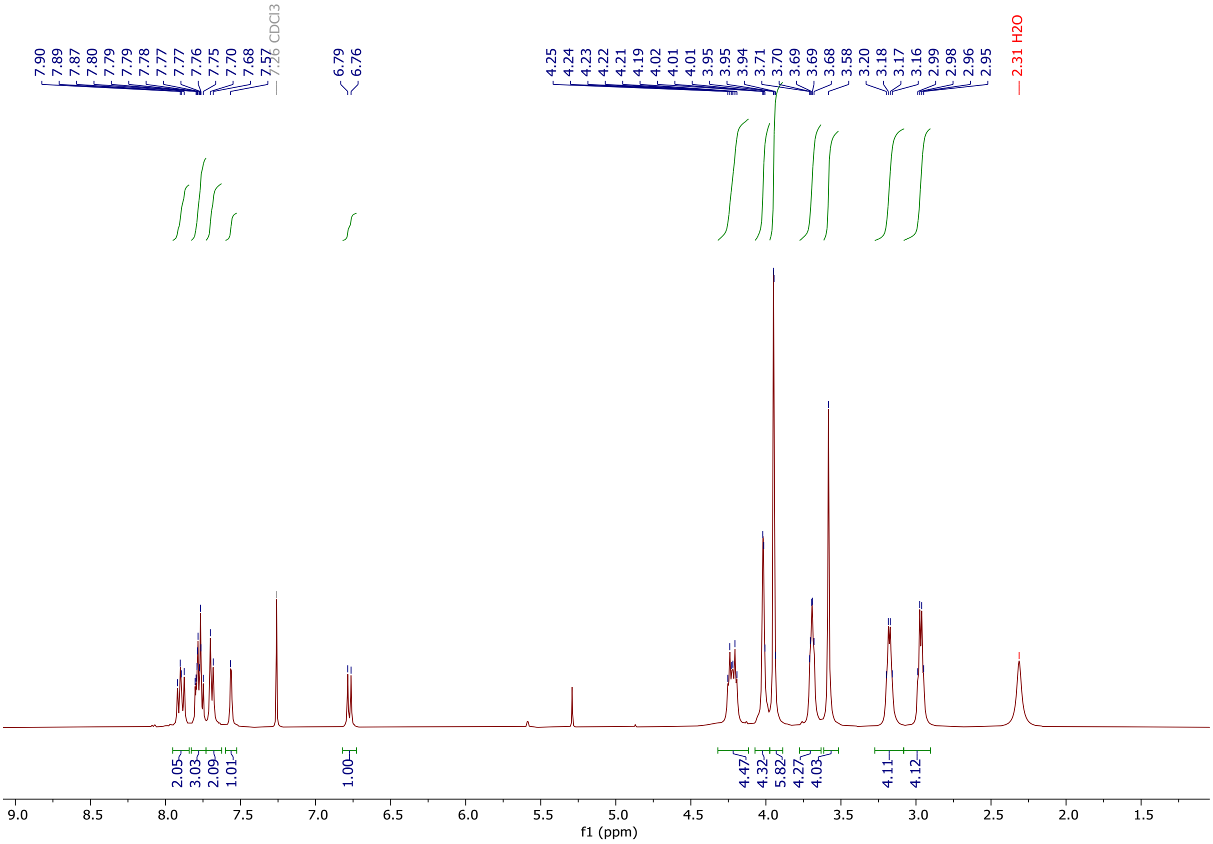

Figure S6. ^{1}H NMR spectrum of compound **3** (400 MHz, $CDCl_3$, 298 K).

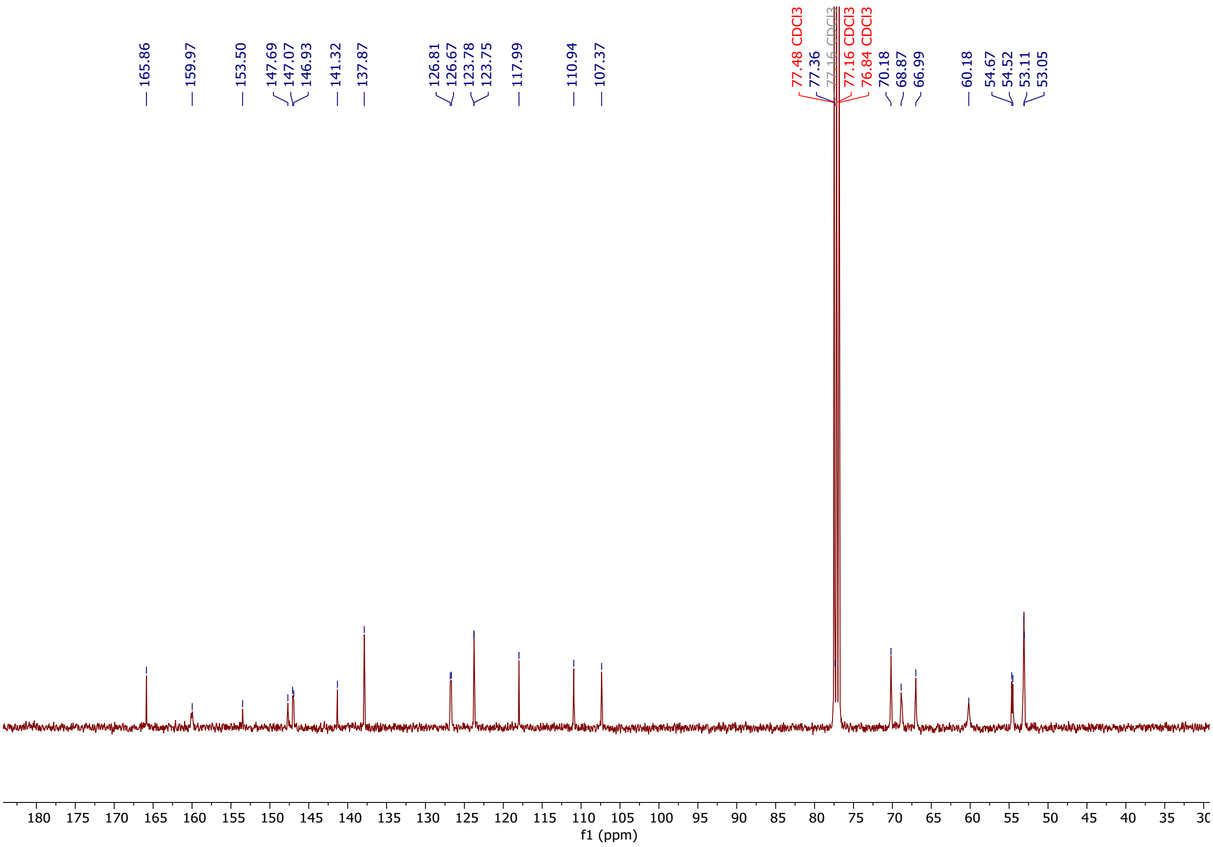

Figure S7. $^{13}C\{^{1}H\}$ NMR spectrum of compound **3** (101 MHz, $CDCl_3$, 298 K).

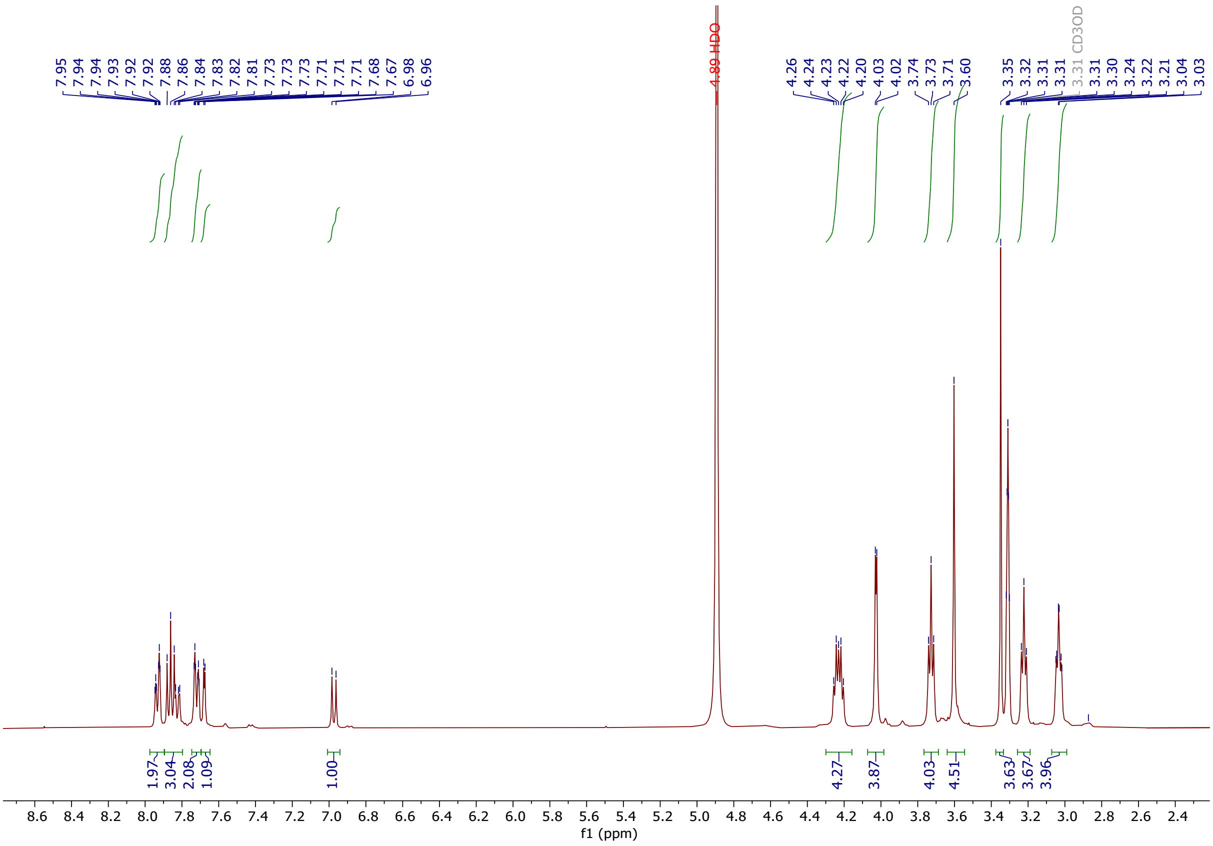

Figure S8. ^{1}H NMR spectrum of **NPK_PicAm** (400 MHz, CD_3OD, 298 K).

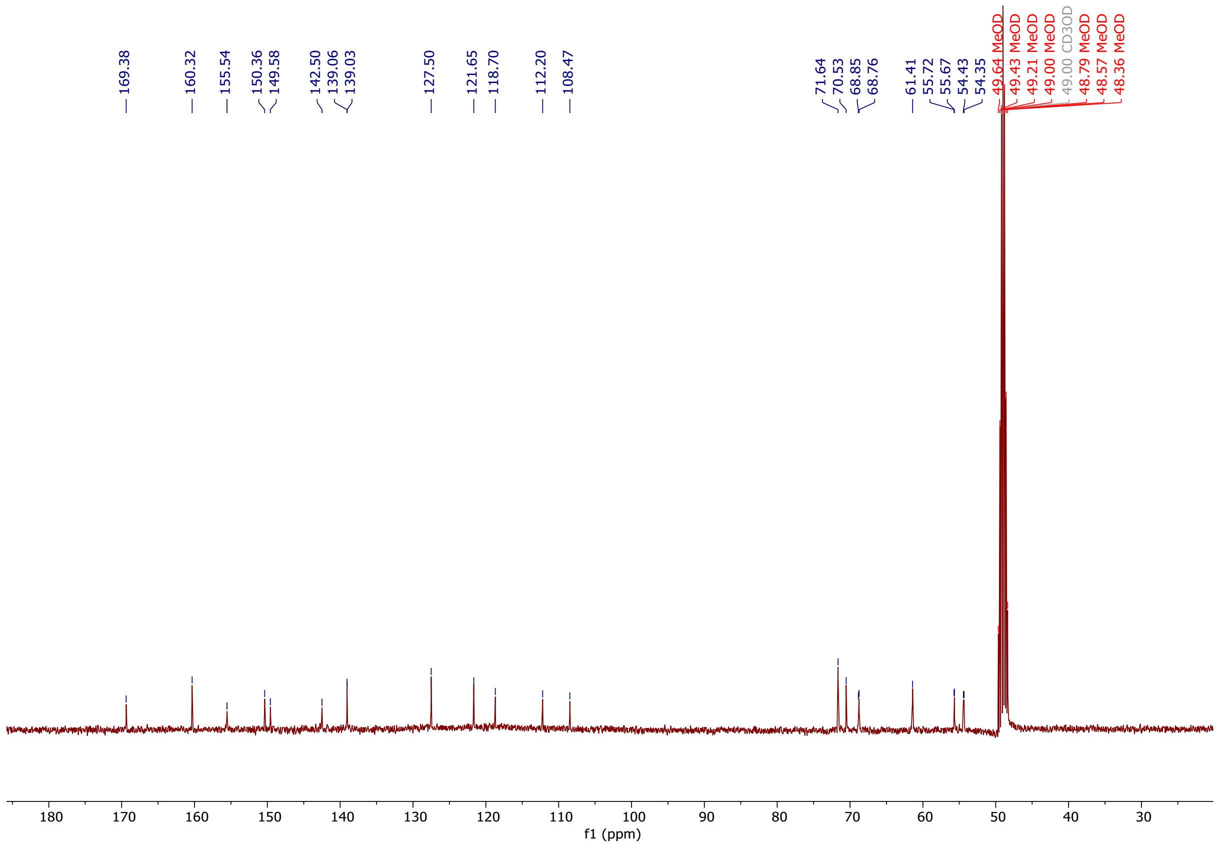

Figure S9. $^{13}C\{^{1}H\}$ NMR spectrum of **NPK_PicAm** (101 MHz, CD_3OD, 298 K).

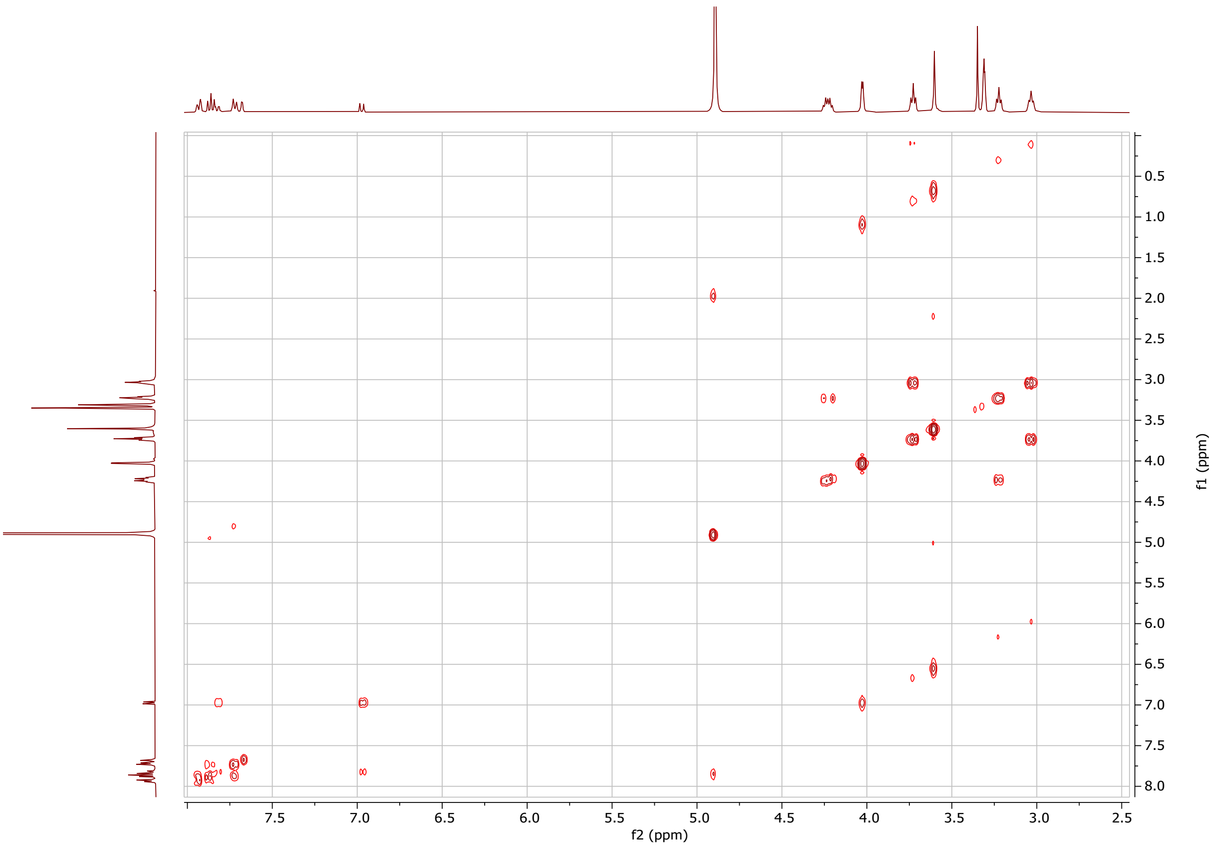

Figure S10. ${}^{1}H$-${}^{1}H$ COSY NMR spectrum of **NPK_PicAm** (400 MHz, CD_3OD, 298 K).

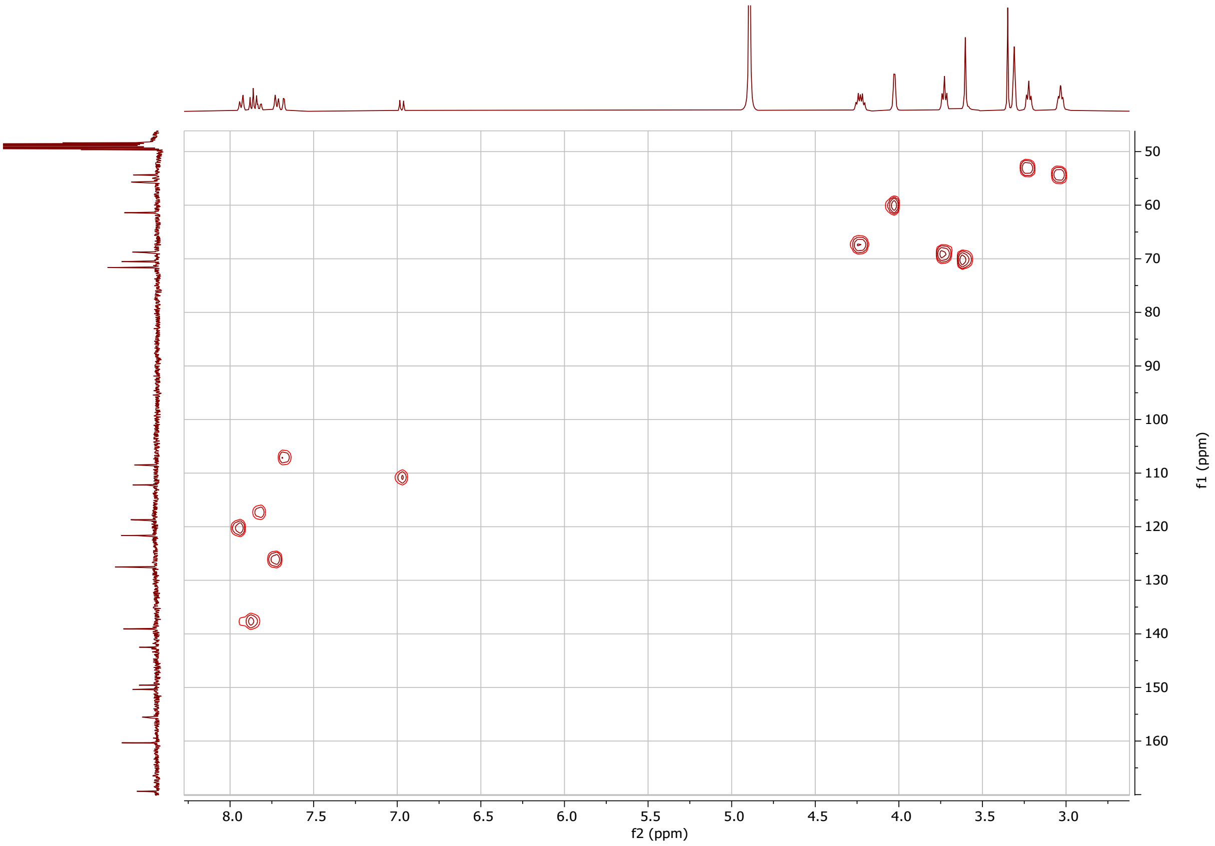

Figure S11. ^{1}H-^{13}C HSQC NMR spectrum of **NPK_PicAm** (CD_3OD, 298 K).

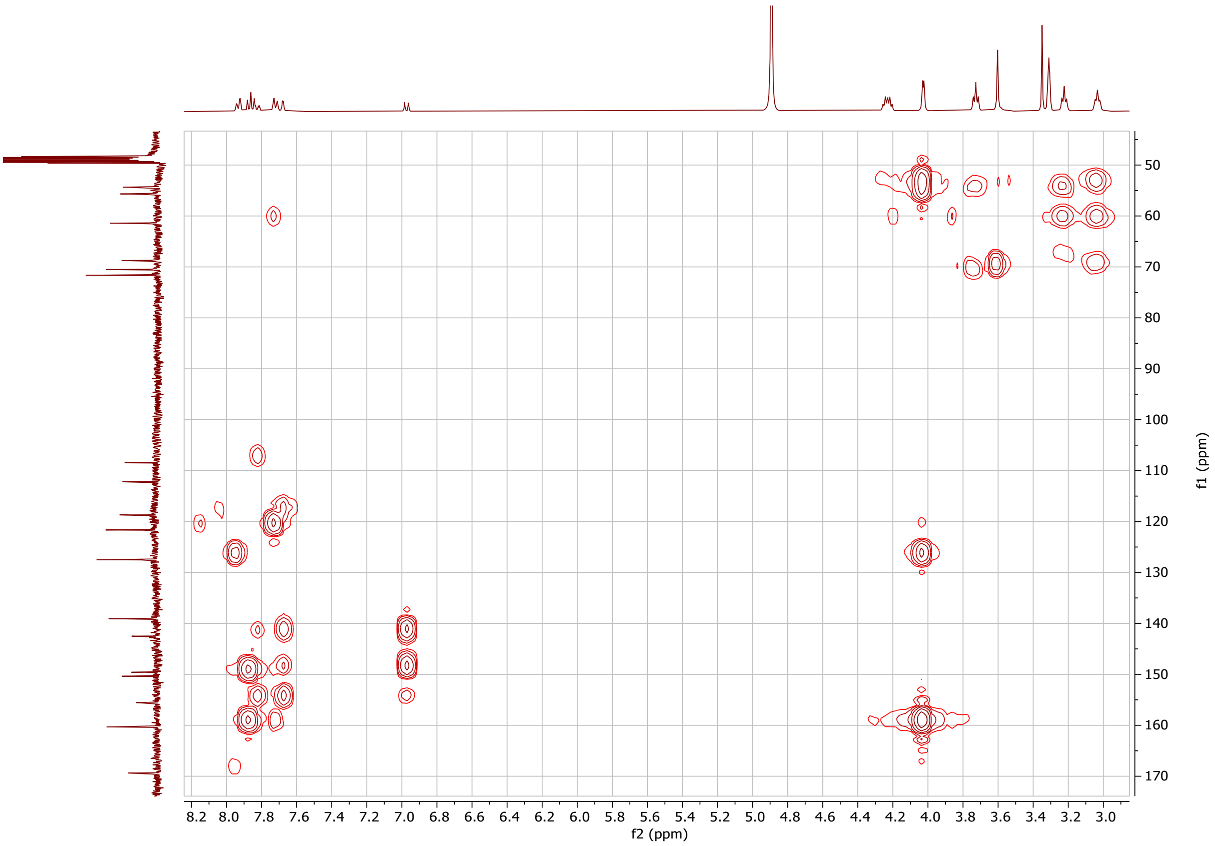

Figure S12. ^{1}H-^{13}C HMBC NMR spectrum of **NPK_PicAm** (CD_3OD, 298 K).

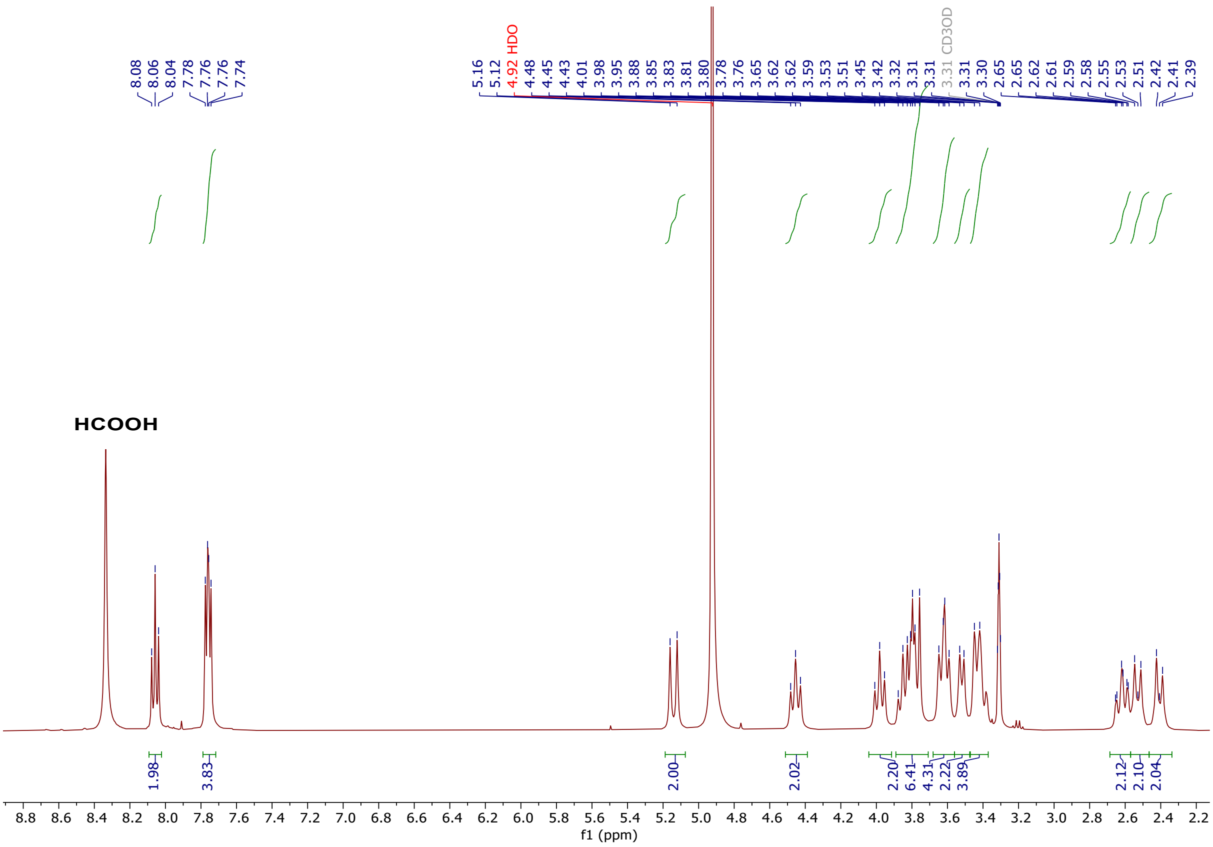

Figure S13. ^{1}H NMR spectrum of **[Pb(K22_PicAm)]$^{2+}$** (400 MHz, CD_3OD, 298 K).

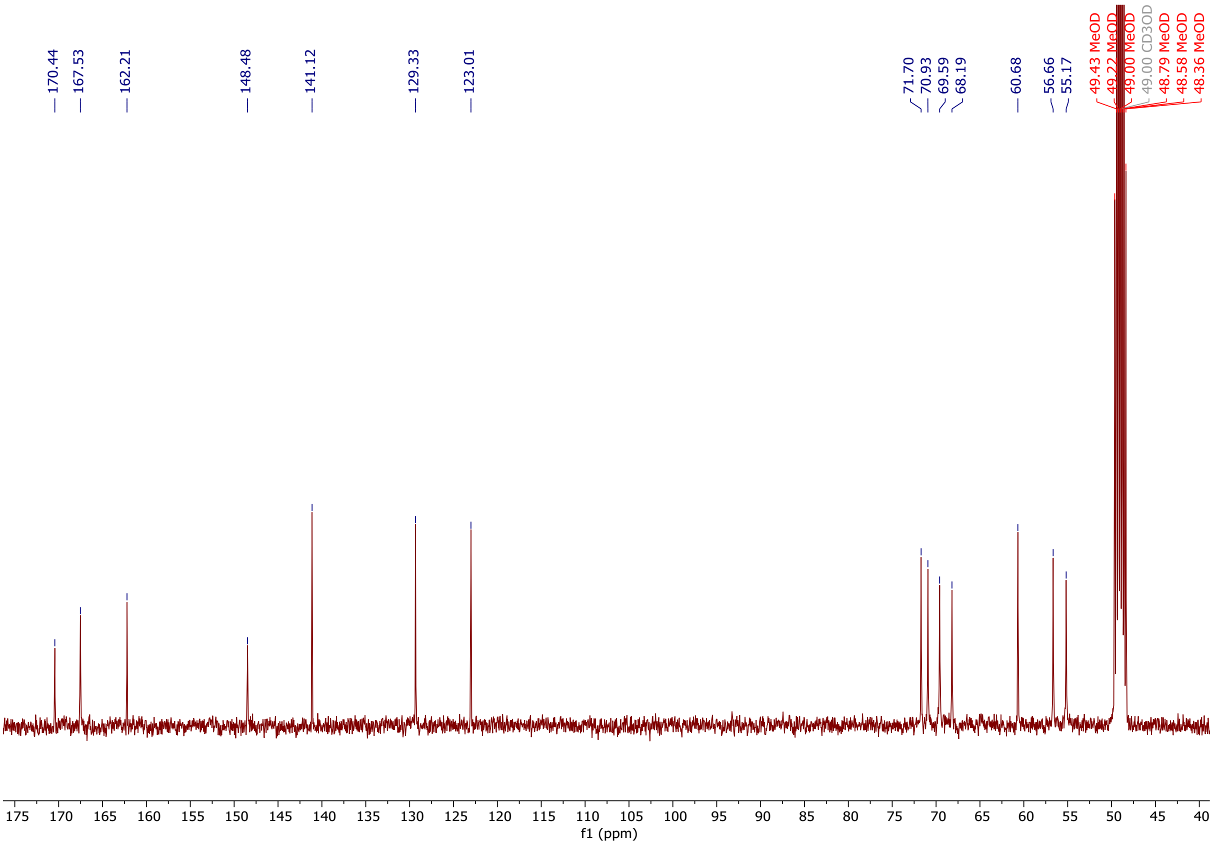

Figure S14. $^{13}C\{^{1}H\}$ NMR spectrum of **[Pb(K22_PicAm)]$^{2+}$** (101 MHz, CD_3OD, 298 K).

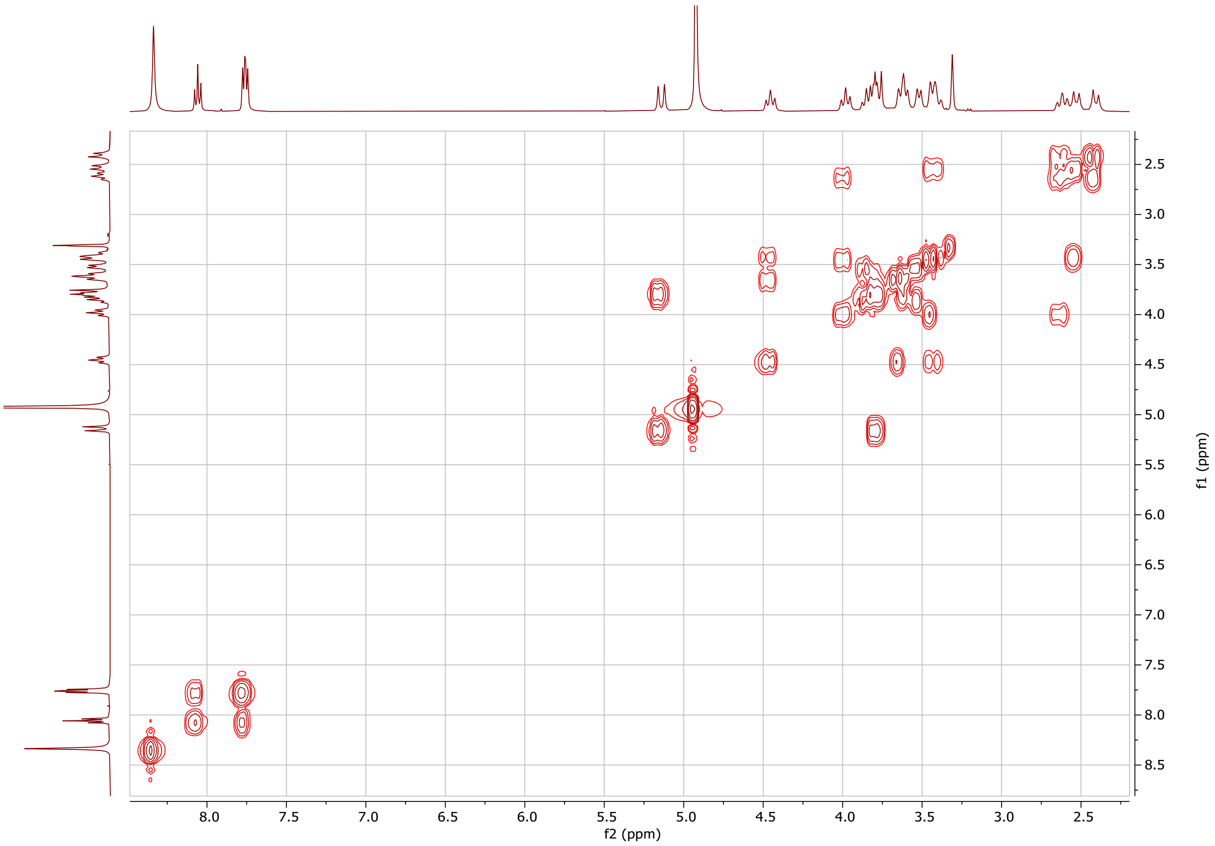

Figure S15. ^{1}H-^{1}H COSY NMR spectrum of **[Pb(K22_PicAm)]$^{2+}$** (400 MHz, CD_3OD, 298 K).

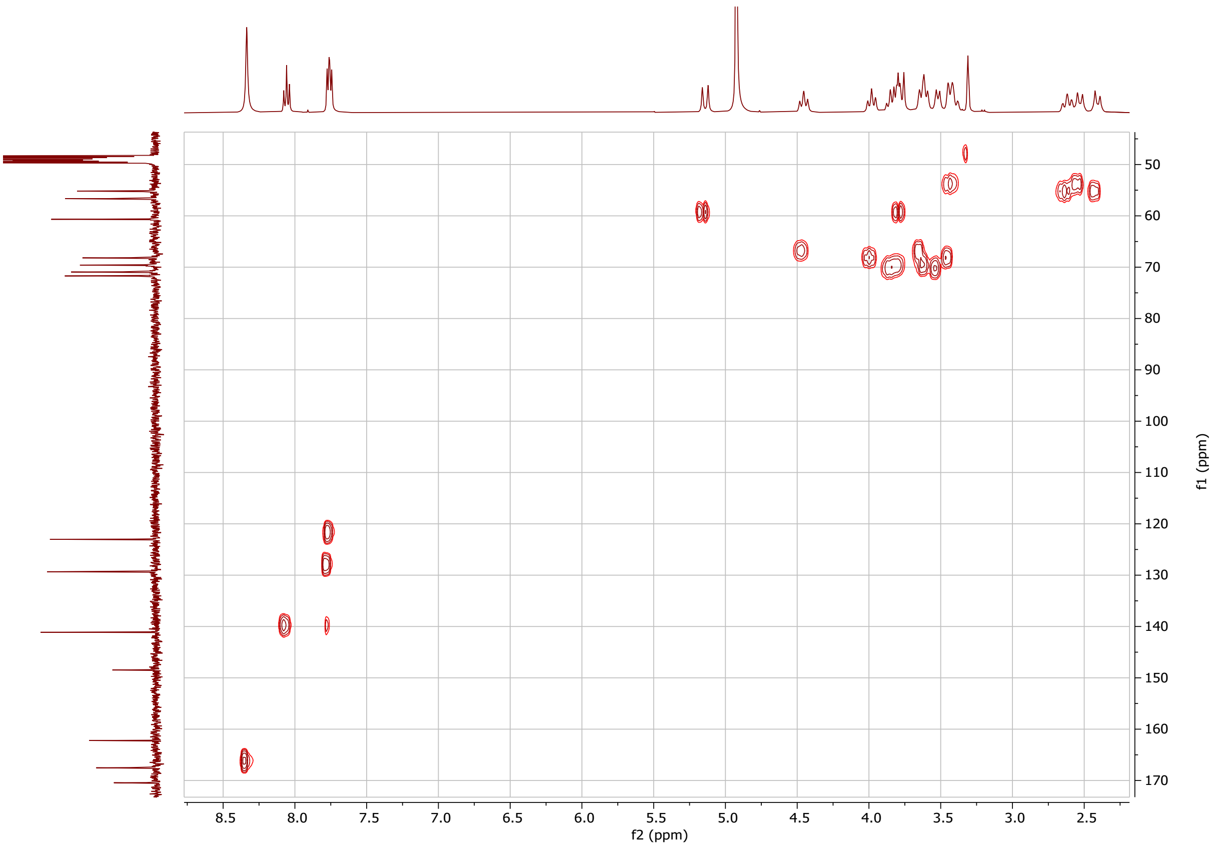

Figure S16. ^{1}H-^{13}C HSQC NMR spectrum of **[Pb(K22_PicAm)]$^{2+}$** (CD_3OD, 298 K).

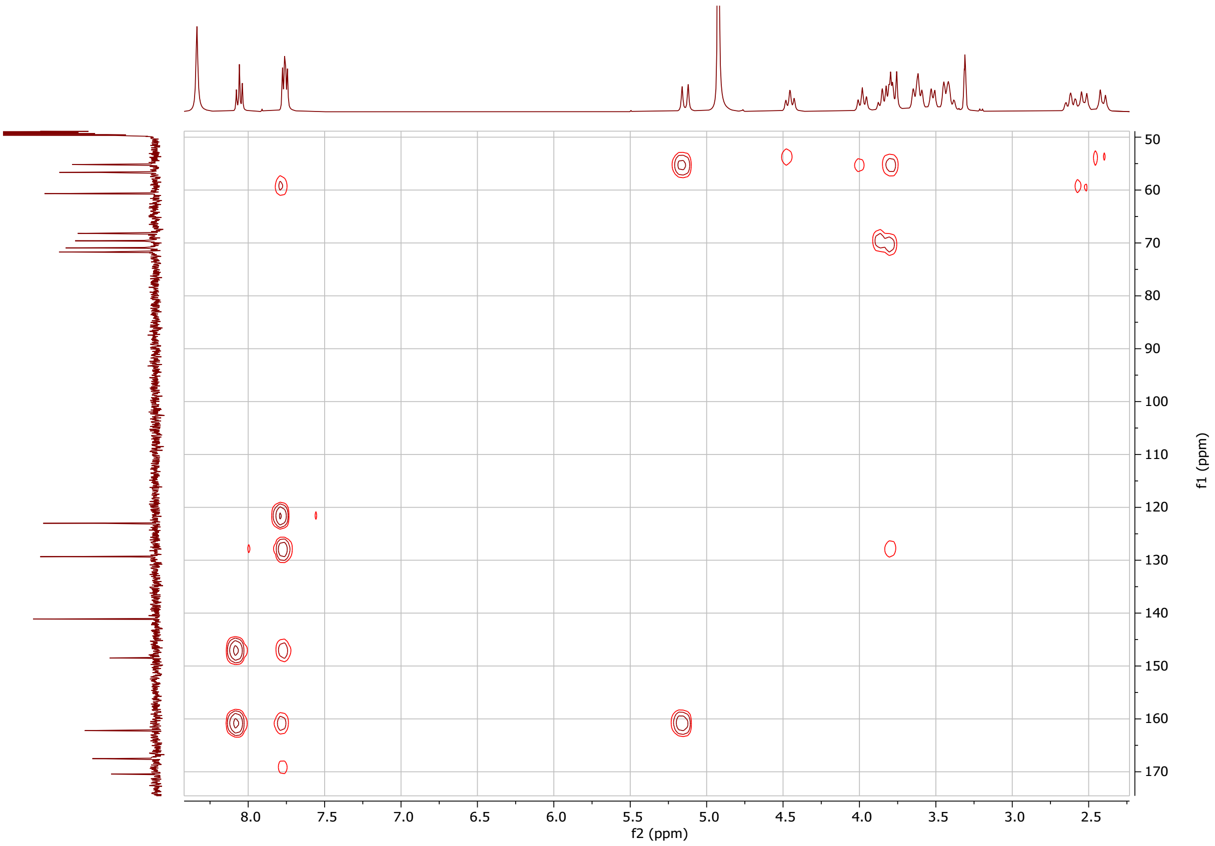

Figure S17. ^{1}H-^{13}C HMBC NMR spectrum of **$[Pb(K22\_PicAm)]^{2+}$** (CD_3OD, 298 K).

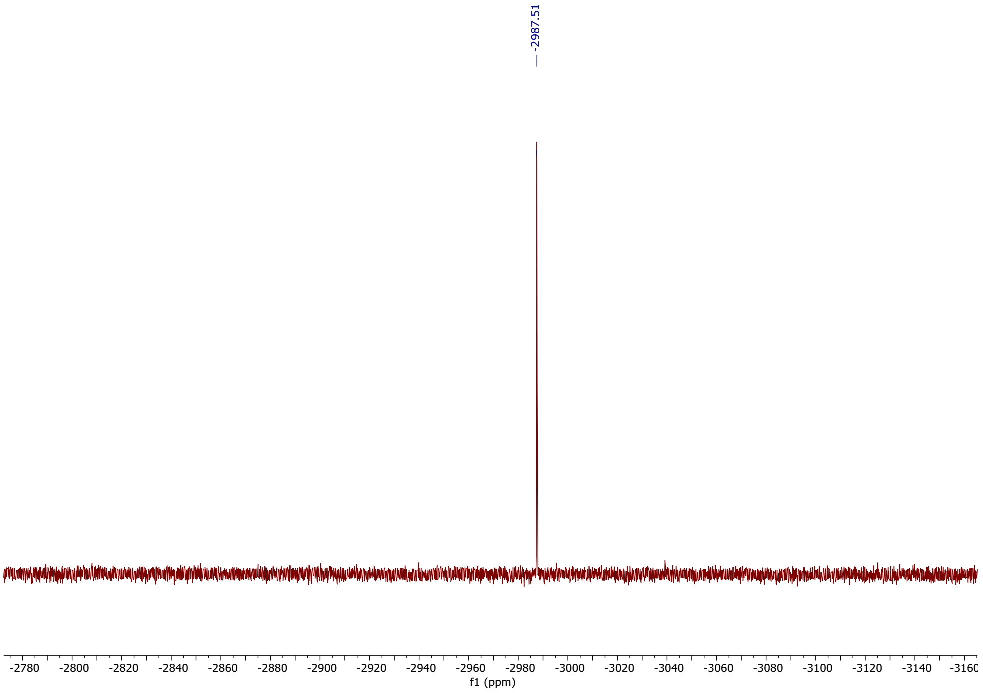

Figure S18. $^{207}Pb\{^{1}H\}$ NMR spectrum of 1 M $Pb(NO_3)_2$ (83 MHz, D_2O, 298 K).

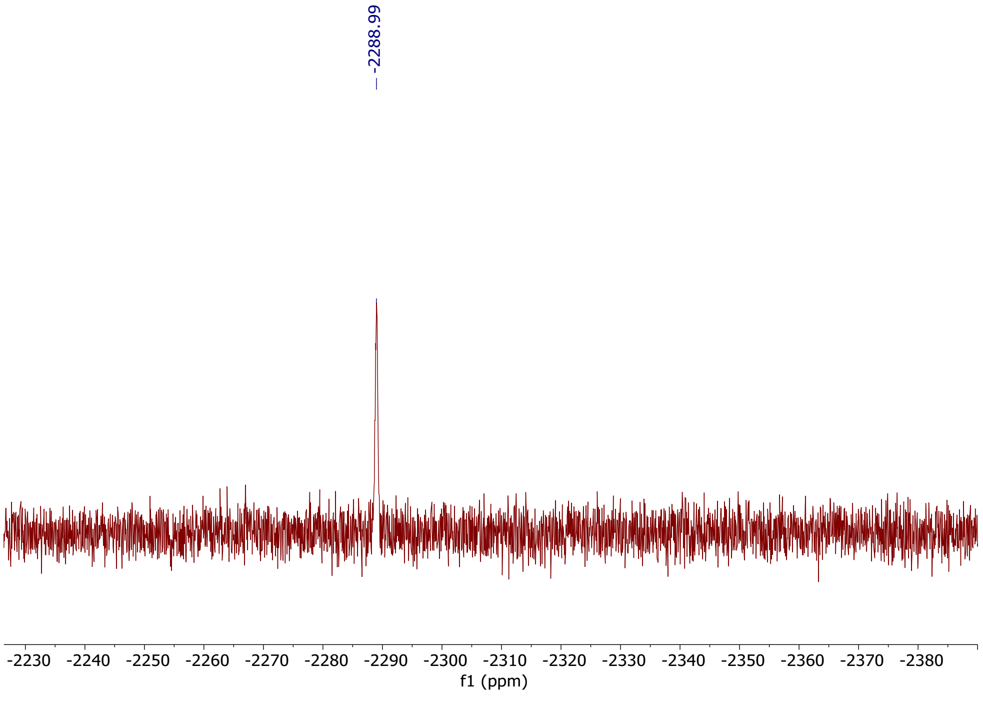

Figure S19. $^{207}Pb\{^{1}H\}$ NMR spectrum of **$[Pb(K22\_PicAm)]^{2+}$** (84 MHz, D_2O, 298 K).

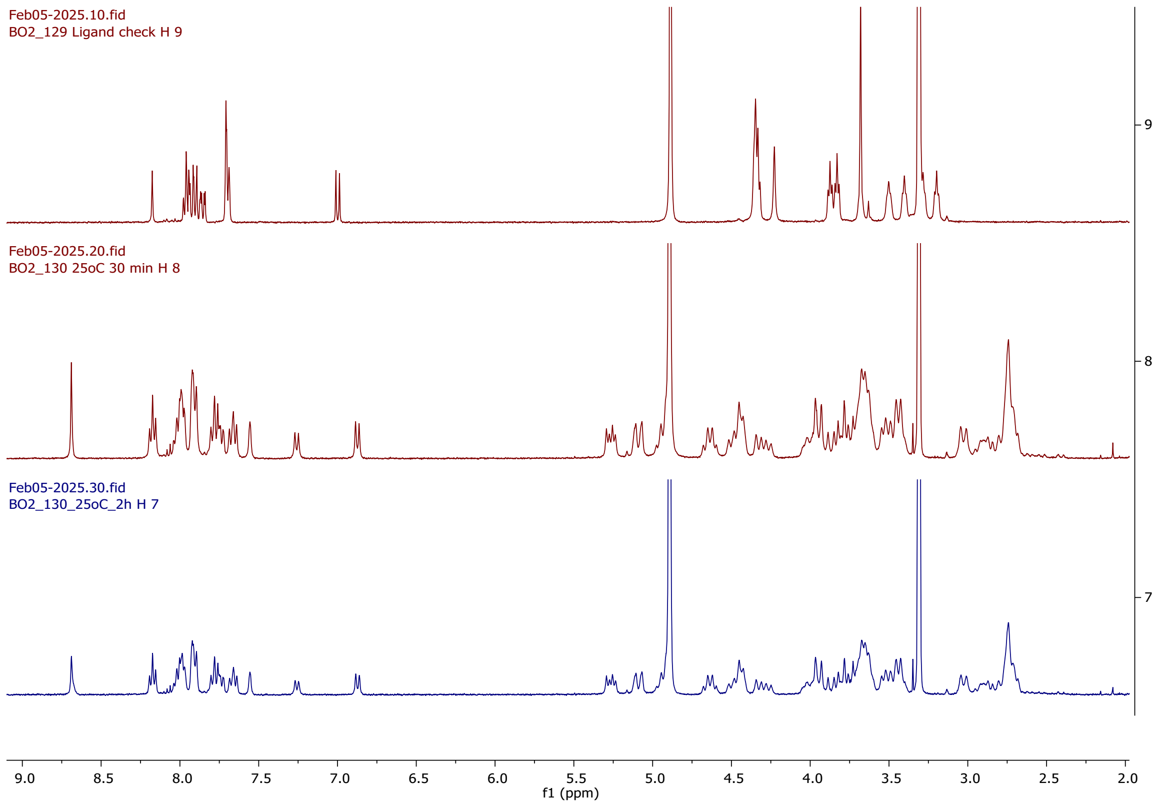

Figure S20. ^{1}H NMR spectrum of $Pb(OAc)_2.3H_2O$ complexation with **NPK_PicAm** (400 MHz, CD_3OD, 298 K).

7. Mass Spectra

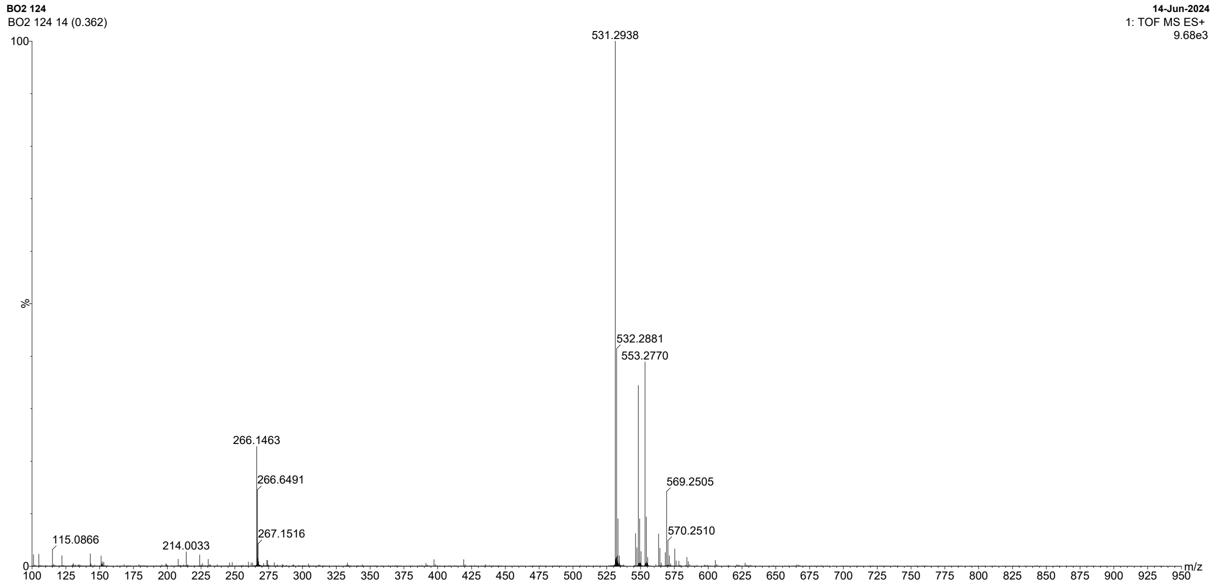

Figure S21. HRMS (ES-TOF+) of **K22_PicAm**.

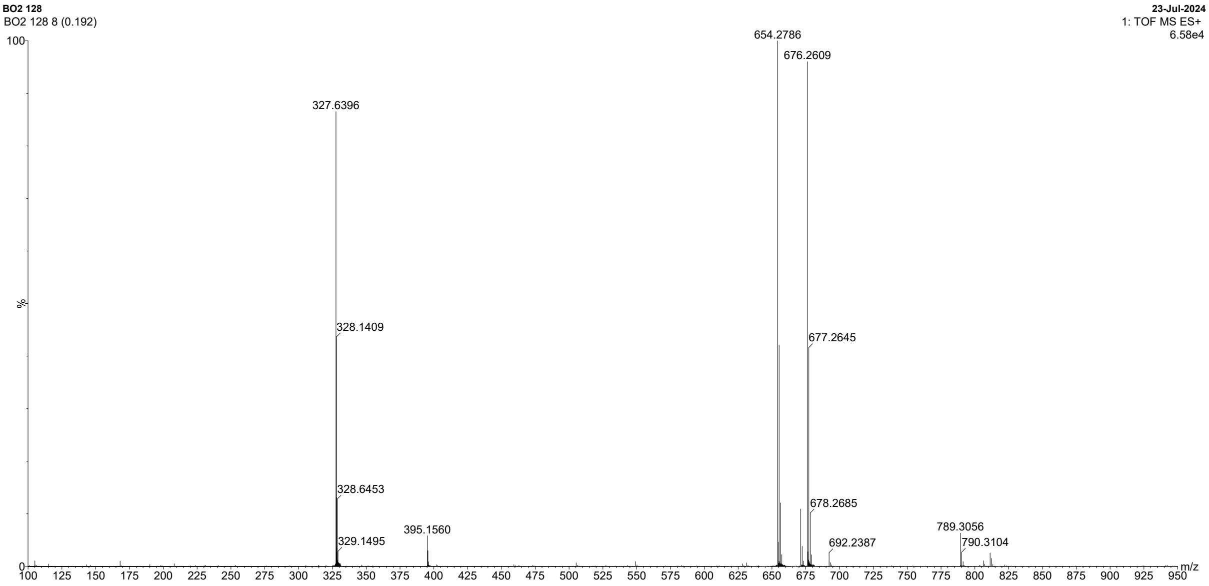

Figure S22. HRMS (ES-TOF+) of compound **3**.

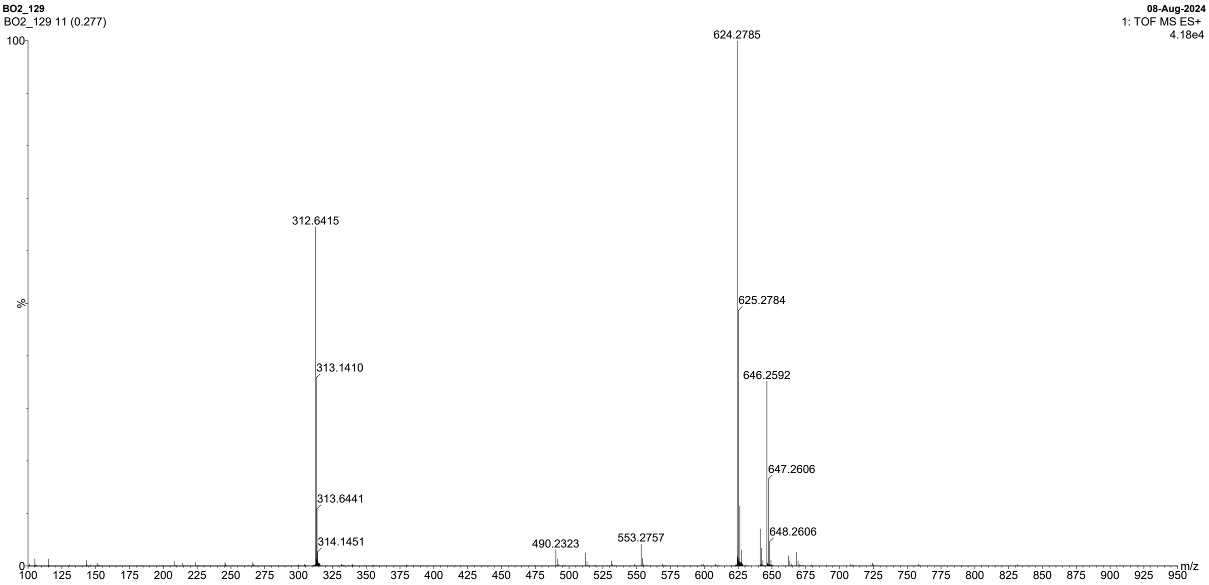

Figure S23. HRMS (ES-TOF+) of **NPK_PicAm**.

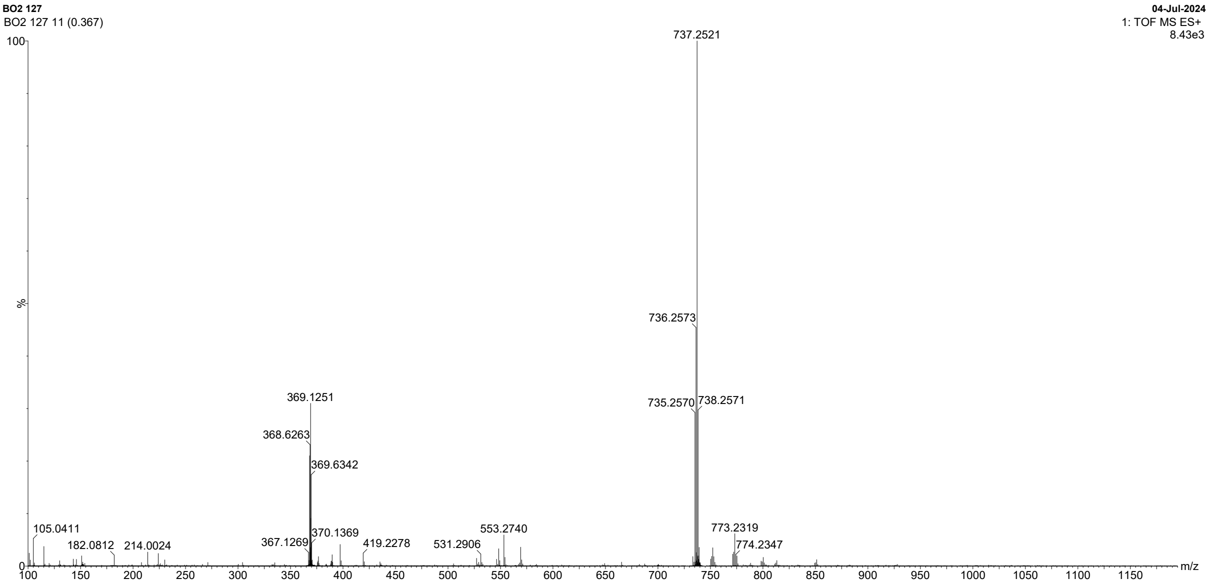

Figure S24. HRMS (ES-TOF+) of **[Pb(K22_PicAm)]$^{2+}$**.

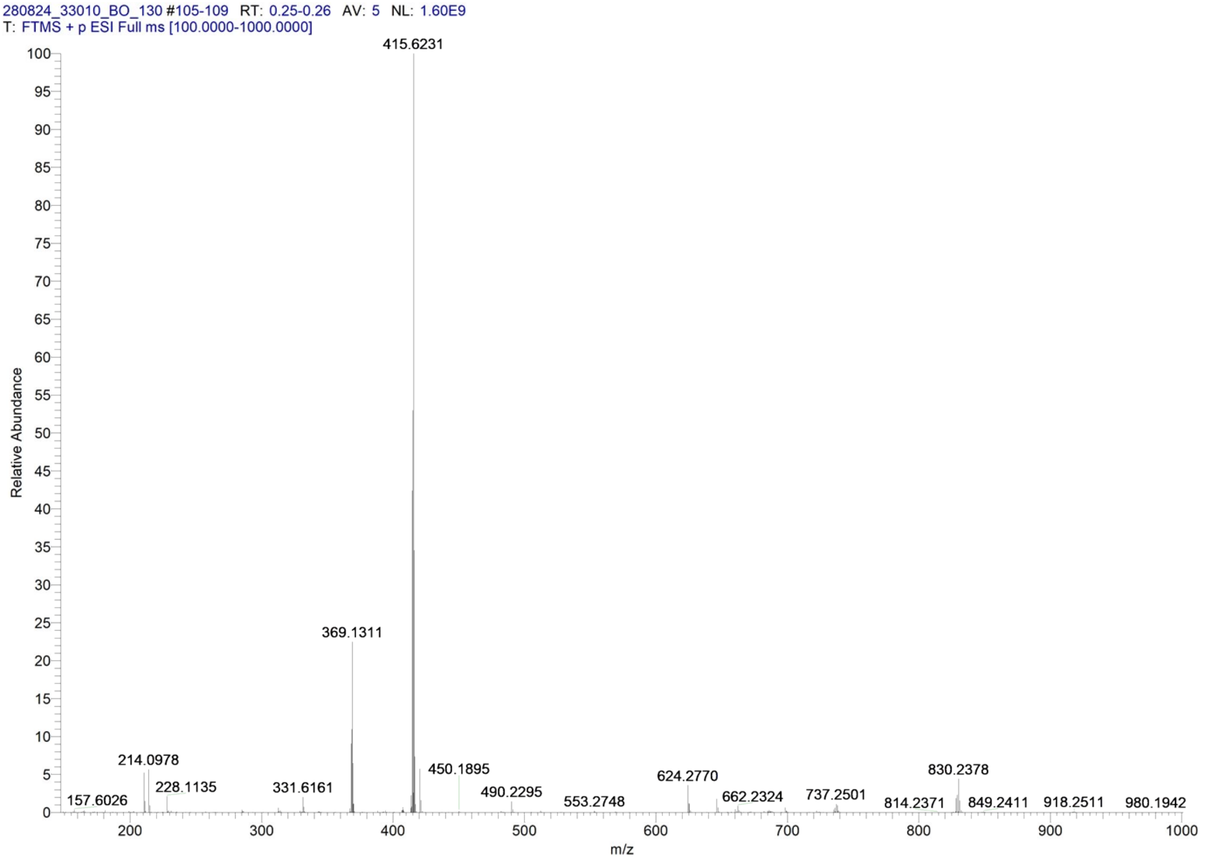

Figure S25. HRMS (ES-TOF+) of **[Pb(NPK_PicAm)]$^{2+}$**.

8. HPLC Spectra

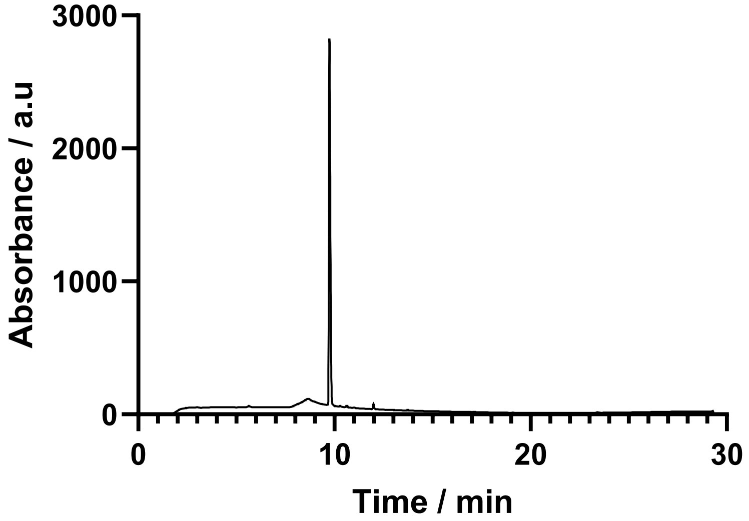

Figure S26. HPLC UV trace of **K22_PicAm**. HPLC was performed on an Agilent 1260 Infinity II HPLC using a ZORBAX Eclipse XDB-C18 column (4.6 × 150 mm, 5 μm) and UV spectroscopic detection at 254 nm. Gradient: 0-5 min 5 % B in A, 5-25 min 5-95 % B in A (A = H_2O + 0.1 % TFA, B = MeCN + 0.1 % TFA). Flow rate: 1 mL min^{-1}.

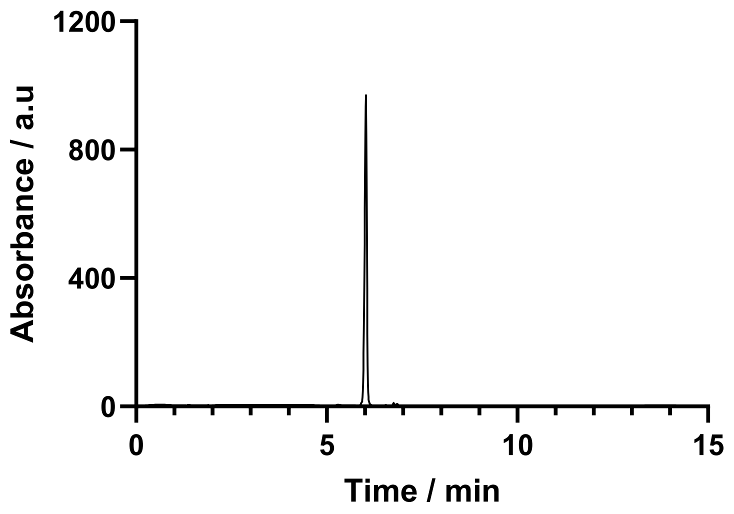

Figure S27. HPLC UV trace of **NPK_PicAm**. HPLC was performed on an Agilent 1260 Infinity II HPLC using a ZORBAX Eclipse XDB-C18 column (4.6 × 150 mm, 5 µm) and UV spectroscopic detection at 254 nm. Gradient: 0-5 min 5 % B in A, 5-25 min 5-95 % B in A (A = H_2O, B = MeCN). Flow rate: 1 mL min^{-1}.

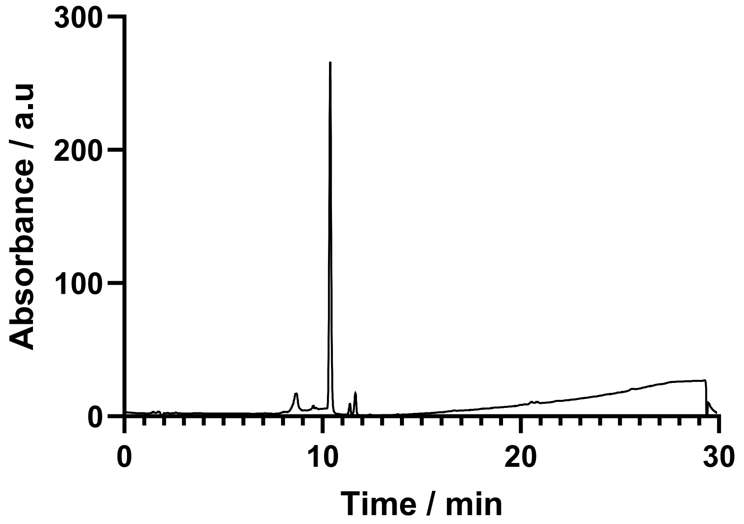

Figure S28. HPLC UV trace of **[Pb(K22_PicAm)]**$^{2+}$. HPLC was performed on an Agilent 1260 Infinity II HPLC using a ZORBAX Eclipse XDB-C18 column (4.6 × 150 mm, 5 µm) and UV spectroscopic detection at 254 nm. Gradient: 0-5 min 5 % B in A, 5-25 min 5-95 % B in A (A = H_2O + 0.1 % FA, B = MeCN + 0.1 % FA). Flow rate: 1 mL min^{-1}.

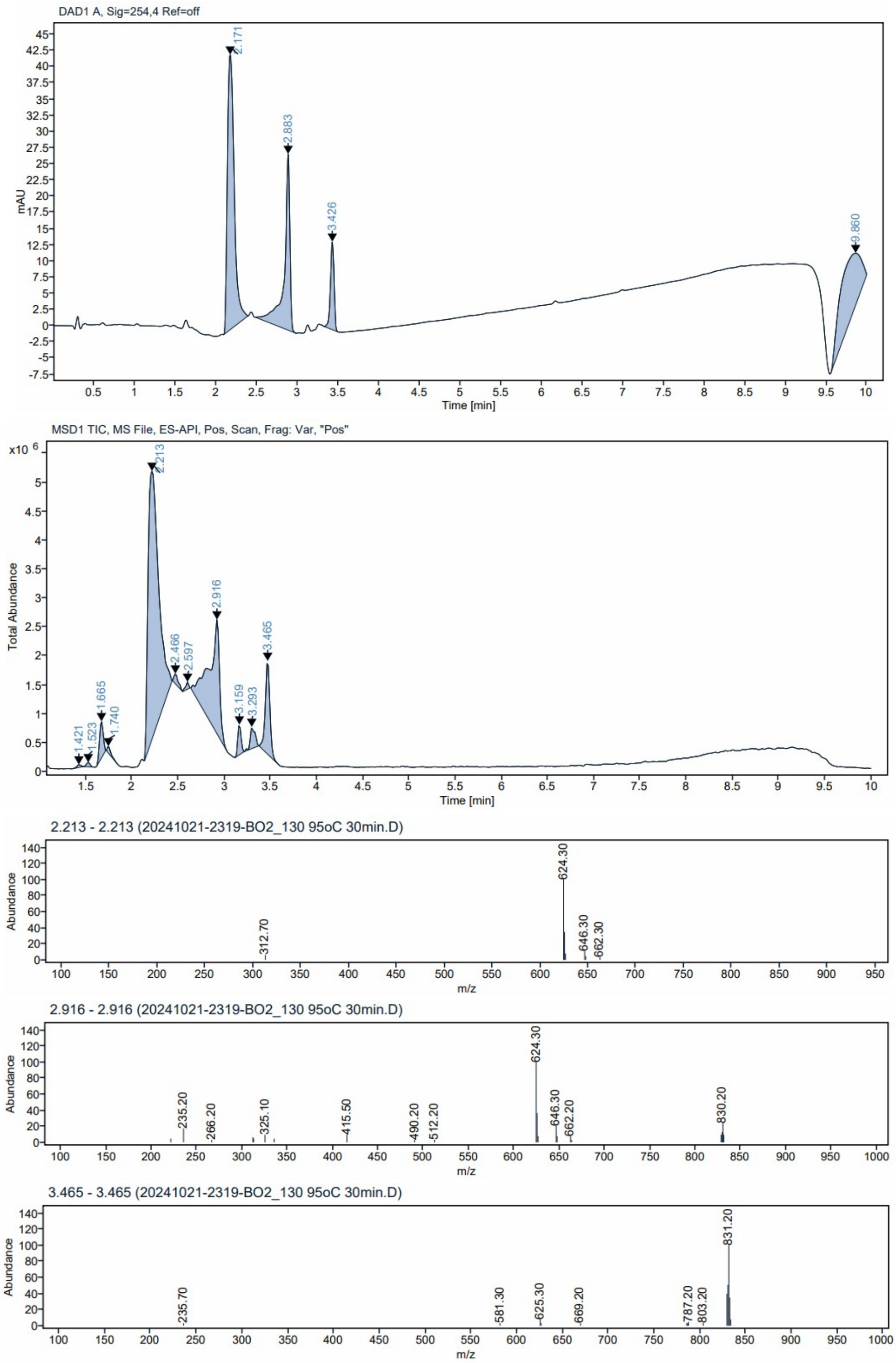

Figure S29. LCMS spectra of **[Pb(NPK_PicAm)]$^{2+}$** complexation reaction. LCMS was performed on an Agilent 1260 Infinity II HPLC with Single Quad MSD/XT MS. MS: positive and negative (dynamic), scan speed: 2600 u/s, 100-1000 m/z, cycle time: 0.84 s. For compounds more than 1000 Da; dynamic, scan speed: 2600 u/s, 500-3000 m/z, cycle time: 2.07 s. Drying gas flow and temperature: nitrogen, 12 L/min, 350 ^{o}C. Nebulizer pressure: 50 psig. Column temperature: 40 ^{o}C. Mobile phase A contained H_2O with 0.1 % formic acid, and mobile phase B contained MeCN with 0.1 % formic acid. Analytical reverse-phase LCMS was conducted using an Agilent Poroshell HPH C18 column (3.0×0.50 mm, 2.7 µm) and UV spectroscopic detection at 210 nm and 360 nm. Eluent gradient: 0 % B in A, 0-100 % B in A for 7 minutes, 0-100 % B in A for 1 minute, 100-50 % B in A for 0.17 minutes, 50 % B in A for 1.83 minutes; flow rate 0.8 mL min^{-1}.

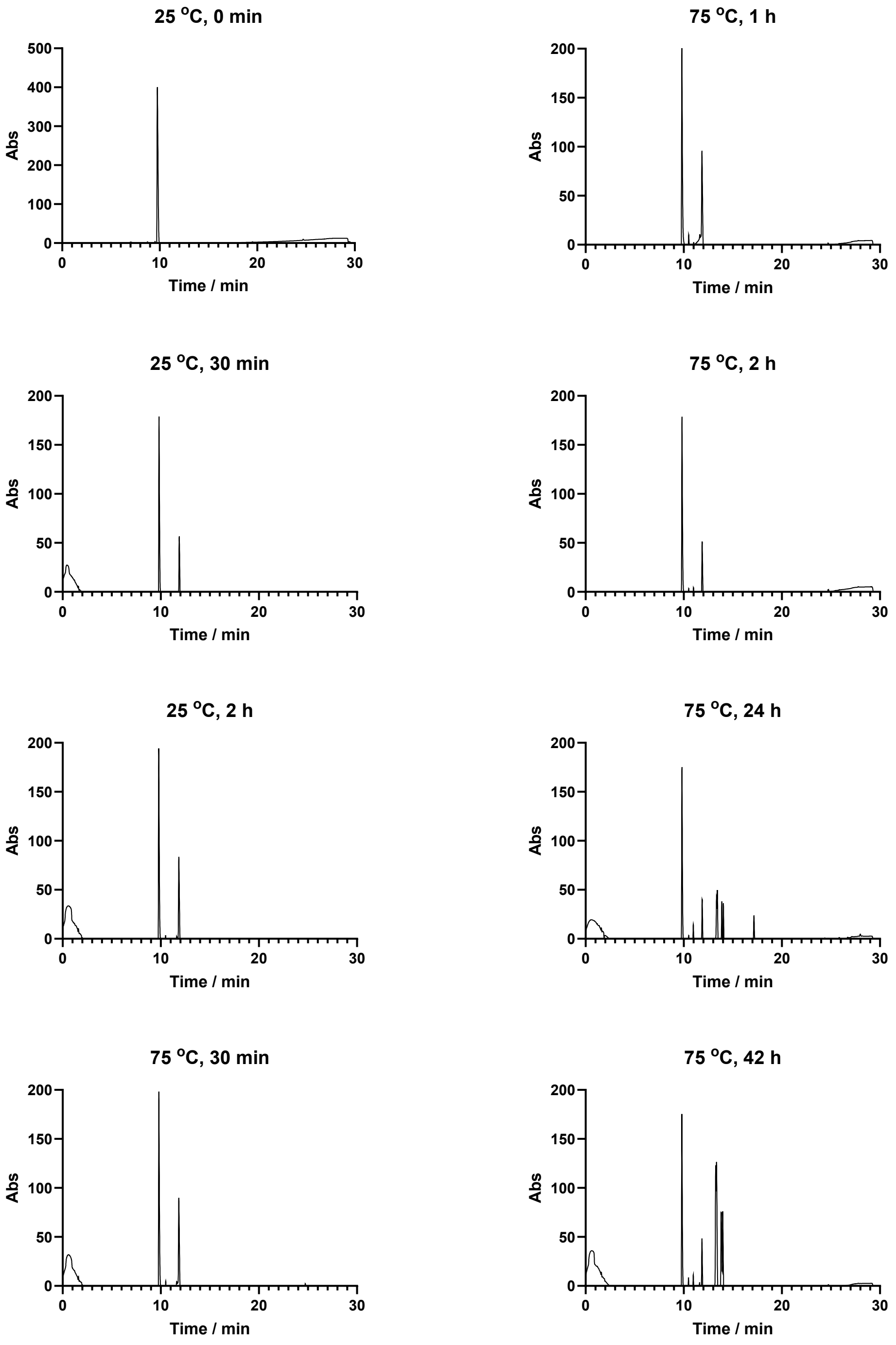

Figure S30. HPLC UV trace of $Pb(OAc)_2.3H_2O$ complexation with **NPK_PicAm**. HPLC was performed on an Agilent 1260 Infinity II HPLC using a ZORBAX Eclipse XDB-C18 column (4.6 × 150 mm, 5 μm) and UV spectroscopic detection at 254 nm. Gradient: 0-5 min 5 % B in A, 5-25 min 5-95 % B in A (A = H_2O + 0.1 % FA, B = MeCN + 0.1 % FA). Flow rate: 1 mL min^{-1}.

9. Potentiometric Titration and UV-Vis Spectroscopy Measurements

Potentiometric titrations were carried out using a Metrohm Titrando 888 titrator equipped with a combined glass electrode (Metrohm model 6.0260.010) and a Metrohm 801 Stirrer unit. This titration system was controlled by *Tiamo* (ver. 2.34) software. The titration vessel was fitted into a removable

glass cell (≈ 70 mL) and thermostated at 25.0 °C (p*K*w (NaCl, 0.1 M) = 13.78)[4] using a circulating water bath. CO_2 was excluded from the vessel prior to and during the titrations by a nitrogen flow, which was passed through an aqueous solution. NaOH (0.1 M) was standardized by potentiometric titration against potassium hydrogen phthalate. Hydrochloric acid (0.1 M,) was standardized against Tris (base form). Titration solutions were maintained at a constant ionic strength of 0.1 M with NaCl (prepared by adding 5.8 g NaCl to 1 L de-ionised H_2O). The electrode was calibrated before each titration by titrating a solution of standardized HCl with standardized NaOH, and the data were analysed using the program *GLEE*5 (ver. 3.0.21)[5] to obtain the standard electrode potential and slope factor.
Pb^{2+} stock solution was made by dissolving the corresponding perchlorate salts (98 % purity or higher) in H_2O. The exact concentrations were determined by complexometric titrations[6] with a standardized 0.01 M EDTA solution. The complexometric titrations were performed in water, and the end point was indicated by Eriochrome Black T. Ligand aliquots were added directly into the electrolyte solution prior to analysis.

Protonation constant or stability constant determinations were carried out by titrating an acidic solution containing free ligand or both ligand and metal (in 30 mL 0.1 M NaCl) with standardized 0.1 M NaOH. For protonation constant determinations, the amount of ligand used was 0.02 mmol. For stability constant determinations, the amount of ligand used was 0.02 mmol and amount of metal ion used was 0.018 mmol. The solutions were inspected throughout the titrations for signs of $Pb(OH)_2$ precipitation. The titration data were refined with *Hyperquad 2013*[4] software to determine protonation and stability constants. The data points within the pH range of 2.6–11.3 were used for analysis. At least three independent titrations for protonation constant measurements and three titrations for stability constant measurements were carried out for each L and PbL system.

Equilibration between the addition of NaOH aliquots was monitored via the electrode potential. If the system is not at equilibrium, the electrode potential will drift as the system continues to adjust. To ensure that the thermodynamic equilibration is reached after an aliquot of NaOH is added, the next NaOH aliquot was not added until either the electrode drifting is slow (< 0.2 mV/min), or a maximum time limit is reached (2 min for both protonation constant determinations and stability constant determinations).

The UV-Vis spectra were recorded using a Cary 60 spectrophotometer. The water was distilled twice and passed through a Millipore apparatus. The pH values were measured using a Metler-Toledo pH meter, and the hydrogen ion concentration of the solutions was adjusted using diluted HCl and NaOH solutions. The ligand concentration in the working solutions was determined by titration with known amounts of lead(II) at fixed pH values of 3 and 7, at which quantitative formation of the complexes occurs. To fix the pH at 7, a Tris-buffered solution was employed in some of the titrations; see Fig. S39, for examples.

To assess complex thermodynamic stability, the ligand protonation constants ($\log K_i$) and Pb-ligand stability complexes ($\log K_{PbL}$) were determined via potentiometric titrations.
Protonation and stability constants are defined in **Equations S1** and **S2** respectively, where (L) represents the equilibrium concentrations of fully deprotonated ligand.

$$K_i = \frac{[H_iL]}{[H^+][H_i - 1L]}$$

Equation S1:
i = number of protons

$$K_{PbL} = \frac{[PbL]}{[Pb^{2+}][L]}$$

Equation S2:
L = K_{22}PicAm or NPK_PicAm

Potentiometric titrations use the change in measured potential to calculate protonation (K_a) and stability constants (K_{PbL}) using the Nernst Equation (**Equation S3**). The E^0 is measured during the calibration measurements, and the E is the measured potential during the experiment. At defined temperature and ionic strength conditions, log K values can be calculated.

$$E = E^0 - \frac{2.303RT}{nF} log_{10}K$$

Equation S3: The Nernst Equation used for the calculation of $log_{10}K$
E = measured difference in potential
E^0 = calibration value for difference in potential
n = number of protons transferred

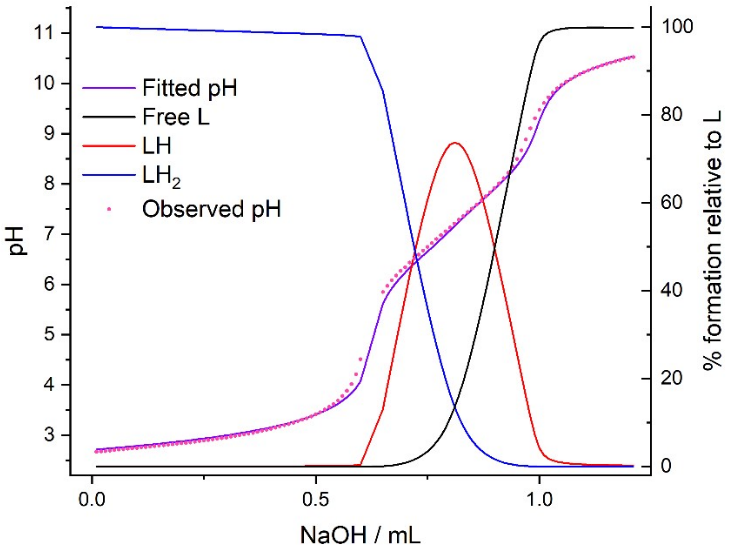

Figure S31. Representative protonation constant determination of **K22_PicAm** by potentiometric titrations. C_{K22_PicAm} = 0.02 mmol. Initial volume V = 31.5 mL. Data fitting and speciation distribution over the pH range shown.

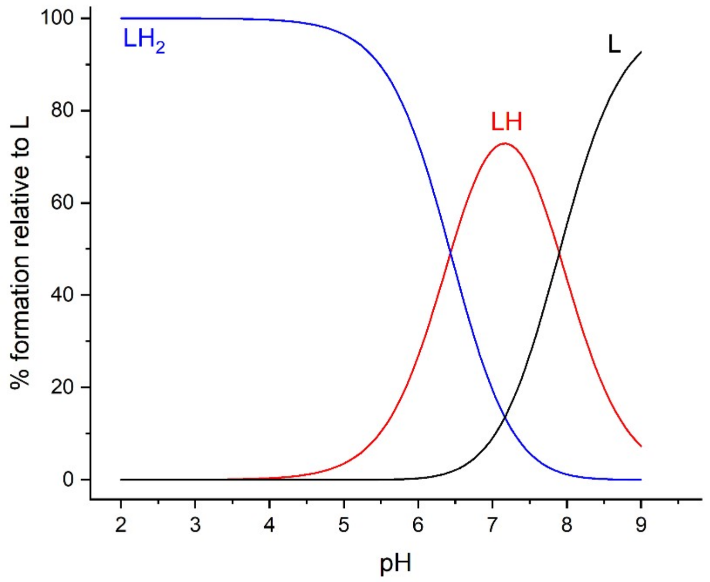

Figure S32. Representative species distribution of **K22_PicAm** modelled in HySS. C_{K22_PicAm} = 0.02 mmol. Initial volume V = 31.5 mL. Data fitting and speciation distribution over the pH range shown.

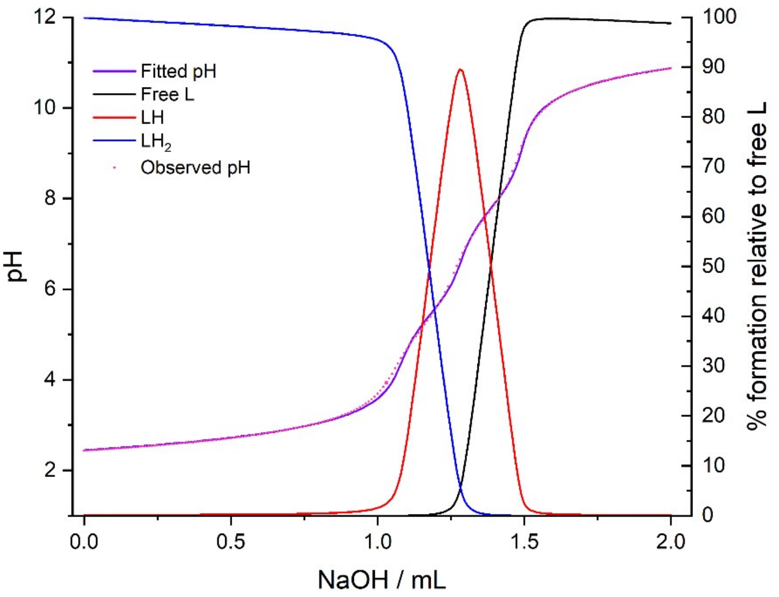

Figure S33. Representative protonation constant determination of **NPK_PicAm** by potentiometric titrations. C_{NPK_PicAm} = 0.02 mmol. Initial volume V = 31.5 mL. Data fitting and speciation distribution over the pH range shown.

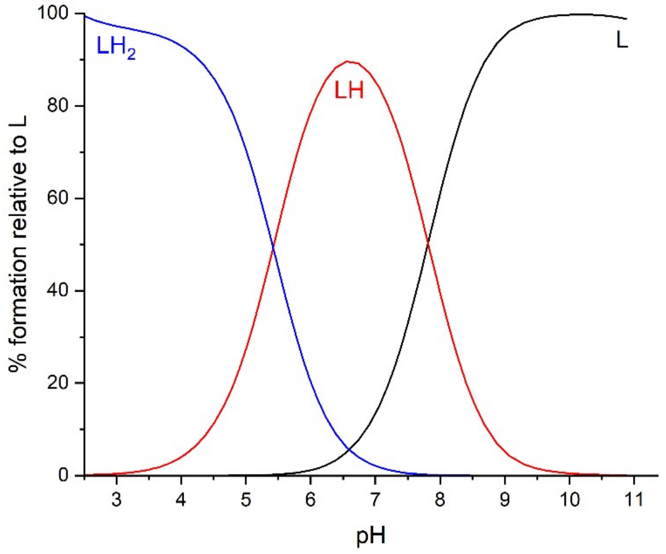

Figure S34. Representative species distribution of **NPK_PicAm** modelled in HySS. C_{NPK_PicAm} = 0.02 mmol. Initial volume V = 31.5 mL. Data fitting and speciation distribution over the pH range shown.

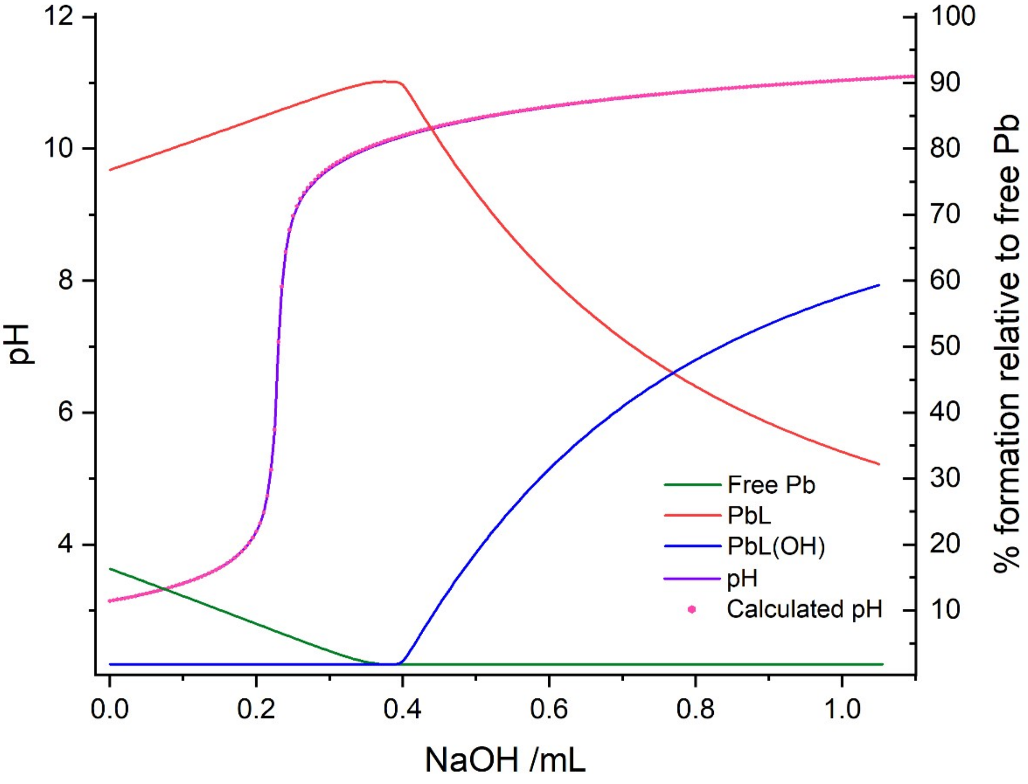

Figure S35. Representative stability constant determination of **[Pb(NPK_PicAm)]$^{2+}$** by potentiometric titrations. C_{K22_PicAm} = 0.02 mmol. C_{Pb} = 0.018 mmol. Initial volume V = 30.6 mL. Data fitting and speciation distribution over the pH range shown.

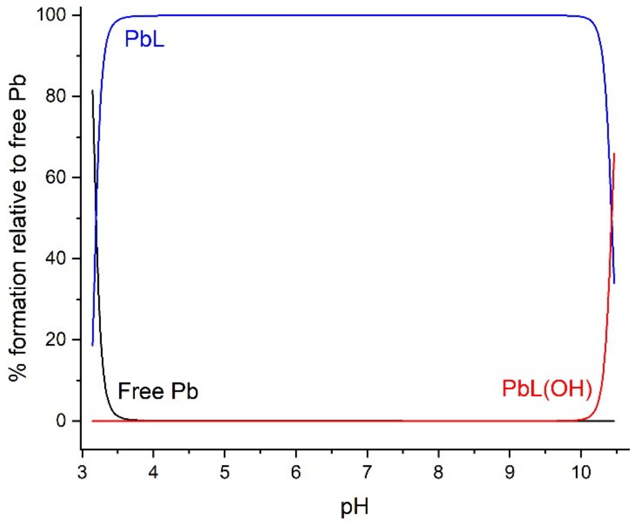

Figure S36. Representative species distribution of **[Pb(NPK_PicAm)]$^{2+}$** modelled in HySS. C_{K22_PicAm} = 0.02 mmol. C_{Pb} = 0.018 mmol. Initial volume V = 30.6 mL. Data fitting and speciation distribution over the pH range shown.

A

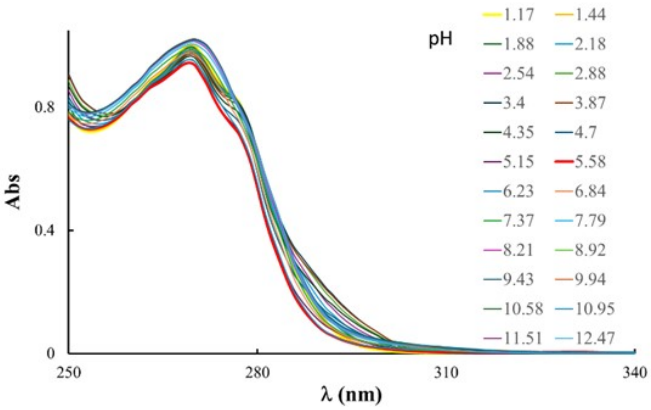

B

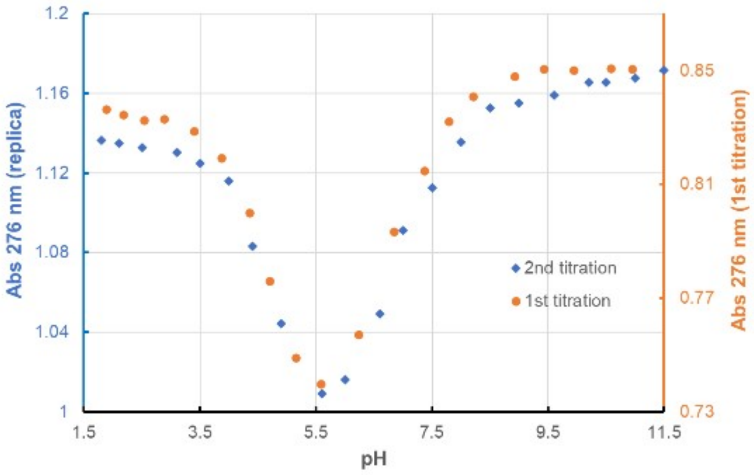

Figure S37. A) Changes in the absorption spectra of a 7.4x10^{-5} M solution of **K22_PicAm** in 0.15 M NaCl at different pH values (repeat 2). B) Representation of the absorbance at 276 nm vs. pH showing the values obtained from different repeats (repeat 1, blue dots; repeat two, orange dots).

A **B**

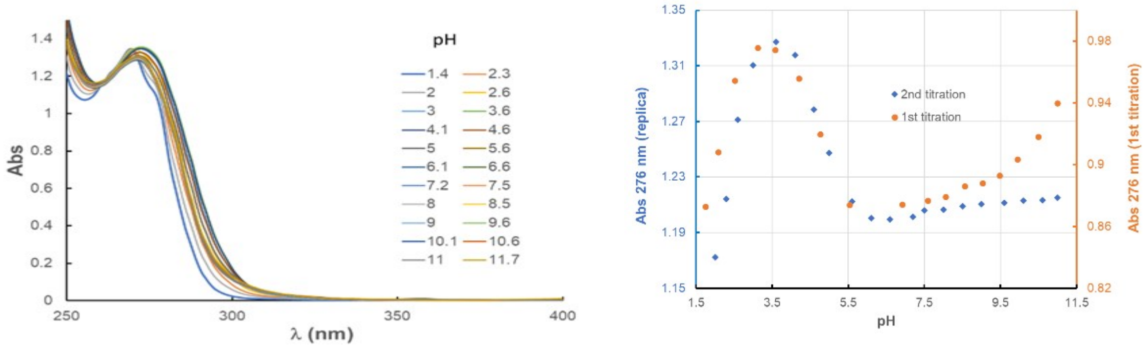

Figure S38. A) Changes in the absorption spectra of a 7.4x10^{-5} M solution of **K22_PicAm** containing an equimolar amount of Pb^{2+} in 0.15 M NaCl at different pH values (repeat 2). B) Representation of the absorbance at 276 nm vs pH showing the values obtained from different repeats (repeat 1, blue dots; repeat two, orange dots).

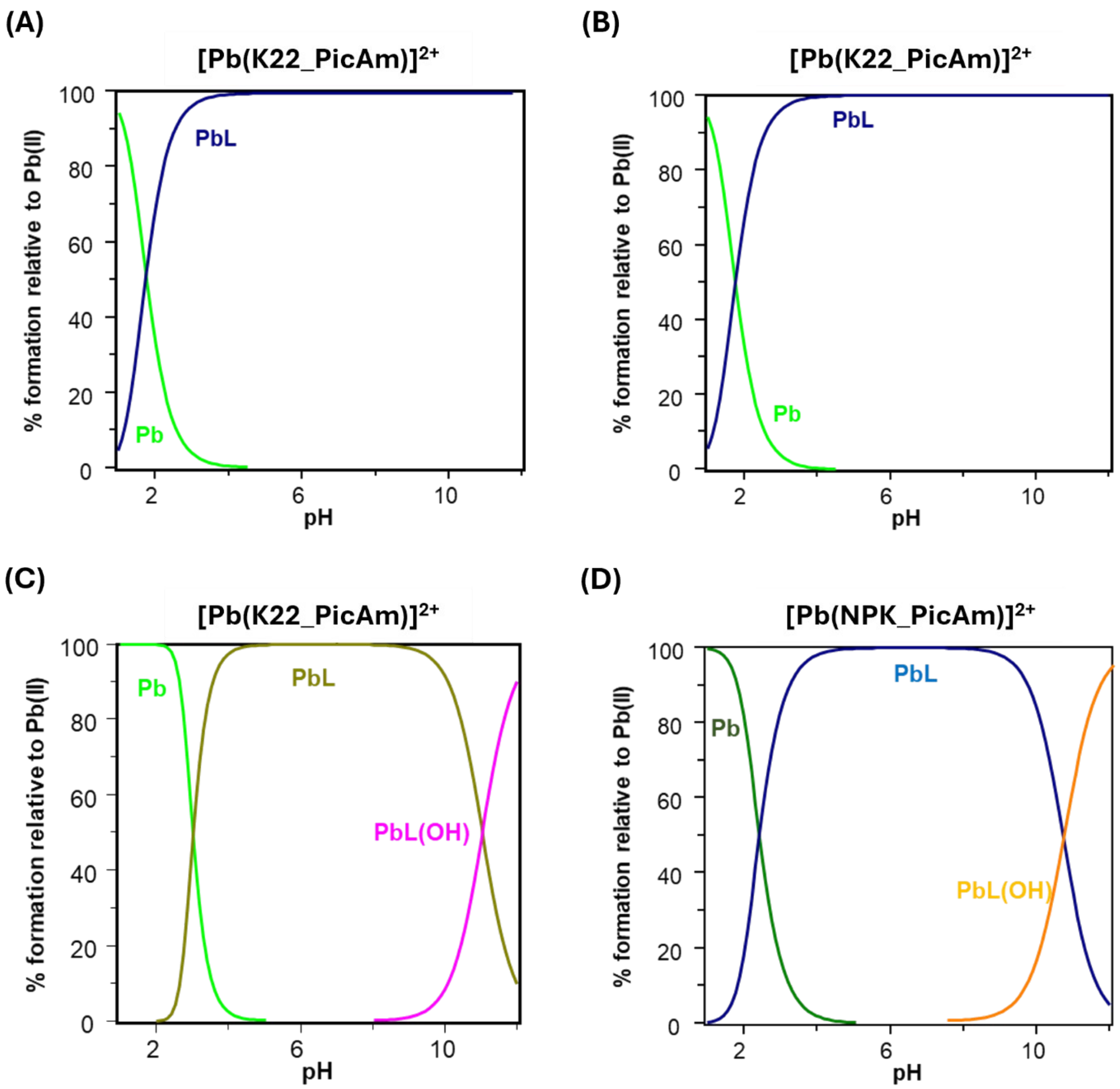

Figure S39. Speciation diagram of the system Pb^{2+}-L calculated from the stability constants determined from the spectroscopic data $[Pb^{2+}] = [L] = 1x10^{-3}$ M. A) Potentiometric data. B) Data from Regueiro-Figueroa *et al.*[3] C) Data using values from the four-protonation constant UV model. D) Species distribution for the system **Pb^{2+}-NPK_Picam**.

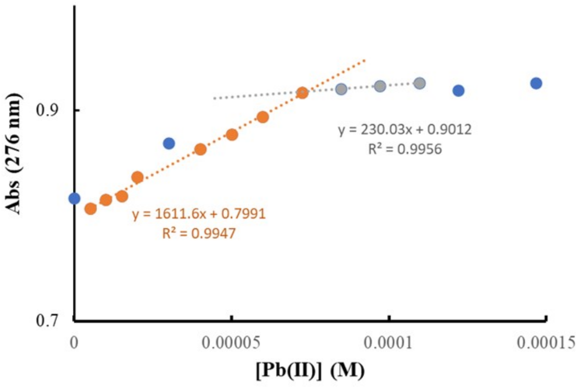

Figure S40. Example of a titration of the ligand with a Pb^{2+} solution at constant pH. Dots represent the absorbance at 276 nm.

10. Crystal Data and Structure Refinement Details for [Pb(K22_PicAm)]-$2PF_6$

Single crystals of **[Pb(K22_PicAm)]-$2PF_6$** suitable for X-ray diffraction studies were isolated from the addition of excess NH_4PF_6 to an aqueous solution of **[Pb(K22_PicAm)][OAc]$_2$**, which was concentrated under reduced pressure, dissolved in DMF and slowly evaporated.

Table S2. Crystal data and structure refinement for **[Pb(K22_PicAm)]-$2PF_6$** (NL2416).

Identification code	NL2416	
Formula	C26 H38 N6 O6 Pb, 2(F6 P), 1.75(C3 H7 N O)	
Formula weight	1155.67	
Temperature	173(2) K	
Diffractometer, wavelength	Agilent Xcalibur PX Ultra A, 1.54184 Å	
Crystal system, space group	Tetragonal, I4/m	
Unit cell dimensions	a = 28.02150(16) Å	a = 90°
	b = 28.02150(16) Å	b = 90°
	c = 22.63094(16) Å	g = 90°
Volume, Z	17769.9(2) Å^3, 16	
Density (calculated)	1.728 Mg/m^3	
Absorption coefficient	9.011 mm^{-1}	
F(000)	9184	
Crystal colour / morphology	Colourless blocks	
Crystal size	0.411 x 0.083 x 0.073 mm^3	
q range for data collection	3.907 to 73.547°	
Index ranges	-34<=h<=34, -34<=k<=34, -27<=l<=28	
Reflns collected / unique	138305 / 9170 [R(int) = 0.0510]	
Reflns observed [F>4s(F)]	6638	
Completeness to theta(full)	0.998 to 67.684 deg	
Absorption correction	Analytical	
Max. and min. transmission	0.576 and 0.123	
Refinement method	Full-matrix least-squares on F^2	
Data / restraints / parameters	9170 / 0 / 352	
Goodness-of-fit on F^2	1.046	
Final R indices [F>4s(F)]	R1 = 0.0372, wR2 = 0.1002	
R indices (all data)	R1 = 0.0551, wR2 = 0.1193	

Largest diff. peak, hole	1.539, -0.640 eÅ^{-3}
Mean and maximum shift/error	0.000 and 0.003

Outside of the lead complex itself, the structure of **[Pb(K22_PicAm)]-2PF$_6$** was found to be significantly disordered. The solvent could not be identified, and even allowing for some less than ideal thermal ellipsoids only *ca*. 1.88 of the expected 2PF$_6$ anions could be located, distributed across seven sites ranging from general to 4/*m* special positions. Given that the complex looked to be the only ordered portion of the overall structure, it was decided to use the SQUEEZE routine of PLATON[4] to remove everything that was not the complex. This has the downside of losing detail for all the removed electron density (though the extensive disorder meant that detail was already somewhat lacking) whilst having the upside of allowing for a more reliable determination of the geometry of the complex itself, which is the most interesting part of the structure.

Using SQUEEZE like this suggested a total of 3380 electrons per unit cell, equivalent to 211.3 electrons per complex. Accounting for the electron density of the presumed 2PF$_6$ anions (2 x 69 = 138 electrons) leaves 211.3 – 138 = 73.3 electrons for the solvent. The most recently used solvent was dimethylformamide (C_3H_7NO, 40 electrons), and 1.75 dimethylformamide molecules correspond to 70 electrons, so this was used as the solvent present. As a result, the atom list for the asymmetric unit is low by 2(PF6) + 1.75(C_3H_7NO) = $C_{5.25}H_{12.25}F_{12}N_{1.75}O_{1.75}P_2$ (and that for the unit cell low by $C_{84}H_{196}F_{192}N_{28}O_{28}P_{32}$) compared to what is actually presumed to be present.

The presumed NH_2 hydrogen atoms on N17 and N36 could not be located from ΔF maps, and so were added in calculated positions with an N–H distance of 0.90 Å.

XRD References

[1] O.V. Dolomanov, L.J. Bourhis, R.J. Gildea, J.A.K. Howard, H. Puschmann, *J. Appl. Cryst.*, 2009, **42**, 339-341.

[2] SHELXTL v5.1, Bruker AXS, Madison, WI, 1998.

[3] SHELX-2013, G.M. Sheldrick, *Acta Cryst.*, 2015, **C71**, 3-8.

[4] A.L. Spek (2003, 2009) PLATON, A Multipurpose Crystallographic Tool, Utrecht University, Utrecht, The Netherlands. See also A.L. Spek, *Acta. Cryst.*, 2015, **C71**, 9-18.

Table S3. Bond lengths (Å) and angles (°) for **[Pb(K22_PicAm)]-2PF$_6$** (NL2416).

Pb(1)-O(26)	2.534(4)
Pb(1)-O(35)	2.604(4)
Pb(1)-N(30)	2.613(5)
Pb(1)-N(21)	2.679(5)
Pb(1)-N(10)	2.890(5)
Pb(1)-O(7)	2.891(4)
Pb(1)-O(16)	2.910(4)
Pb(1)-O(13)	2.925(4)
Pb(1)-O(4)	3.015(4)
Pb(1)-N(1)	3.045(5)
N(1)-C(19)	1.450(8)
N(1)-C(2)	1.466(9)
N(1)-C(18)	1.468(9)
C(2)-C(3)	1.468(11)
C(3)-O(4)	1.426(8)
O(4)-C(5)	1.414(8)

C(5)-C(6)	1.508(10)
C(6)-O(7)	1.395(8)
O(7)-C(8)	1.428(8)
C(8)-C(9)	1.501(10)
C(9)-N(10)	1.473(9)
N(10)-C(11)	1.485(8)
N(10)-C(28)	1.486(8)
C(11)-C(12)	1.519(11)
C(12)-O(13)	1.423(8)
O(13)-C(14)	1.425(8)
C(14)-C(15)	1.499(11)
C(15)-O(16)	1.402(8)
O(16)-C(17)	1.425(9)
C(17)-C(18)	1.512(11)
C(19)-C(20)	1.510(10)
C(20)-N(21)	1.340(8)
C(20)-C(25)	1.372(10)
N(21)-C(22)	1.351(8)
C(22)-C(23)	1.389(9)
C(22)-C(26)	1.466(9)
C(23)-C(24)	1.402(12)
C(24)-C(25)	1.346(13)
O(26)-C(26)	1.235(7)
C(26)-N(27)	1.335(8)
C(28)-C(29)	1.502(8)
C(29)-N(30)	1.349(7)
C(29)-C(34)	1.371(9)
N(30)-C(31)	1.332(7)
C(31)-C(32)	1.405(9)
C(31)-C(35)	1.498(8)
C(32)-C(33)	1.354(9)
C(33)-C(34)	1.395(10)
O(35)-C(35)	1.236(8)
C(35)-N(36)	1.341(8)
O(26)-Pb(1)-O(35)	113.92(13)
O(26)-Pb(1)-N(30)	69.10(15)
O(35)-Pb(1)-N(30)	61.05(14)
O(26)-Pb(1)-N(21)	61.45(15)
O(35)-Pb(1)-N(21)	71.52(14)
N(30)-Pb(1)-N(21)	82.74(16)
O(26)-Pb(1)-N(10)	75.61(16)
O(35)-Pb(1)-N(10)	109.49(14)
N(30)-Pb(1)-N(10)	59.72(14)
N(21)-Pb(1)-N(10)	131.06(15)
O(26)-Pb(1)-O(7)	135.31(14)
O(35)-Pb(1)-O(7)	78.63(14)
N(30)-Pb(1)-O(7)	83.22(14)
N(21)-Pb(1)-O(7)	150.14(14)
N(10)-Pb(1)-O(7)	60.05(15)
O(26)-Pb(1)-O(16)	75.94(15)
O(35)-Pb(1)-O(16)	132.72(14)
N(30)-Pb(1)-O(16)	144.45(15)
N(21)-Pb(1)-O(16)	74.99(14)
N(10)-Pb(1)-O(16)	117.63(15)
O(7)-Pb(1)-O(16)	128.35(13)
O(26)-Pb(1)-O(13)	67.01(12)
O(35)-Pb(1)-O(13)	170.11(13)
N(30)-Pb(1)-O(13)	111.98(13)
N(21)-Pb(1)-O(13)	115.78(14)
N(10)-Pb(1)-O(13)	60.81(14)

O(7)-Pb(1)-O(13)	93.91(13)
O(16)-Pb(1)-O(13)	57.13(14)
O(26)-Pb(1)-O(4)	166.53(14)
O(35)-Pb(1)-O(4)	70.89(13)
N(30)-Pb(1)-O(4)	122.48(14)
N(21)-Pb(1)-O(4)	110.85(14)
N(10)-Pb(1)-O(4)	115.45(15)
O(7)-Pb(1)-O(4)	56.99(13)
O(16)-Pb(1)-O(4)	91.58(14)
O(13)-Pb(1)-O(4)	110.58(11)
O(26)-Pb(1)-N(1)	110.44(16)
O(35)-Pb(1)-N(1)	75.22(14)
N(30)-Pb(1)-N(1)	128.78(14)
N(21)-Pb(1)-N(1)	57.87(15)
N(10)-Pb(1)-N(1)	170.53(15)
O(7)-Pb(1)-N(1)	114.24(15)
O(16)-Pb(1)-N(1)	58.81(15)
O(13)-Pb(1)-N(1)	114.08(14)
O(4)-Pb(1)-N(1)	57.59(14)
C(19)-N(1)-C(2)	109.8(6)
C(19)-N(1)-C(18)	109.8(6)
C(2)-N(1)-C(18)	110.5(6)
C(19)-N(1)-Pb(1)	101.7(4)
C(2)-N(1)-Pb(1)	113.7(4)
C(18)-N(1)-Pb(1)	110.9(4)
N(1)-C(2)-C(3)	114.8(6)
O(4)-C(3)-C(2)	108.9(6)
C(5)-O(4)-C(3)	110.0(6)
C(5)-O(4)-Pb(1)	112.0(4)
C(3)-O(4)-Pb(1)	113.2(4)
O(4)-C(5)-C(6)	109.0(5)
O(7)-C(6)-C(5)	108.1(6)
C(6)-O(7)-C(8)	110.8(5)
C(6)-O(7)-Pb(1)	119.2(4)
C(8)-O(7)-Pb(1)	118.5(4)
O(7)-C(8)-C(9)	108.5(5)
N(10)-C(9)-C(8)	113.4(6)
C(9)-N(10)-C(11)	110.3(6)
C(9)-N(10)-C(28)	110.2(6)
C(11)-N(10)-C(28)	108.6(5)
C(9)-N(10)-Pb(1)	111.1(4)
C(11)-N(10)-Pb(1)	112.5(4)
C(28)-N(10)-Pb(1)	104.0(3)
N(10)-C(11)-C(12)	114.2(6)
O(13)-C(12)-C(11)	107.9(5)
C(12)-O(13)-C(14)	108.5(5)
C(12)-O(13)-Pb(1)	114.4(4)
C(14)-O(13)-Pb(1)	113.3(4)
O(13)-C(14)-C(15)	109.5(5)
O(16)-C(15)-C(14)	110.0(5)
C(15)-O(16)-C(17)	110.3(6)
C(15)-O(16)-Pb(1)	120.4(4)
C(17)-O(16)-Pb(1)	119.3(4)
O(16)-C(17)-C(18)	109.1(5)
N(1)-C(18)-C(17)	114.1(6)
N(1)-C(19)-C(20)	112.3(6)
N(21)-C(20)-C(25)	122.0(8)
N(21)-C(20)-C(19)	117.2(6)
C(25)-C(20)-C(19)	120.8(7)
C(20)-N(21)-C(22)	119.4(6)

C(20)-N(21)-Pb(1)	123.3(5)
C(22)-N(21)-Pb(1)	116.8(4)
N(21)-C(22)-C(23)	120.7(7)
N(21)-C(22)-C(26)	114.0(5)
C(23)-C(22)-C(26)	125.1(7)
C(22)-C(23)-C(24)	118.7(8)
C(25)-C(24)-C(23)	119.4(8)
C(24)-C(25)-C(20)	119.9(8)
C(26)-O(26)-Pb(1)	121.6(4)
O(26)-C(26)-N(27)	121.4(6)
O(26)-C(26)-C(22)	121.4(6)
N(27)-C(26)-C(22)	117.1(6)
N(10)-C(28)-C(29)	112.0(5)
N(30)-C(29)-C(34)	121.1(6)
N(30)-C(29)-C(28)	116.4(5)
C(34)-C(29)-C(28)	122.4(5)
C(31)-N(30)-C(29)	117.9(5)
C(31)-N(30)-Pb(1)	118.2(4)
C(29)-N(30)-Pb(1)	122.6(4)
N(30)-C(31)-C(32)	123.8(5)
N(30)-C(31)-C(35)	112.9(5)
C(32)-C(31)-C(35)	123.2(5)
C(33)-C(32)-C(31)	117.7(6)
C(32)-C(33)-C(34)	118.8(7)
C(29)-C(34)-C(33)	120.6(6)
C(35)-O(35)-Pb(1)	114.9(4)
O(35)-C(35)-N(36)	122.0(6)
O(35)-C(35)-C(31)	119.7(5)
N(36)-C(35)-C(31)	118.4(6)

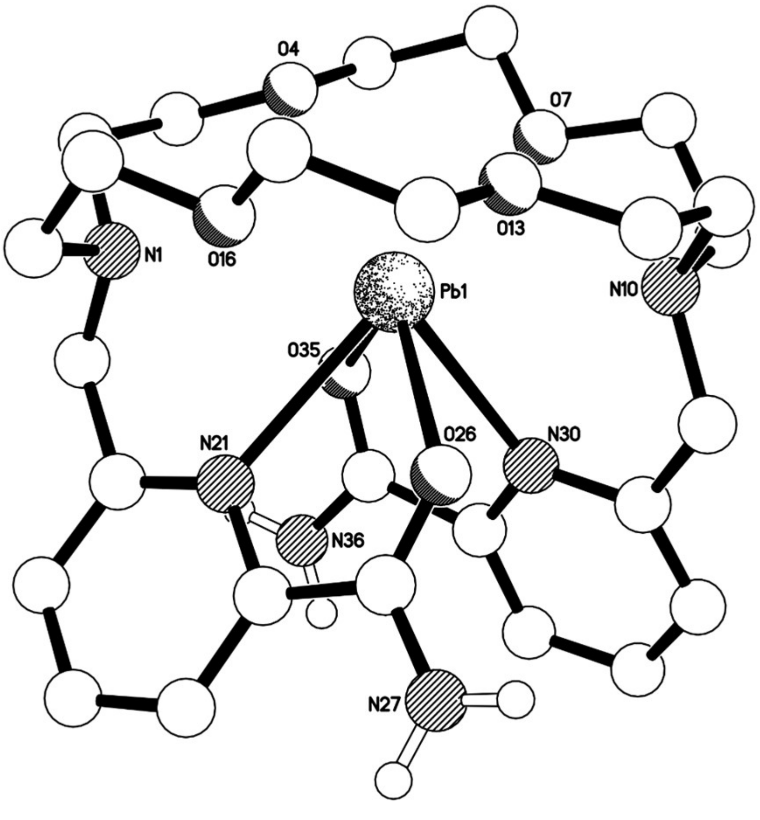

Figure S41. The structure of the cationic complex present in the crystal of **[Pb(K22_PicAm)]-2PF$_6$** (NL2416).

11. Density Functional Theory (DFT) Calculations

Details

Geometry optimizations using density functional theory (DFT) were performed in Gaussian16 (Revision C.01.).[5] Initial geometries for **K22_PicAm** and **NPK_PicAm** and respective Pb^{2+} complexes, were taken from the crystal structure of **macropa** with modification of picolinate arms to the picolinamide arms and addition of a nitrophenyl group in the macrocyclic ring.[6] The hybrid ωB97XD functional was employed,[7,8] which includes both dispersion and long-range corrections. Specifically For C, H, N and O atoms the def2tzvp (triple ζ) basis set was utilised.[9] For Pb the Stuttgart-Dresden SDD effective core potential (ECP) and associated basis set was used (keyword=SDD).[10] Geometry optimisations were performed without symmetry constrains (nosymm) and improved numerical integration grid using a pruned grid with 99 radial shells and 590 angular points per shell (int=ultrafine) was used. Additionally, frequency calculations were carried out on optimised structure to confirm, via the absence of imaginary frequencies, that the structures are local minima on the potential energy surface. To simulate the aqueous environment, single point energy corrections using the universal solvation model (SMD)[11] were carried out using water as the solvent. Natural Bond Orbital analysis was carried out in NBO 7.0.[12]

Thermal corrections and solution phase corrections were applied using Paton's *GoodVibes* software,[13] which incorporates Grimme's quasi-harmonic approximation to the vibrational entropy below a cut-off of 100 cm^{-1} and a frequency scaling factor = 1.0. The entropic terms for the frequencies below the cut-off are obtained from the free-rotor approximation while the standard rigid-rotor harmonic oscillator (RRHO) approximation is retained for those above the cut-off. A dampening function is used to interpolate between these two expressions close to the cut-off frequency. A temperature of 298 K and concentration of 1 M for [L] and 55.5 M (concentration of pure water) for H_2O was used to correct the gas-phase standard state free energies ($G°_{gas}$: 298 K, 1 atm) into solution-phase standard state free energies ($G°_{gas}$: 298 K, 1 mol/L) by the relationship with the ideal gas law (1 atm = 1/24.5 M at 298 K).

Methods

For the Gibbs energy of complexation:

$$Equation\ S4: \Delta G°_{[PbL]} = G°_{aq}([PbL]^{2+}) - G°_{aq}(Pb^{2+}) - G°_{aq}(L)$$

Table S4. Free energies in aqueous solution ($G°_{aq}$., kcal mol^{-1}) of DFT optimised structures and the Gibbs free energy changes ($\Delta G°_{aq}$., kcal mol^{-1}) described by **Equation S4**. a: $G°_{aq}$(K22_PicAm) = -1792.490822 kcal mol^{-1}; b: $G°_{aq}$(NPK_PicAm) = -2149.398076 kcal mol^{-1}.

	$G°_{aq.}$(PbK22_PicAm)	$G°_{aq.}$(PbNPK_PicAm)	$G°_{aq.}(Pb^{2+})$	$\Delta G°_{PbK22\_PicAm}{}^{a}$	$\Delta G°_{PbNPK\_PicAm}{}^{b}$
Pb-*syn*	-1795.734352	-2152.648295	-3.189201	-34.09193646	-38.28934416
Pb-*anti*	-1795.733969	-2152.645044		-33.85160051	-36.2493124

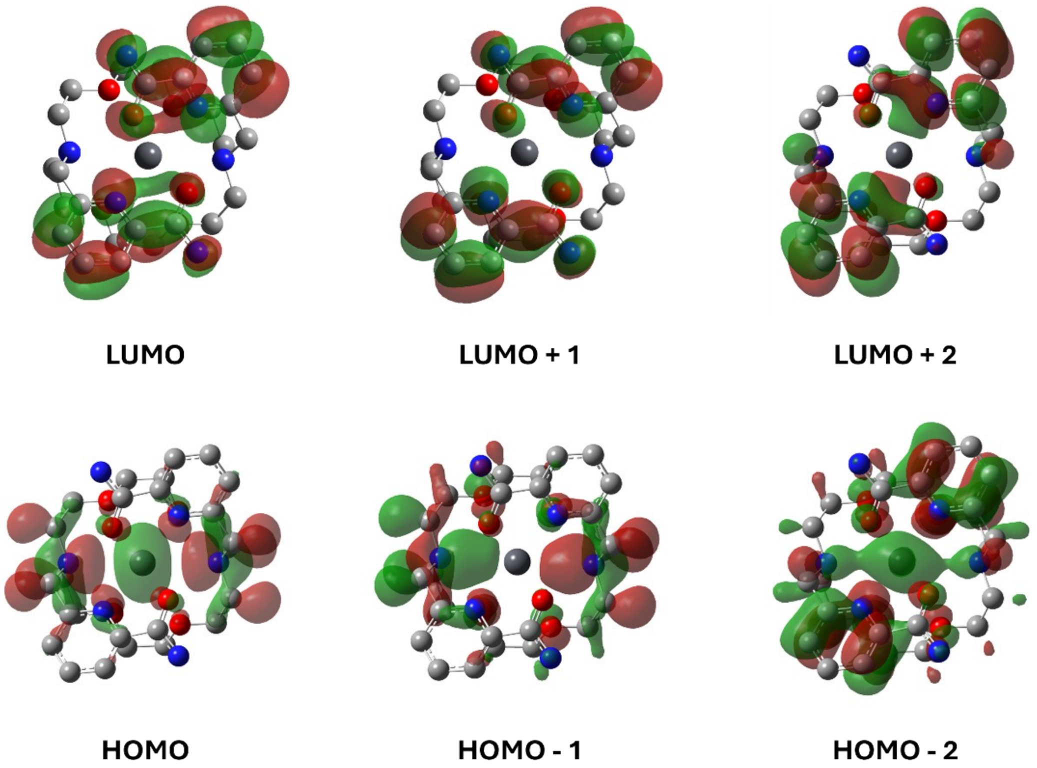

Figure S42. Representation of the HOMO, HOMO-1, HOMO-2, LUMO, LUMO+1, and LUMO+2 of ***syn*-[Pb(K22_PicAm)]$^{2+}$**.

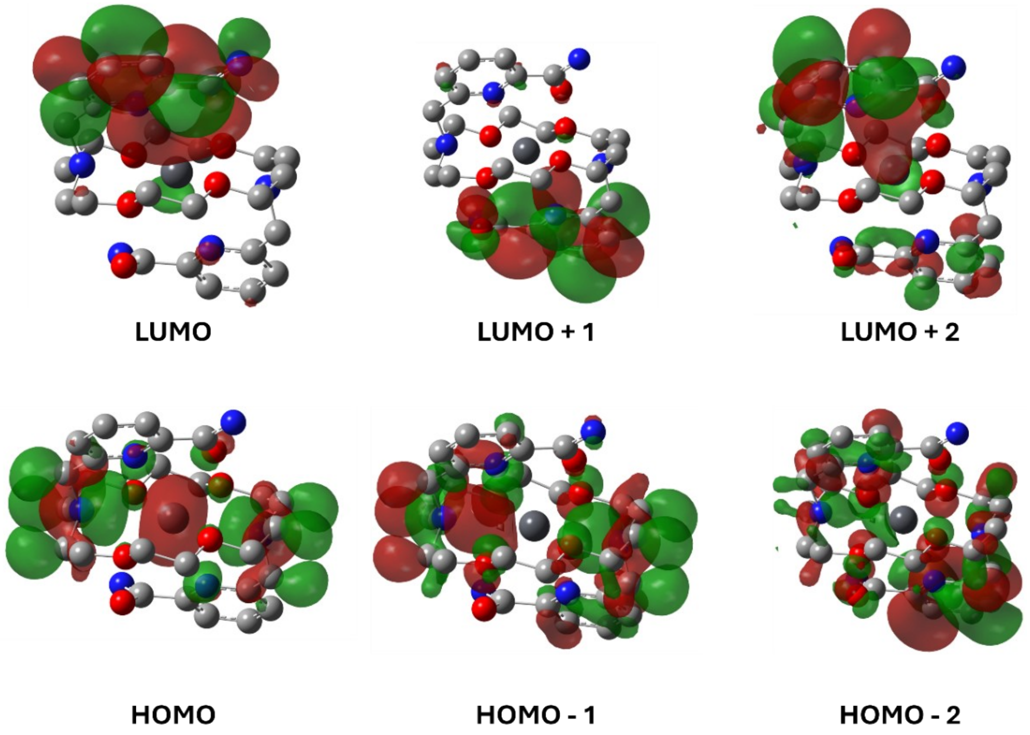

Figure S43. Representation of the HOMO, HOMO-1, HOMO-2, LUMO, LUMO+1, and LUMO+2 of ***anti*-[Pb(K22_PicAm)]$^{2+}$**.

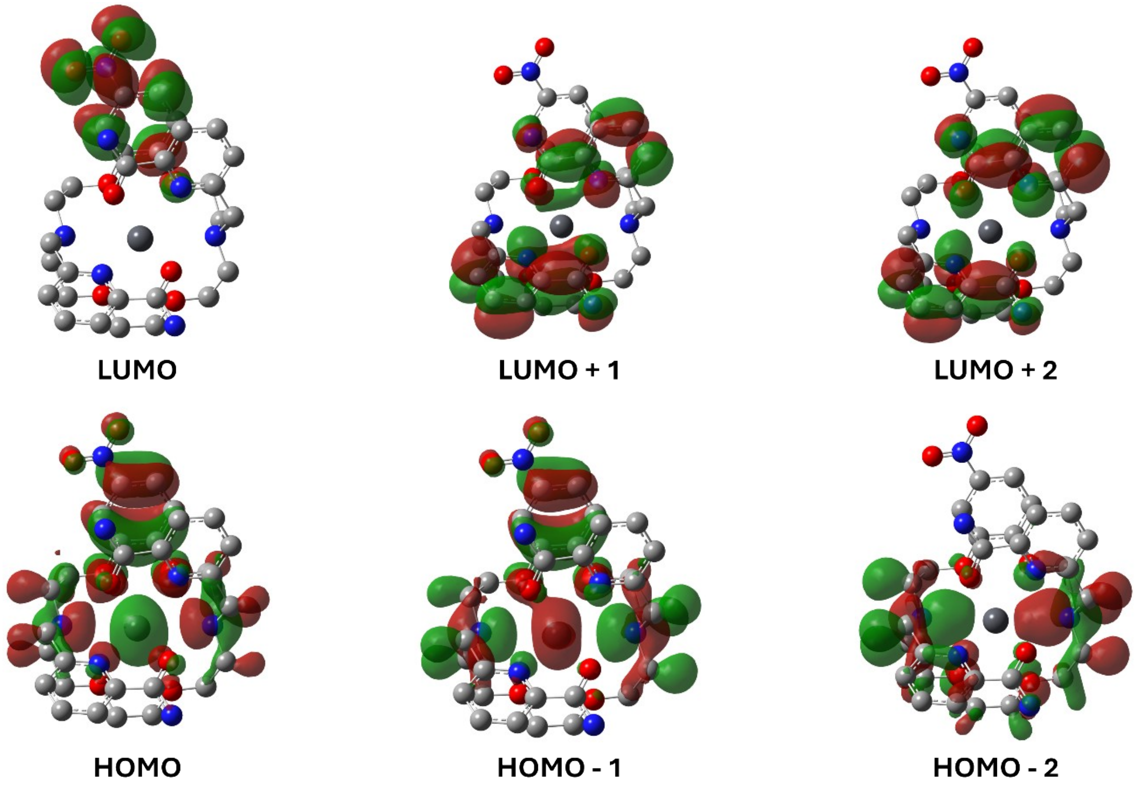

Figure S44. Representation of the HOMO, HOMO-1, HOMO-2, LUMO, LUMO+1, and LUMO+2 of ***syn*-[Pb(NPK_PicAm)]**$^{2+}$.

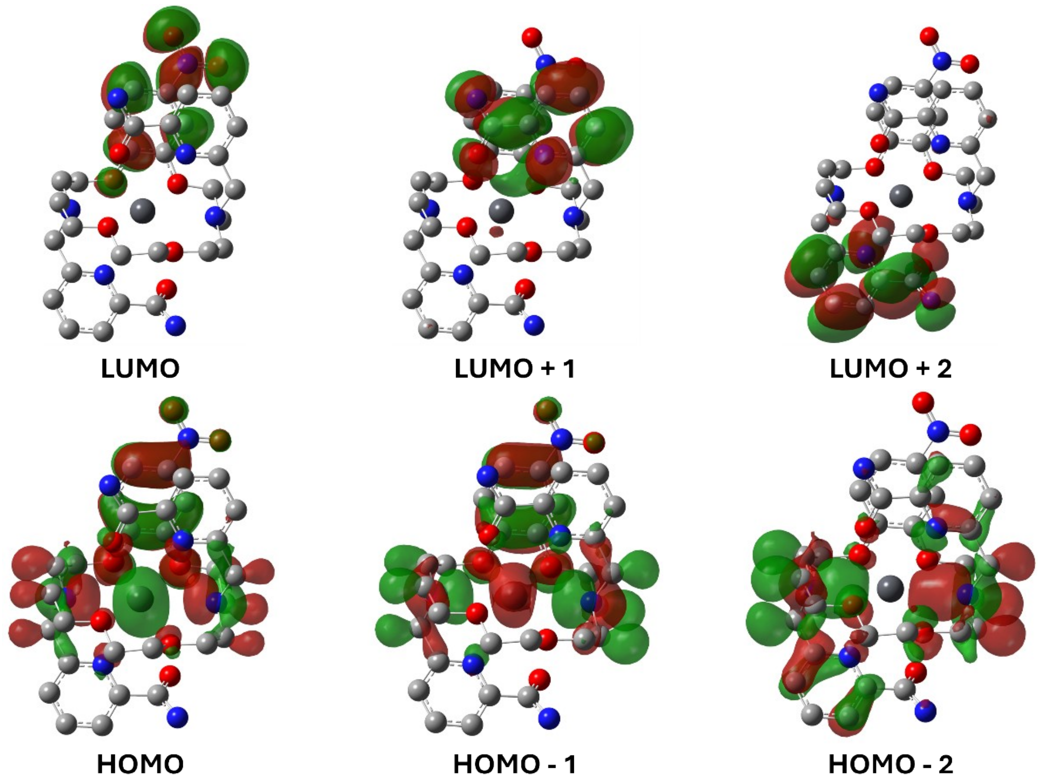

Figure S45. Representation of the HOMO, HOMO-1, HOMO-2, LUMO, LUMO+1, and LUMO+2 of ***anti*-[Pb(NPK_PicAm)]**$^{2+}$.

Table S5. Natural bond orbital analysis of Pb^{2+} $6s^2$ lone pair for **K22_PicAm** Pb^{2+} complexes.

$[Pb(K22\_PicAm)]^{2+}$	Occupancy	Coefficients/Hybrids
Syn-conformer	1.98196	s (99.16 %), p (0.59 %)
Anti-conformer	1.98334	s (99.85 %), p (0.15 %)

Table S6. Natural bond orbital analysis of Pb^{2+} $6s^2$ lone pair for **NPK_PicAm** Pb^{2+} complexes.

$[Pb(NPK\_PicAm)]^{2+}$	Occupancy	Coefficients/Hybrids
Syn-conformer	1.98318	s (99.40 %), p (0.60 %)
Anti-conformer	1.98376	s (99.79 %), p (0.20 %)

Table S7. Cartesian coordinates (Å) of the optimized ligand structure of **K22_PicAm**.

C	2.979278	2.579560	20.500602
C	2.928686	1.069105	20.447513
C	2.526068	0.363843	21.572053
H	2.250059	0.905318	22.465578
C	2.497995	-1.015330	21.492050
H	2.192051	-1.610576	22.343130
C	2.872825	-1.626568	20.305874
H	2.867750	-2.704929	20.210166
C	3.262947	-0.839423	19.229908
C	3.681093	-1.440181	17.910860
H	3.489882	-2.526659	17.922747
H	3.038159	-1.015557	17.139262
C	5.310509	-1.197569	16.144926
H	4.800906	-2.053501	15.673912
H	6.377305	-1.320514	15.976304
C	4.873336	0.085706	15.463421
H	3.803650	0.278668	15.631509
H	5.430723	0.930217	15.889017
C	4.584868	1.002186	13.323252
H	4.796416	1.977810	13.781420
H	3.492950	0.891247	13.252763
C	5.186932	0.973638	11.942253
H	5.195373	-0.060221	11.572583
H	4.571977	1.575686	11.257483
C	7.229856	1.324236	10.825204
H	6.715562	1.825788	9.991643
H	7.324446	0.259006	10.576616
C	5.982755	-1.927146	18.380614
H	6.081194	-2.958990	18.002785
H	5.565757	-1.996991	19.388042
N	3.432577	3.149821	19.368028
H	3.512266	4.148218	19.321137
H	3.707494	2.559824	18.601266
N	3.282964	0.487781	19.308946
N	5.062302	-1.140299	17.578682
O	2.639176	3.192597	21.492144

O	5.132471	-0.037108	14.087975
O	6.489472	1.489424	12.007557
C	11.614993	-2.579543	8.857761
C	11.665586	-1.069088	8.910858
C	12.068209	-0.363821	7.786323
H	12.344223	-0.905290	6.892797
C	12.096278	1.015353	7.866332
H	12.402226	1.610603	7.015257
C	11.721440	1.626585	9.052508
H	11.726514	2.704945	9.148221
C	11.331314	0.839434	10.128469
C	10.913163	1.440186	11.447518
H	11.104371	2.526665	11.435635
H	11.556097	1.015562	12.219116
C	9.283747	1.197564	13.213450
H	9.793348	2.053495	13.684466
H	8.216950	1.320506	13.382071
C	9.720921	-0.085713	13.894953
H	10.790607	-0.278674	13.726865
H	9.163534	-0.930223	13.469356
C	10.009387	-1.002197	16.035121
H	9.797836	-1.977820	15.576952
H	11.101305	-0.891260	16.105609
C	9.407325	-0.973650	17.416120
H	9.398886	0.060209	17.785791
H	10.022278	-1.575700	18.100889
C	7.364401	-1.324244	18.533171
H	7.878695	-1.825797	19.366732
H	7.269814	-0.259013	18.781759
C	8.611501	1.927143	10.977762
H	8.513058	2.958986	11.355592
H	9.028499	1.996989	9.970335
N	11.161685	-3.149809	9.990328
H	11.081996	-4.148207	10.037214
H	10.886762	-2.559816	10.757091
N	11.311300	-0.487769	10.049425
N	9.531955	1.140298	11.779693
O	11.955103	-3.192574	7.866219
O	9.461784	0.037098	15.270399
O	8.104782	-1.489432	17.350818

Table S8. Cartesian coordinates (Å) of the optimized ligand structure of **NPK_PicAm**.

C	4.463300	2.141139	21.594143
C	3.550868	1.075362	21.049356
C	2.454817	0.663445	21.781542
H	2.234909	1.109786	22.740624
C	1.663245	-0.344399	21.248536
H	0.798048	-0.705678	21.789221
C	2.002416	-0.887159	20.026719
H	1.415500	-1.684769	19.589662
C	3.120264	-0.399642	19.349237
C	3.455162	-0.956402	17.986103
H	3.162805	-2.017936	17.967441

H	2.802135	-0.450555	17.271707
C	5.001991	-1.077697	16.154102
H	4.592227	-2.070354	15.907042
H	6.068344	-1.105022	15.938167
C	4.382429	-0.035122	15.241622
H	3.287702	-0.085419	15.247294
H	4.676652	0.971234	15.563424
C	4.232918	0.549755	12.966703
H	4.229035	1.591193	13.310992
H	3.192807	0.237862	12.814090
C	4.980967	0.449621	11.666494
H	5.106159	-0.603864	11.386979
H	4.402744	0.944487	10.876265
C	7.105409	0.860703	10.715966
H	6.692599	1.366222	9.834108
H	7.172047	-0.209465	10.486839
C	5.734855	-1.579232	18.400937
H	5.879332	-2.581352	17.973569
H	5.278506	-1.725181	19.379988
N	5.544155	2.394722	20.857593
H	6.204502	3.091351	21.159305
H	5.691057	1.884399	20.001434
N	3.871363	0.565326	19.859713
N	4.836120	-0.774234	17.572695
O	4.210847	2.718570	22.652889
O	4.855970	-0.277191	13.930367
O	6.241251	1.066720	11.817870
C	12.721553	-1.946739	8.573255
C	12.359231	-0.527691	8.923572
C	12.670446	0.494074	8.042418
H	13.169897	0.277296	7.109263
C	12.324101	1.786438	8.396981
H	12.547561	2.615841	7.738715
C	11.685391	1.999139	9.604720
H	11.394798	2.994564	9.913754
C	11.409097	0.914300	10.429970
C	10.727789	1.101585	11.758663
H	10.734033	2.169921	12.018814
H	11.322266	0.584805	12.512261
C	8.917752	0.458570	13.178157
H	9.210013	1.344406	13.762192
H	7.832908	0.407473	13.197936
C	9.440379	-0.805711	13.838260
H	10.526770	-0.899387	13.800367
H	9.010471	-1.680668	13.354425
C	7.076578	-0.940232	18.667502
H	7.607843	-1.503680	19.440628
H	6.938346	0.088332	19.011833
C	8.482599	1.423359	10.996714
H	8.376499	2.420964	11.452346
H	8.959973	1.571255	10.026991
N	12.361975	-2.866810	9.466132
H	12.569533	-3.836890	9.297287
H	11.880475	-2.585790	10.304165
N	11.748198	-0.326501	10.088703

N	9.363942	0.569336	11.793372
O	13.309887	-2.212108	7.523927
O	7.845737	-0.948052	17.467947
C	9.602508	-0.173206	16.147287
C	8.982680	-0.230413	17.418808
C	10.762272	0.567838	15.976679
C	9.549116	0.425090	18.487614
C	11.330191	1.235841	17.049216
H	11.230190	0.639936	15.005734
C	10.721170	1.151443	18.283214
H	9.104486	0.385588	19.469299
H	12.232635	1.812525	16.914760
O	8.983764	-0.877969	15.189718
N	11.309339	1.843823	19.410739
O	12.333327	2.483618	19.236791
O	10.760128	1.763207	20.497180

Table S9. Cartesian coordinates (Å) of the optimized complex structure of ***syn*-[Pb(K22_PicAm)]**$^{2+}$.

Pb	4.918596	8.694108	5.032714
O	7.435448	10.187059	5.729931
N	3.432308	7.693166	7.424520
O	6.258446	8.566469	7.680709
N	6.424880	10.296344	2.976596
O	2.476704	10.123111	6.071488
N	7.090114	7.524880	3.778620
O	3.628034	11.032057	3.684886
N	2.625755	7.223308	4.637838
C	5.454307	11.016297	2.137089
H	5.970847	11.667779	1.416910
H	4.899223	10.270348	1.570182
C	4.448408	11.858573	2.884944
H	4.921168	12.627534	3.506090
H	3.835129	12.382500	2.142206
C	2.627570	11.765666	4.362174
H	2.029268	12.340391	3.645608
H	3.086977	12.468482	5.067204
C	1.725586	10.816976	5.098650
H	0.924772	11.394448	5.573880
H	1.264754	10.103573	4.401814
C	1.668723	9.437010	7.001505
H	0.988504	8.752918	6.481531
H	1.046908	10.150576	7.554449
C	2.553212	8.720677	7.993259
H	3.175291	9.476683	8.469848
H	1.912113	8.288245	8.775979
C	4.408754	7.251400	8.433904
H	4.925968	6.382467	8.029460
H	3.898476	6.937889	9.356347
C	5.459190	8.269703	8.806957
H	5.028873	9.192110	9.212848
H	6.079798	7.832450	9.597883
C	7.308686	9.460924	7.988235
H	7.909817	9.068108	8.816404

H	6.900507	10.432579	8.290557
C	8.190505	9.626708	6.783683
H	9.023755	10.286428	7.049899
H	8.605916	8.658633	6.473323
C	8.238434	10.675905	4.678920
H	8.893690	9.882944	4.302283
H	8.886068	11.480428	5.046348
C	7.350768	11.247570	3.599762
H	6.764815	12.043054	4.057603
H	7.993335	11.715761	2.838927
C	7.123063	9.324265	2.138772
H	7.861694	9.818367	1.492745
H	6.381387	8.863172	1.483818
C	7.791029	8.179619	2.860138
C	9.069863	7.767501	2.493193
H	9.619912	8.316963	1.740978
C	9.626121	6.666053	3.108188
H	10.626403	6.339463	2.856490
C	8.878955	5.976269	4.050911
H	9.308317	5.113748	4.540399
C	7.604852	6.431324	4.341429
C	2.681345	6.524495	6.975279
H	1.950752	6.209685	7.733142
H	3.392029	5.704150	6.857674
C	1.981544	6.644745	5.644048
C	0.728652	6.062147	5.465003
H	0.223270	5.599342	6.302104
C	0.149892	6.077895	4.214558
H	-0.818895	5.625724	4.048573
C	0.835924	6.672802	3.166168
H	0.405503	6.656388	2.175160
C	2.071974	7.237292	3.424142
C	2.940672	7.853054	2.355855
C	6.661711	5.719481	5.278493
N	7.010671	4.508049	5.715392
H	7.819445	4.016293	5.384321
H	6.371553	4.011172	6.312397
N	2.394467	8.088847	1.161212
H	1.429233	7.916720	0.951716
H	2.978649	8.451723	0.427456
O	4.118950	8.108262	2.585799
O	5.597839	6.235580	5.610730

Table S10. Cartesian coordinates (Å) of the optimized complex structure of ***anti*-[Pb(K22_PicAm)]$^{2+}$**.

C	6.210078	2.628155	16.989725
C	5.552868	1.477188	17.702504
C	5.196369	1.521107	19.039203
H	5.425299	2.375795	19.660647
C	4.556784	0.417971	19.584594
H	4.268241	0.410505	20.627378
C	4.292570	-0.665425	18.772978
H	3.781433	-1.536367	19.161704
C	4.703853	-0.632781	17.442677

C	4.357308	-1.762163	16.506538
H	4.013956	-2.626655	17.089635
H	3.492522	-1.420531	15.935678
C	4.836125	-2.693138	14.337375
H	4.074274	-3.455083	14.554944
H	5.637876	-3.179430	13.785125
C	4.211185	-1.652234	13.436525
H	3.744463	-2.167327	12.589885
H	3.423295	-1.083023	13.943871
C	4.661319	0.235729	12.101694
H	3.970996	0.876533	12.662382
H	4.107321	-0.235120	11.282278
C	5.776074	1.061284	11.521538
H	6.464421	0.433023	10.941490
H	5.339070	1.806250	10.848583
C	7.293843	2.771720	12.120452
H	6.657290	3.607123	11.810957
H	7.868527	2.455440	11.243205
C	6.312208	-3.144188	16.178892
H	6.966417	-3.519045	15.394334
H	5.741436	-3.994612	16.579688
N	6.066527	3.844937	17.524580
H	6.475296	4.632144	17.050107
H	5.443945	4.029506	18.289672
N	5.337774	0.413174	16.929318
N	5.413116	-2.152078	15.575771
O	6.841896	2.442137	15.956451
O	5.200560	-0.756759	12.956655
O	6.481345	1.700001	12.569230
C	8.396584	-2.610303	12.474940
C	8.903352	-1.447811	11.665130
C	9.103703	-1.505925	10.296779
H	8.846245	-2.384602	9.721541
C	9.621939	-0.386471	9.662716
H	9.786158	-0.388178	8.593237
C	9.931406	0.724187	10.420605
H	10.357205	1.606390	9.960671
C	9.681283	0.702122	11.790456
C	10.091016	1.856882	12.669266
H	10.331790	2.725866	12.043804
H	11.029857	1.555923	13.137397
C	9.823643	2.680632	14.922015
H	10.592553	3.427293	14.679098
H	9.085754	3.163781	15.559911
C	10.475308	1.572938	15.718833
H	11.047580	2.025217	16.535689
H	11.174956	0.982907	15.114750
C	10.019537	-0.305167	17.080366
H	10.613476	-1.000068	16.475656
H	10.669510	0.131255	17.846231
C	8.893683	-1.036951	17.758588
H	8.305844	-0.351727	18.381851
H	9.319797	-1.812191	18.403585
C	7.184081	-2.607919	17.290733
H	7.770103	-3.438537	17.698044

H	6.597117	-2.185579	18.113367
C	8.193267	3.237957	13.240818
H	7.570267	3.531286	14.083820
H	8.729499	4.131448	12.888233
N	9.155527	-0.358213	12.388900
N	9.133853	2.215257	13.712443
O	9.482403	0.715964	16.259124
O	8.062834	-1.623270	16.773621
Pb	7.290262	0.026139	14.684862
O	7.876793	-2.427936	13.569433
N	8.549617	-3.833100	11.955948
H	8.256861	-4.627546	12.499353
H	9.098806	-4.004551	11.133531

Table S11. Cartesian coordinates (Å) of the optimized complex structure of ***syn*-[Pb(NPK_PicAm)]$^{2+}$**.

Pb	5.143018	8.684554	5.049150
O	7.668822	10.042148	5.899583
N	3.472825	7.750750	7.345875
O	6.412676	8.230797	7.661868
N	6.577604	10.449692	3.183566
O	2.421415	10.262115	6.059650
N	7.222283	7.654235	3.718342
O	3.872362	11.324295	4.250438
N	2.702282	7.413083	4.531001
C	5.595148	11.304735	2.498995
H	6.100581	12.009438	1.822271
H	4.962473	10.661409	1.886252
C	4.708936	12.124778	3.412007
H	5.283831	12.739205	4.101357
H	4.103491	12.799849	2.806229
C	1.748244	9.578456	7.116881
H	1.017550	8.879056	6.715769
H	1.214705	10.310489	7.726328
C	2.769987	8.876021	7.974786
H	3.507891	9.612261	8.292176
H	2.239297	8.531528	8.875207
C	4.386890	7.137541	8.322574
H	4.791208	6.228170	7.877426
H	3.835165	6.843666	9.227811
C	5.550947	7.992534	8.759123
H	5.233503	8.945957	9.193857
H	6.092701	7.442640	9.537448
C	7.550871	8.987030	8.025545
H	8.145074	8.438213	8.765165
H	7.238541	9.938430	8.472080
C	8.396726	9.242904	6.812266
H	9.308159	9.763045	7.127225
H	8.686767	8.298681	6.333672
C	8.469164	10.534973	4.842877
H	9.028951	9.716274	4.381249
H	9.203934	11.245883	5.238213
C	7.594826	11.268912	3.854624
H	7.095644	12.066085	4.402138

H	8.248480	11.745826	3.110486
C	7.187311	9.558765	2.196473
H	7.917870	10.099354	1.581901
H	6.399868	9.208666	1.527384
C	7.846169	8.324336	2.757303
C	9.048182	7.865497	2.225788
H	9.535780	8.426804	1.440367
C	9.604036	6.705391	2.720865
H	10.538692	6.330962	2.325345
C	8.952066	6.019711	3.735549
H	9.369312	5.102183	4.124671
C	7.757465	6.532510	4.202272
C	2.552707	6.719529	6.870370
H	1.746587	6.544470	7.594423
H	3.112090	5.784529	6.795650
C	1.940543	6.938878	5.507757
C	0.620180	6.557816	5.277184
H	0.020589	6.183533	6.096002
C	0.098844	6.668538	4.007221
H	-0.922795	6.377367	3.802992
C	0.900331	7.164848	2.990401
H	0.503439	7.249076	1.989273
C	2.195306	7.533018	3.301161
C	3.163923	8.068497	2.282838
C	6.917054	5.851693	5.247829
N	7.485408	4.931167	6.010993
H	8.461061	4.697896	5.944517
H	6.923999	4.467348	6.707976
N	2.723797	8.317198	1.055318
H	1.748242	8.293944	0.813335
H	3.367686	8.718461	0.392022
O	4.348735	8.241084	2.586827
O	5.722660	6.153664	5.367054
C	1.783543	10.516698	4.910176
C	2.597331	11.057841	3.885892
C	0.440809	10.270361	4.669988
C	2.078115	11.237708	2.626686
C	-0.090846	10.475999	3.408211
H	-0.200197	9.900811	5.455749
C	0.736879	10.933394	2.405748
H	2.690206	11.585804	1.809747
H	-1.130521	10.261636	3.212806
N	0.209254	11.080088	1.065492
O	-0.980056	10.885864	0.879674
O	0.977067	11.381724	0.166989

Table S12. Cartesian coordinates (Å) of the optimized complex structure of ***anti*-[Pb(NPK_PicAm)]$^{2+}$**.

C	6.750043	2.754534	16.770292
C	5.775920	1.748060	17.311624
C	5.090361	1.916753	18.499964
H	5.236634	2.781539	19.130752
C	4.214865	0.917321	18.897667
H	3.671657	1.007726	19.828720

C	4.064223	-0.196165	18.100495
H	3.400435	-1.001624	18.384643
C	4.798887	-0.290318	16.920911
C	4.613749	-1.469932	16.002801
H	4.122711	-2.282461	16.551264
H	3.908534	-1.149001	15.234861
C	5.535626	-2.501946	14.029532
H	4.697619	-3.210244	14.083199
H	6.409026	-3.062532	13.701174
C	5.197667	-1.468719	12.980521
H	4.908129	-1.989660	12.062343
H	4.359308	-0.833521	13.283993
C	6.033564	0.405235	11.802410
H	5.286713	1.072979	12.245058
H	5.626558	-0.009290	10.874968
C	7.291501	1.159198	11.477367
H	8.039952	0.492383	11.033619
H	7.048195	1.942257	10.752889
C	8.927164	2.599545	12.398755
H	8.560616	3.539823	11.975459
H	9.576818	2.134511	11.653907
C	6.523158	-2.958626	16.164408
H	7.405060	-3.288503	15.614125
H	5.876053	-3.835148	16.312529
N	6.791842	3.960940	17.321368
H	7.446040	4.633296	16.953609
H	6.162447	4.255176	18.047371
N	5.634560	0.666480	16.542772
N	5.834353	-1.944570	15.354718
O	7.483691	2.458808	15.821445
O	6.326882	-0.647318	12.710018
O	7.819823	1.744014	12.659428
C	8.806487	-2.830565	12.396450
C	9.693389	-1.710258	11.920259
C	10.189225	-1.683358	10.628202
H	9.914181	-2.440682	9.907453
C	11.078267	-0.676425	10.287465
H	11.493811	-0.627224	9.289669
C	11.427431	0.255179	11.242043
H	12.115853	1.057659	11.013842
C	10.872899	0.162692	12.515351
C	11.297559	1.122894	13.596751
H	11.931739	1.902003	13.157411
H	11.931989	0.549918	14.272895
C	10.689901	2.128975	15.702676
H	11.612945	2.720397	15.624911
H	9.930733	2.764915	16.157226
C	10.973601	0.967852	16.629898
H	11.441816	1.332584	17.542797
H	11.649455	0.241426	16.183020
C	6.943146	-2.493459	17.538604
H	7.348780	-3.339378	18.096561
H	6.092300	-2.101751	18.093138
C	9.669966	2.909484	13.677054
H	8.992280	3.415312	14.361084

H	10.477223	3.610779	13.423631
N	10.026581	-0.807260	12.844407
N	10.211678	1.731501	14.369346
O	7.962233	-1.500042	17.406483
C	9.110151	0.416876	18.080593
C	8.126127	-0.559812	18.364448
C	9.281800	1.497366	18.928856
C	7.402319	-0.477283	19.530043
C	8.524679	1.603938	20.083892
H	9.991870	2.274953	18.690469
C	7.610876	0.612817	20.369391
H	6.669917	-1.221482	19.796670
H	8.647384	2.451892	20.740159
O	9.796876	0.208061	16.942223
N	6.798806	0.721848	21.565623
O	6.988605	1.662875	22.316718
O	5.947846	-0.126587	21.770543
Pb	7.872481	0.070945	14.786429
N	7.993744	-3.378061	11.490145
H	7.386556	-4.130182	11.773888
H	7.867702	-2.975886	10.577367
O	8.873826	-3.228270	13.557715

12.References

1 A. Roca-Sabio, M. Mato-Iglesias, D. Esteban-Gómez, É. Tóth, A. de Blas, C. Platas-Iglesias and T. Rodríguez-Blas, Macrocyclic Receptor Exhibiting Unprecedented Selectivity for Light Lanthanides, *J. Am. Chem. Soc.*, 2009, **131**, 3331–3341.

2 K. J. Kadassery and J. J. Wilson, WO 2022/251496 A1, 2022.

3 M. Regueiro-Figueroa, J. L. Barriada, A. Pallier, D. Esteban-Gómez, A. de Blas, T. Rodríguez-Blas, É. Tóth and C. Platas-Iglesias, Stabilizing Divalent Europium in Aqueous Solution Using Size-Discrimination and Electrostatic Effects, *Inorg. Chem.*, 2015, **54**, 4940–4952.

4 P. Gans, A. Sabatini and A. Vacca, Investigation of equilibria in solution. Determination of equilibrium constants with the HYPERQUAD suite of programs, *Talanta*, 1996, **43**, 1739–1753.

5 Gaussian, https://gaussian.com/citation/, (accessed 11 March 2025).

6 M. K. Blei, L. Waurick, F. Reissig, K. Kopka, T. Stumpf, B. Drobot, J. Kretzschmar and C. Mamat, Equilibrium Thermodynamics of Macropa Complexes with Selected Metal Isotopes of Radiopharmaceutical Interest, *Inorg. Chem.*, 2023, **62**, 20699–20709.

7 J.-D. Chai and M. Head-Gordon, Long-range corrected hybrid density functionals with damped atom–atom dispersion corrections, *Physical Chemistry Chemical Physics*, 2008, **10**, 6615–6620.

8 J.-D. Chai and M. Head-Gordon, Systematic optimization of long-range corrected hybrid density functionals, *J. Chem. Phys.*, 2008, **128**, 084106.

9 F. Weigend and R. Ahlrichs, Balanced basis sets of split valence, triple zeta valence and quadruple zeta valence quality for H to Rn: Design and assessment of accuracy, *Physical Chemistry Chemical Physics*, 2005, **7**, 3297–3305.

10 W. Küchle, M. Dolg, H. Stoll and H. Preuss, Ab initio pseudopotentials for Hg through Rn, *Mol. Phys.*, 1991, **74**, 1245–1263.

11 A. V Marenich, C. J. Cramer and D. G. Truhlar, Universal Solvation Model Based on Solute Electron Density and on a Continuum Model of the Solvent Defined by the Bulk Dielectric Constant and Atomic Surface Tensions, *J. Phys. Chem. B*, 2009, **113**, 6378–6396.

12 J. K. B. A. E. R. C. E. , J. A. B. C. M. and F. W. Glendening, NBO 6.0: Natural Bond Orbital Analysis program, 2013, preprint.

13 G. Luchini, J. V. Alegre-Requena, I. Funes-Ardoiz and R. S. Paton, GoodVibes: automated thermochemistry for heterogeneous computational chemistry data, *F1000Res.*, 2020, **9**, 291.
